# Accurate and affordable detection of rifampicin and isoniazid resistance in *Tuberculosis* sputum specimens by multiplex PCR-multiple probes melting analysis

**DOI:** 10.1007/s15010-024-02295-w

**Published:** 2024-06-17

**Authors:** Long Xie, Xiao-Ya Zhu, Li Xu, Xiao-Xie Xu, Ze-Fan Ruan, Ming-Xiang Huang, Li Chen, Xi-Wen Jiang

**Affiliations:** 1 Clinical and Translational Research Institute, Faculty of Medical Sciences, Newcastle University, Newcastle Upon Tyne, UK; 2 State Key Laboratory of Virology, School of Life Sciences, Wuhan University, Wuhan, China; 3 Research Institute, DAAN Gene Co., Ltd., Guangzhou, China; 4 The Medicine and Biological Engineering Technology Research Centre of the Ministry of Health, Guangzhou, China; 5 Fuzhou Pulmonary Hospital and Fujian Medical University Clinical Teaching Hospital, Fuzhou, China; 6 Chaoshan Hospital, The First Affiliated Hospital of Jinan University, Chaozhou, China; 7 Department of Pulmonary and Critical Care Medicine, The First Affiliated Hospital of Jinan University, Guangzhou, China; 8 School of Life Sciences and Biopharmaceutics, Guangdong Pharmaceutical University, Guangzhou, China

**Keywords:** *Mycobacterium tuberculosis*, Rifampicin and isoniazid resistance, Multiplex fluorescence PCR, Multiple probes melting analysis, Sputum detection

## Abstract

**Background:**

Escalating cases of multidrug-resistant tuberculosis (MDR-TB) pose a major challenge to global TB control efforts, necessitating innovative diagnostics to empower decentralized detection of gene mutations associated with resistance to rifampicin (RIF) and isoniazid (INH) in *Mycobacterium tuberculosis* (*M. tuberculosis*) in resource-constrained settings.

**Methods:**

Combining multiplex fluorescent PCR and Multiple Probes Melting Analysis, we identified mutations in the *rpoB*, *katG*, *ahpC* and *inhA* genes from sputum specimens. We first constructed a reference plasmid library comprising 40 prevalent mutations in the target genes’ resistance determining regions and promoters, serving as positive controls. Our assay utilizes a four-tube asymmetric PCR method with specifically designed molecular beacon probes, enabling simultaneous detection of all 40 mutations. We evaluated the assay’s effectiveness using DNA isolated from 50 clinically confirmed *M. tuberculosis* sputum specimens, comparing our results with those obtained from Sanger sequencing and retrospective validation involving bacteriological culture and phenotypic drug susceptibility testing (pDST). We also included the commercial Xpert MTB/RIF assay for accuracy comparison.

**Results:**

Our data demonstrated remarkable sensitivity in detecting resistance to RIF and INH, achieving values of 93.33% and 95.24%, respectively, with a specificity of 100%. The concordance between our assay and pDST was 98.00%. Furthermore, the accuracy of our assay was comparable to both Sanger sequencing and the Xpert assay. Importantly, our assay boasts a 4.2-h turnaround time and costs only $10 per test, making it an optimal choice for peripheral healthcare settings.

**Conclusion:**

These findings highlight our assay’s potential as a promising tool for rapidly, accurately, and affordably detecting MDR-TB.

**Supplementary Information:**

The online version contains supplementary material available at 10.1007/s15010-024-02295-w.

## Introduction

Tuberculosis (TB) remains a pervasive and deadly infectious disease worldwide, attributable to *Mycobacterium tuberculosis* (*M. tuberculosis*) infection, as highlighted in the World Health Organization’s (WHO) Global Tuberculosis Report [1]. Historically, anti-TB medications, including rifampin (RIF) and isoniazid (INH), the two first-line drugs most utilized in developing countries, significantly reduced TB morbidity and mortality [2]. However, the emergence and spread of numerous drug-resistant (DR) TB strains, notably multidrug-resistant TB (MDR-TB), characterized by resistance to both RIF and INH, have exacerbated the TB incidence, undermining prior progress [3]. Beyond RIF-resistant (RR) or INH-resistant TB or MDR-TB, TB resistant to additional drugs is categorized as pre-extensively drug-resistant TB (pre-XDR-TB) and XDR-TB. Pre-XDR-TB entails resistance to RIF and any fluoroquinolone, while XDR-TB denotes resistance to RIF, any fluoroquinolone (FQ), and at least one additional group A drug (bedaquiline [BDQ] and linezolid [LZD]) [1]. Despite global efforts to decrease TB cases, DR TB negatively impacts control progress, with MDR-TB remaining undetected in an estimated 60% of TB patients by 2016, increasing annually [1, 4, 5]. Recent data indicates China had approximately 780,000 new TB cases in 2021, representing 7.4% of the global burden. Of these, around 33,000 patients had MDR-TB, ranking China the third-highest TB-burdened country, behind India and Indonesia [6].

China’s 13th Five-Year Plan sets ambitious goals, aiming to reduce TB incidence by 80% and mortality rates by 90% by 2030 [7]. However, with emerging MDR-TB threatening control efforts, rapid laboratory diagnosis and treatment initiation are urgently needed, particularly in underprivileged regions. In response, since 2014 the WHO has established a series of high-priority target product profiles (TPPs), guiding investment, research and development towards optimal diagnostic tools personalized for end users, thereby significantly advancing capabilities [8]. The latest update to these TPPs emphasizes next-generation drug susceptibility testing (DST) in microscopy centres, prioritizing molecular-dominated tools tailored for peripheral centres [9]. The 2021TPP defines desired characteristics, including anti-TB drug selection, analytical performance, operational and infrastructural requirements, and price, setting both optimal and minimal criteria to improve access to TB and DST testing in low- and middle- income countries. By providing simplified, user-friendly, affordable, accurate and reliable test methods in resource-constrained settings, the 2021TPP aims to make TB and DST diagnostics easily accessible to patients [10].

Conventional culture-based DST, e.g. inoculation of *M. tuberculosis* onto 7H10 or 7H11 agar plates with critical concentrations of tested drugs to diagnose resistance by observing a growth rate greater than 1%, is considered the gold standard for diagnosing DR-TB. Nevertheless, it is time-consuming, with a typical culture period of 4–8 weeks [11]. Although commercial automated broth-based systems like the Mycobacterial Growth Indicator Tube (MGIT™) 960 system (supplied by Becton Dickinson, Sparks, MD) can reduce turnaround time, they are costly and require biosafety infrastructure, limiting decentralized utility [11, 12]. Other phenotypic tests, such as microscopic observation of drug susceptibility and thin layer agar assays, are not universally accepted in frontline settings as alternatives due to reliance on subjective individual operator judgment [13].

The molecular mechanism underlying resistance to RIF and INH has been well documented in previous studies. MDR-TB arises from mutations in genes or gene promoters involved in drug activation or encoding drug targets, detectable in drug-resistant isolates. The detection targets for RIF resistance include a segment of the *rpoB* gene, which encodes the β-subunit of the bacterial DNA-dependent RNA polymerase and accumulates mutations within an 81-bp-long RIF resistance-determining region (RRDR) spanning codons 507 to 533, primarily associated with RIF resistance, and up to 98% of RR isolates contain at least one mutation in this region [14, 15]. Regarding INH resistance, mutations occur in the *katG* gene encoding a catalase-peroxidase responsible for INH activation, particularly at codon 315 [16], the promoter region of the *inhA* gene encoding the acyl carrier protein-enoyl reductase involved in mycolic acid synthesis [17], and the regulatory region of the *ahpC* gene, which was previously thought to compensate for the deficiency of *katG* activity [18]. Notably, recent report studying clinical isolates of MDR-TB from the Chinese population has found that the *ahpC*-*oxyR* intergenic region synergistically increases INH resistance level combined with *katG* non-315 mutations [19], with these three genes accounting for the majority of INH resistance. Against this background, nucleic acid amplification tests (NAATs) driven by fluorescence PCR techniques have rapidly emerged as alternative detection approaches.

For over a decade, the WHO has endorsed various molecular tests within the NAAT technology category to detect DR-TB. Prior to 2020, the WHO recommended two specific products, Cepheid’s Xpert MTB/RIF (Sunnyvale, CA) and its successor MTB/RIF Ultra, as well as Molbio Diagnostics’s Truenat MTB-RIF-Dx (Goa, India) [20–22]. However, these tests are primarily design to detect RR-TB and are less suitable for the rapidly evolving global DR-TB landscape. In 2021, the WHO stratified molecular diagnostic technologies by operational complexity and automation levels. Following this approach, Cepheid’s Xpert MTB/XDR, Abbott’s RealTime MTB RIF/INH (Des Plaines, IL), Becton Dickinson’s BD Max MDR-TB (Sparks, MD), Roche Molecular Diagnostics’s cobas MTB-RIF/INH (Pleasanton, CA), and Bruker/Hain Lifescience’s FluoroType MTBDR (Nehren, Germany) received the WHO’s endorsement for that year [23]. It is important to acknowledge the inherent limitations of these commercial testing solutions. They require proprietary polymerase chain reaction (PCR) instruments, limiting interoperability. Moreover, these tests necessitate advanced laboratories with uninterrupted power supply, restricting decentralized use. Furthermore, their limited mutation coverage may miss emerging resistance markers beyond the intended detection range [24]. Additionally, the significant cost associated with each test hinders their widespread adoption in less developed countries and regions. Cepheid is one of the few manufacturers that has agreed a price with the WHO to supply Xpert MTB/RIF or MTB/RIF Ultra to low- and middle- income countries at an ex-factory price of $9.90 per test. In comparison, the negotiated price for Xpert MTB/XDR is $19.80, resulting in RIF/INH resistance detection costing at least $29.70 [22]. Another important consideration is that these tests may erroneously report synonymous or silent mutations as drug resistance [21, 25]. Therefore, there is an urgent need for rapid, accurate, sensitive and affordable diagnostic options for resource-limited peripheral settings.

High-resolution melting curve analysis (HRMA) has emerged as a promising tool for detecting DNA sequence variation in recent years [26]. Advancements in HRMA technique have come from progresses in reaction reagents, transitioning from exclusively using saturating fluorescent dyes (from the first generation LC-Green to current third generation dyes, including LCGreen+, SYTO9, EvaGreen and ResoLight) to the incorporation of unlabelled melting probes, which boost sensitivity and specificity [27]. Nevertheless, fluorescent dye-based HRMA is limited to detecting mutations in one fragment per reaction, and detection conditions must be iteratively optimized against controls, impacting sensitivity and specificity due to incomplete coverage of all crucial mutations in the amplification targets. Molecular beacon-based melting analysis, on the other hand, emerges as a novel genetic mutation analysis technique. It relies on detecting homozygous or heterozygous carriers of a particular allele, as determined by changes in the melting temperature (T_m_) or the visual appearance of a double peak reflecting the shape of the melting curve. This method exhibits characteristics such as sensitivity, rapidity, accuracy, affordability, and the ability to identify diverse mutations without constraint by specific detection sites, enabling comprehensive closed-tube analysis [28]. Furthermore, sequence-specific probes labelled with distinct fluorophores facilitate concurrent multi-target detection. Therefore, to accurately and simultaneously detect RIF and INH resistance, a comprehensive HRMA assay should be developed, targeting the rifampicin RRDR of the *rpoB* gene, specific regions of the *katG* and *ahpC* genes, and the *ahpC* and *inhA* promoters.

The primary objective of this study was to develop an innovative diagnostic assay for detecting MDR-TB in patients, tailored for peripheral laboratories and resource-poor settings where traditional culture-based DST is often unavailable. We modified conventional PCR-HRMA by incorporating multiple molecular beacon probes, using the WHO’s updated 2021TPP as a guideline. Our assay utilizes sequence-specific probes with varying melting temperatures to genotype diverse mutations associated with RIF and INH resistance, including point mutations and deletions. The resulting melting curve profiles, arising from differences in the thermodynamic stability of fully complementary and mismatched probe-target sequence duplexes between wild-type (WT) and mutant templates, enabled simultaneous identification of up to 40 commonly reported mutations occurring in the four aforementioned genes. This comprehensive closed-tube approach represents a novel contribution to the field, as multi-probe melting curve analysis has not been extensively documented. Given the combined use of molecular beacons and melting curve analysis with fluorescence PCR, we term this assay PCR-Multiple Probes Melting Curve Analysis (MPMA), differentiating it from traditional dye-dependent HRMA.

## Methods

### Study design

**Fig. 1:**
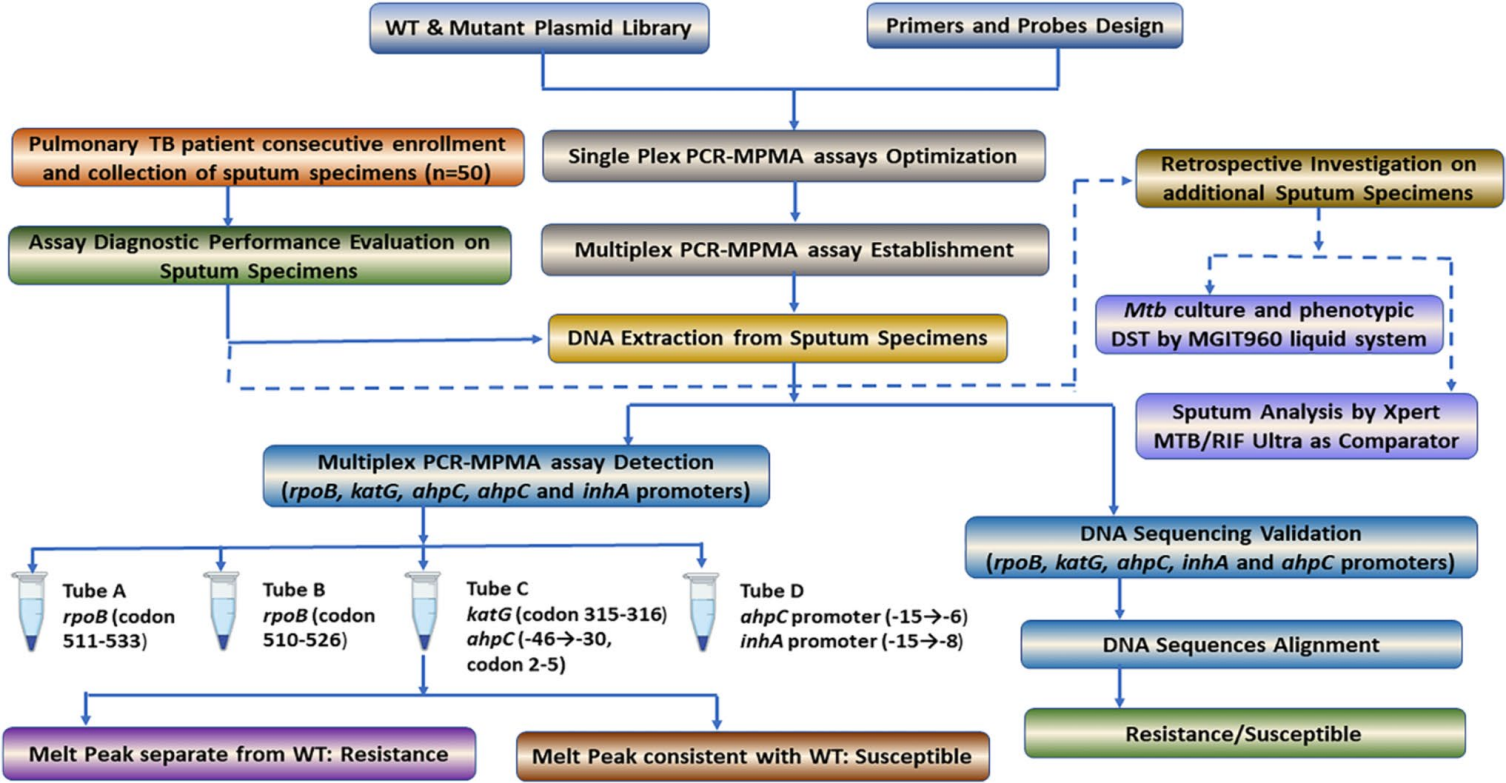
Flowchart describing the present study

This study, consisting of the assay development phase and the subsequent evaluation of diagnostic performance, was conducted at the Medicine and Biological Engineering Technology Research Centre of the Ministry of Health, Guangzhou, China. A systematic approach was employed to select mutation sites associated with resistance to RIF or INH for the four target genes. The selection process involved referencing the WHO guideline for genotypes linked to resistance [29], screening mutation sites detected by the six automated NAAT platforms recommended by the WHO (see Table S1 for a full list in the supplementary material), and conducting an extensive literature review to identify commonly reported mutation sites in Chinese TB patients. Based on this process, we designed the study workflow (depicted in Fig. 1). The experimental process involved several key steps: preparation of a positive control plasmid library, design of primers and probes, optimization and standardization of the multiplex PCR-MPMA assay, evaluation of diagnostic performance using clinical samples, validation of results by DNA sequencing, retrospective validation by bacteriological culture and phenotypical DST, and comparison to data obtained using the Xpert MTB/RIF assay as a WHO-recommended comparator.

### Clinical feasibility evaluation and specimen collection

Our study was conceptualized as a pilot clinical feasibility study, designed to evaluate the prospective efficacy of our newly developed assay. Consequently, the calculation of a sample size was deemed unnecessary. It is pertinent to highlight that feasibility studies commonly involve 10 and 300 participants [30]. Furthermore, evidence suggests that a minimum cohort of 50 is adequate to assess the precision and efficiency of a pilot trial [31]. In this light, between April 2021 and March 2022, fifty patients who have been previously diagnosed with active pulmonary TB at Guangzhou Chest Hospital, a regional specialist TB hospital in Guangdong Province, China, were consecutively recruited for this study. Each patient’s initial diagnosis was confirmed through positive sputum cultures for *M. tuberculosis*, as reported by the hospital’s district referral laboratory.

Recruitment was conducted through a partnership with Chaoshan Hospital, a county-level district healthcare facility in Raoping County, eastern Guangdong, China. As part of the enrolment process, potential participants presented at the Infectious Diseases Outpatient Clinic of Chaoshan Hospital seeking continuation of their TB treatment. During these visits, the hospital staff screened patients for eligibility and willingness to participate. Eligibility criteria included being 20 years of age or older, a prior bacteriological confirmation of TB at least six months ago, absence of anti-TB treatment in the month preceding their current visit, and the presence of clinical symptoms such as persistent cough and expectoration, supported by radiographic evidence of active TB. Additionally, all participants had previously initiated anti-TB treatment after their first diagnosis but had discontinued it on their own accord and were seeking to resume treatment within a month of cessation.

One of the key criteria for inclusion was a positive result for acid-fast bacilli (AFB) staining, reinforcing the active status of the disease. Following informed consent, each patient was asked to provide a substantial sputum sample—collected the morning after their clinic visit—to ensure the largest volume possible. These specimens were collected by specialized personnel at Chaoshan Hospital using sterile 50 ml Falcon tubes. The collected specimens were immediately processed for direct smear microscopy to assess AFB presence, followed by liquefaction and decontamination procedures, all of which were conducted at the Tuberculosis and Respiratory Diseases Laboratory of Chaoshan Hospital.

### Clinical specimen processing

Sputum specimens, each with a minimum volume of approximately 5.0 ml, were handled in adherence to the National guidelines for TB laboratories (Chinese Antituberculosis Association) adopted from WHO recommendations. Upon collection, one loopful (approximately 0.2 ml) of each sputum sample was subjected to direct smear examination using the standard Ziehl Neelsen’s staining method as described elsewhere [32]. Briefly, the sputum specimen was air dried, heat fixed, 0.8% carbol fuschin as the primary stain and 0.06% methylene blue as the counter stain for the presence of AFB. Smear positivity was graded based on the presence of AFB, ranging from scanty (1–8 organisms per 100 fields at 100× magnification) to 4 + (> 10 organisms per field at 100× magnification). The remaining sputum portions were further digested and decontaminated with a twofold volume of 0.5% N-acetyl-l-cysteine/3% sodium hydroxide (NaLC-NaOH) solution for 15 min before the addition of phosphate-buffered saline (PBS) solution to make a total volume of 45 ml, followed by centrifugation for 15 min at 3000×*g*. After removing the supernatant, the pellet was resuspended in 2 ml PBS. These digested and decontaminated samples were transported back to the Medicine and Biological Engineering Technology Research Centre of the Ministry of Health in strict compliance with the national regulations for the transportation of biological substances. Specimens arrived within 48 h of collection and were processed within 12 h of arrival. Specifically, each 1 ml of suspended sputum sediment was divided into 5 tubes of 200 µl each, with one aliquot immediately undergoing DNA extraction and the remaining portions stored at – 80 °C for further testing.

### Preparation of gene mutants plasmid library

To optimize and standardize the multiplex PCR-MPMA assay, mutant and WT controls were obtained by acquiring a commercially engineered plasmid library based on the target gene fragments. The construction of the plasmid library containing 40 mutations based on the above strategy selected as targets was consigned to Sangon Biotech (Shanghai, China). An additional set of 5 plasmids, each carrying a synonymous mutation within the RRDR region of the *rpoB* gene, was procured to evaluate the assay’s ability to identify false positives caused by these silent mutations. Individual mutant plasmids were subsequently constructed using the contractor’s established PCR-principled cloning protocols. Sangon Biotech designed a series of PCR chimeric primer pairs based on the genome sequence of *M. tuberculosis* strain H37Rv (NCBI Genbank Accession Number: NC_000962.3), which contained both the WT and desired mutation sequences. The institution first amplified the long fragment DNA sequences using overlap extension PCR technique as described elsewhere [33]. The genes were inserted into the pUC57-kan vector by homologous recombination and enzymatic ligation and *Escherichia coli* was then transformed to obtain clones. After isolating the finished plasmids, Sangon Biotech verified the integrity of all mutants and WT plasmids through Sanger’s sequencing. The full list of plasmids used in assay development is shown in Supplemental Table S2, and the complete sequence of each plasmid is provided in Supplemental Table S3.

### DNA extraction

Genomic DNA extraction from the digested and decontaminated sputum samples was performed using a Nucleic Acid Isolation Kit and a Smart32 Nucleic Acid Extraction instrument (both supplied by DAAN Gene, Guangzhou, China) following the manufacturer’s instructions. A 200 µl aliquot of the suspended sputum sediment was used for DNA extraction, and the procedure took approximately 40 min. The DNA was eluted in a final volume of 60 µl and stored at – 20 °C until required for further experiments.

### Design of primer and probe for multiplex PCR-MPMA

Nine primer–probe sets were designed for multiplex fluorescent PCR, divided into four tubes to target mutations of interest (Table 1). Specific primers and molecular beacon probes for the selected *rpoB*, *katG*, *inhA* and *ahpC* genes were designed based on DNA sequences from the International Nucleotide Sequence Database Collaboration (INSDC) at the National Centre for Biotechnology Information (NCBI, https://www.insdc.org/), by utilising the oligo primer analysis software Oligo7, version 7.37 (http://oligo.net). The expected amplicon sizes ranged from 82 and 173 bp with detectable melting temperature variation. In specific, a pair of primers designed to detect mutations associated with RIF resistance selectively amplified a 166 bp amplicon covering codons 510–533, the entire 81 bp length of RRDR within the *rpoB*, along with four molecular Beacon probes targeting specific loci to identify nucleotide changes. A variable number of primers were designed to span a region within either the *katG* or *ahpC* or *inhA* promoter known to contain mutations conferring resistance to INH. Following the same rules, different numbers of probes were used to detect mutant loci localized in the respective genes. Each probe was 5’ end labelled with a unique fluorescent report dye including Texas Red, Cy5, and VIC, and 3’ end quenched with Black Hole Quencher 2 (BHQ2). All primers and probes were synthesized by Sangon Biotech (Shanghai, China).

**Table 1:** Primers and probes sequences used in multiplex PCR-MPMA assay

Tube	Primer/probe name^a^	Sequence (5′–3′)	Amplicon size (bp)	%GC content	Annealing temp (℃)	Nucleotide positions^b^
A, B	rpoB-F	5′-CCGCAGACGTTGATCAACATC-3′	169	52.4	61.1	1222 to 1242
A, B	rpoB-R	5′-CGGCACGCTCACGTGACA-3′		66.7	62.6	1387 to 1370
A	rpoB-P1	5′-Texas-Red-GCCAGCTGAGCCAATTCATGGACCAG-BHQ2-3′	/	57.7	73.6	1283 to 1308
A	rpoB-P2	5′-Cy5-GACTGTCGGCGCTGGGG-BHQ2-3′	/	76.5	62.6	1343 to 1359
B	rpoB-P3	5′-Texas-Red-GCTGTCGGGGTTGACCCACAA-BHQ2-3′	/	61.9	67.3	1317 to 1337
B	rpoB-P4	5′-Cy5-TCGGCACCAGCCAATTC-BHQ2-3′	/	58.8	57.8	1274 to 1300
C	katG-F	5′-GAAGAGCTCGTATGGCACCG-3′	85	60.0	60.6	900 to 919
	katG-R	5′-CCATTTCGTCGGGGTGTTCGTC-3′		59.1	68.1	984 to 963
C	katG-P	5′-Texas-Red-GACGCGATCACCAGCGGCATCGAG-BHQ2-3′	/	66.7	76.3	931 to 954
C	ahpC-F-1	5′-CCGGCTAGCACCTCTTGG-3′	203	66.7	58.8	– 88 to – 71
	ahpC-R-1	5′-TGAGCTGGTAGGCGGGGAAT-3′		60.0	64.1	115 to 97
C	ahpC-P1	5′-VIC-CATGCCACTGCTAACCATTGGCGAT-BHQ1-3′	/	63.2	64.0	42 to 66
C	ahpC-P2	5′-Cy5-TGTGATATATCACCTTTGCCTG-BHQ2-3′	/	40.9	55.6	– 49 to – 28
D	ahpC-F-2	5′-TATGGTGTGATATATCACCTTTGC-3′	153	37.5	56.7	– 54 to – 31
	ahpC-R-2	5′-GATGAGAGCGGTGAGCTGGTAGGC-3′		62.5	68.4	99 to 76
D	ahpC-P3	5′-Texas-Red-ACTTCACGGCACGATGGAATGTC-BHQ2-3′	/	52.2	65.8	2 to – 21
D	inhA-F-A2	5′-GCGTAACCCCAGTGCGAAAGTT-3′	126	54.5	65.3	– 102 to – 81
	inhA-R-A2	5′-CCCTTCAGTGGCTGTGGCAG-3′		65.0	63.6	5 to 24
D	inhA-P	5′-Cy5-CGGCGAGACGATAGGTTGT-BHQ2-3′	/	57.9	58.3	− 23 to − 5

^a^*F* forward, *R* reverse, *P* probe

^b^Nucleotide position numbers correspond to the nucleotides of the coding sequences of each gene

### Establishment of the multiplex fluorescent PCR assay

To develop a robust fluorescent PCR assay, we designed a four-tube, one-step, asymmetric PCR system allowing for the simultaneous detection of multiple mutations. This system analyzes nucleic acids from a single human sample distributed across four individual tubes, compatible with mainstream fluorescence PCR instruments, including Roche’s LightCycler 480, Applied Biosystems’ 7500 Fast and Bio-Rad’s CFX96 Deep Well Dx. This setup leverages a uniform PCR reaction across all channels, facilitating simultaneous processing without the need for partitioning experimental time or formats, extending our previous work on detecting nine respiratory pathogens [34]. The tube assignments were as follows: tubes A and B: the most frequent mutations in the *rpoB* gene; tube C: mutated loci in the *katG* and *ahpC* genes; tube D: mutant loci in the *inhA* and *ahpC* promoters. The initial phase involved meticulous optimization of single plex fluorescence PCR-melting curve assays through selecting optimal primers/probe pairs, optimizing PCR thermal cycling parameters, and determining appropriate concentrations of reaction components. This groundwork was integral to the subsequent development of the multiplex PCR-MPMA assay. The reference plasmid library, as described in Supplemental Table S2, harboring 40 control plasmids with single *rpoB*, *katG*, *inhA* and *ahpC* mutations conferring RIF or INH resistance, or WT genotypes from the standard *M. tuberculosis* strain H37Rv, served as DNA templates to develop single plex PCR-MPMA assays that produced distinct melting curves for each target. The achievability of each single plex assay was assessed using tenfold serial dilution series (from 1 × 10^8^ to 1 × 10^4^ copies/ml) of positive mutants and WT nucleic acid standards. Screening identified nine primer/probe pairs with superior performance in post-PCR melting curve analysis, maximally distinguishing WT and variants based on T_m_ values. Rigorous validation over 5 days, with ten repetitions, ensured reproducibility, with the standard deviations (SD) for each T_m_ of not exceeding ± 0.2 °C. These optimized conditions for single plex PCR assays were seamlessly integrated into the multiplex PCR system, ensuring accurate and reliable diagnostic results. The multiplex real-time PCR reaction mixture included the following components: 60 mM Tris–HCl (pH8.8), 2.5 mM MgCl_2_, 20 mM (NH_4_)_2_SO_4_, 2% Tween-20, 200 nM dNTPs, 500 nM forward primer, 5000 nM reverse primer, 1000 nM probe, 3 U Taq Hot-Start DNA polymerase, 0.5 U Uracil-DNA Glycosylase (UDG), 5 µl of the template DNA and diethylpyrocarbonate-treated water for a total reaction volume of 25 µl. PCR amplification was performed on a CFX96 Deep Well Dx system (Bio-Rad Laboratories, Inc., Hercules, CA) under the following conditions: 1 cycle of UDG enzyme treatment at 50 °C for 5 min, 1 cycle of initial denaturation at 95 °C for 5 min, followed by 11 touch down cycles of denaturation at 95 °C for 15 s, annealing at 69 °C for 30 s (with the temperature decreasing by 1 °C per cycle) and extension at 72 °C for 20 s. This was followed by 40 cycles where the annealing temperature stabilized at 58 °C while the other conditions remained the same. A final melting analysis program consisted of 95 °C for 2 min, 40 °C for 2 min and a plate reading step during which the temperature was increased from 40 to 86 °C at a rate of 0.4 °C per second with fluorescence signals acquired in the VIC, Cy5 and Texas-Red after every 1 °C temperature increase. Unless otherwise specified, all samples were simultaneously tested in tubes A, B, C, and D, with the process repeated twice. Each multiplex PCR run included a negative control and a positive control consisting of a mixture of WT plasmids of the *M. tuberculosis* genes *rpoB*, *katG*, *inhA,* and *ahpC,* and 510–512 deletion mutant plasmids of the *rpoB* gene.

### Melting curve analysis

The melting curve analysis followed the thermal cycling conditions and fluorescence acquisition mode described earlier. The analysis was performed using CFX Manager™ software (Bio-Rad Laboratories, Inc.) after completing amplification of a single-stranded oligonucleotide sequence complementary to the designed fluorescent group-labelled probe sequence by the PCR reaction. While monitoring the change in fluorescence value (expressed herein as RFU, relative fluorescence units) in real time, and by plotting the negative derivative of RFU (-*d*RFU) versus temperature (T), the melting curve of the hybridization product of the probe and the template sequence was obtained, and the melting point (T_m_ value) could be derived from the peak value of the -*d*RFU/*d*T versus T curves to obtain the mutation information of the sequence. If the target sequence perfectly matched the probe, the probe hybridized at the highest T_m_. If there was a point mutation, insertion or deletion in the sequence, the probe hybridized at a lower T_m_ than in the previous case. Whether a mutation occurred in the tested sample was determined by comparing the difference in T_m_ values of the melting curves between the measured sample and the positive reference control. In the same reaction tube, similarly consistent T_m_ values of the sample tested and the positive control in all channels identified the WT; while a difference of more than 2 °C (∆T_m_ ≥ 2 °C) of the measured T_m_ distinguished the mutant from the WT signal.

### The absolute quantification of reference plasmids by droplet digital PCR

A droplet digital PCR (ddPCR) technique was utilised to directly and precisely quantify the copy numbers of the reference plasmids. The sequences of the primers and probes used for ddPCR are listed in Supplemental Table S4. Herein, a One-Step ddPCR Advanced kit for Probes (Bio-Rad Laboratories, Hercules, CA) was used according to the manufacturer’s recommendation and as previously described [34]. Briefly, reaction mixtures were assembled with 10 µl ddPCR supermix, ddPCR primers and probes (final concentration of 450 nM and 250 nM, respectively), and 4 µl template nucleic acids in a final volume of 22 µl. Each reaction was loaded into the sample well of an eight-well droplet cartridge with 70 µl of droplet generation oil (Bio-Rad). Following their formation in a QX200 droplet generator (Bio-Rad), the droplets were transferred to a 96-well PCR plate, heat-sealed with foil, and subjected to amplification using a Longene^®^ A200 Gradient Thermal Cycler (Longene Scientific Instrument, Hangzhou, China) with the following PCR parameters: initial denaturation at 95 °C for 10 min, followed by 40 cycles of 95 °C for 30 s and 55 °C for 1 min; and a final extension step at 72 °C for 7 min.

### The evaluation of analytical performance of the multiplex PCR-MPMA assay

The plasmid library consisting of the mutants and WT plasmids as the positive references was used to estimate the sensitivity of the multiplex fluorescence PCR-MPMA assay, particularly the limit of detection (LOD) and the ability to differentiate hetero-resistance. Serial tenfold dilutions of the 44 reference plasmids with known initial concentrations determined by ddPCR were subjected to assay detection to determine assay LOD. In addition, the performance of the assay to discriminate hetero-resistant samples was evaluated by constructing a series of plasmid mixtures containing different proportions of mutant plasmids (20%, 30%, 40%, 50%, and 60%, respectively) against WT plasmid as a background at a concentration of 1 × 10^4^ copies/ml. Furthermore, a variety of bacterial species, consisting of a range of non-tuberculosis mycobacteria (NTM) and common Gram-positive and Gram-negative bacteria found in sputum and the respiratory tract and causing symptoms similar to TB, were used to evaluate the assay’s specificity. These bacteria, which served as negative controls for cross-reactivity validation, including *Mycobacterium xenopi*, *Mycobacterium avium*, *Mycobacterium kansasii*, *Mycobacterium abscessus*, *Mycobacterium gordonae*, *Mycobacterium intracellulare*, *Mycobacterium marinum, Streprococcus pneumoniae, Haemophilus influenzae, Escherichia coli, Staphyolcoccus aureus, Pseudomonas aeruginosa* and *Nocardia *sp., were purchased from the American Type Culture Collection (ATCC, Manassas, VA) in concentrated saturated bacterial liquid forms. A mixture of genomic DNA from each of these microorganisms at known starting concentrations, determined by ddPCR, was tested alongside *M. tuberculosis* nucleic acid to assess non-specific amplification. Evaluation of assay precision (reproducibility of replicate tests) was performed by examining intra-assay and inter-assay coefficient of variation (CV). Intra-assay reproducibility tests were performed in 10 replicates by respectively measuring two different concentration mixtures (5 × 10^6^ and 5 × 10^4^ copies/ml) of each positive control plasmid within the same experiment. Inter-assay variability was measured by repeating the intra-assay experiment on three continuous days to validate the reproducibility of the multiplex fluorescent PCR-MPMA assay. The means of each replicated data points, standard deviation (SD), and CV of the T_m_ values were calculated to verify the precision and repeatability of this assay.

### The assessment of diagnostic performance of the assay

DNA samples extracted from 50 AFB smear-positive sputum specimens were subjected to our multiplex fluorescence PCR for amplification, followed by multiple probe melting curve analysis (MPMA). Nucleic acids from all 50 samples were synchronously analyzed through Sanger sequencing, recognized by the WHO as the reference standard for genotypic drug resistance information. The remaining aliquots of decontaminated sputum sediment suspension from each patient were sent to Fuzhou Pulmonary Hospital in May 2023 for a retrospective evaluation of the multiplex PCR-MPMA assay’s performance in comprehensively assessing MDR-TB in the participants. Prior to transporting the samples, an aliquot of each cryopreserved sample was selected to repeat the multiplex PCR-MPMA assay. Consistent results with the initial run confirmed that prolonged freezing did not adversely affect the sample stability. At Fuzhou Pulmonary Hospital, liquid culture was conducted using 500 µl of each homogenized sputum sample in the automated BACTEC MGIT™ 960 system (Becton Dickinson, Sparks, MD) for up to 6 weeks or until flagged as positive. Positive cultures were then confirmed as *M. tuberculosis* using the immunochromatographic method with the MPT64 antigen detection kit by Bioline TBAgMPT64 (Standard Diagnostics, Seoul, South Korea). Phenotypic DST was subsequently performed using the MGIT™ SIRE kit (Becton Dickinson, Sparks, MD), following manufacturer’s protocol. The critical concentrations of 1.0 µg/ml for RIF and 0.1 µg/ml for INH were utilized to determine the drug susceptibility profile. To compare the results using a WHO-approved molecular test, the Xpert MTB/RIF assay (Cepheid, Sunnyvale, CA) was employed. Approximately 1.0 ml aliquots of each sample were diluted with sample reagent at a ratio of 1:2, as per the manufacturer’s instruction. Following sample preparation, which involved a 15 s vortexing step followed by a 15 min settling period with an additional 15 s intermittent vortex halfway through, a precise volume of the mixed solution was collected using the provided calibrated pipette and transferred into the designated cartridge. These cartridges were then inserted into the Xpert instrument, allowing for automated result generation, which was read after a 90-min reaction period.

### DNA sequencing

Automated sequencing of the four genes, *rpoB*, *katG*, *inhA*, and *ahpC* was performed by commissioning Sangon Biotech (Shanghai, China) using an Applied Biosystems 3730xl DNA Analyzer (Thermo Fisher Scientific, Foster City, CA) according to the contractor’s protocol. Preliminary amplification of the nucleic acids from the 50 clinical samples was performed in our laboratory by conventional PCR using the primer sequences listed in supplementary Table S4 under the following thermal cycling conditions: 95 °C for 15 min, followed by 40 cycles at 94 °C for 30 s, 55 °C for 30 s, and 72 °C for 30 s, with a final extension at 72 °C for 7 min. The sequencing results were confirmed using the BLAST algorithm and aligned with the reference sequence for the *rpoB*, *katG*, *inhA* and *ahpC* (NCBI GenBank accession no. 888164, 885638, 886523 and 885717) to identify the presence or absence of mutations of interest using the MEGA software version 7.0.

### Data analysis

Using established epidemiological methods, the multiplex PCR-MPMA assay was evaluated for diagnostic performance by calculating sensitivity, specificity, positive predictive value (PPV), negative predictive value (NPV) and coincidence with 95% confidence intervals (CIs). The gold reference for this evaluation was bacteriological culture and phenotypic DST. To measure the disparity in performance among various molecular detection techniques in comparison to DST, McNemar’s test (paired Chi-square test) was employed. Sensitivity and specificity values calculated for each test were graphed on a Receiver Operating Characteristic (ROC) plot of sensitivity versus specificity to determine the area under the ROC curve (AUC)-ROC, which assessed the accuracies of the tests being evaluated. Data analysis was conducted using SPSS software, version 21.0 (IBM Corp., Armonk, NY, USA). A p value of < 0.05 was considered statistically significant in detecting accuracy differences between the compared methods.

## Results

### Development of the multiplex PCR-MPMA assay

Following the rigorous optimization of the single plex PCR assays described in the Methods section, we successfully obtained reference melting curve shapes and T_m_ values for our selected targets, which is crucial for establishing the multiplex PCR-MPMA assay. Supplemental Figure S1 shows the melting curves from individual optimized PCR-melting curve assays, while Supplemental Table S5 specifies the T_m_ values for effectively discrimination between WT and mutant samples.

**Fig. 2:**
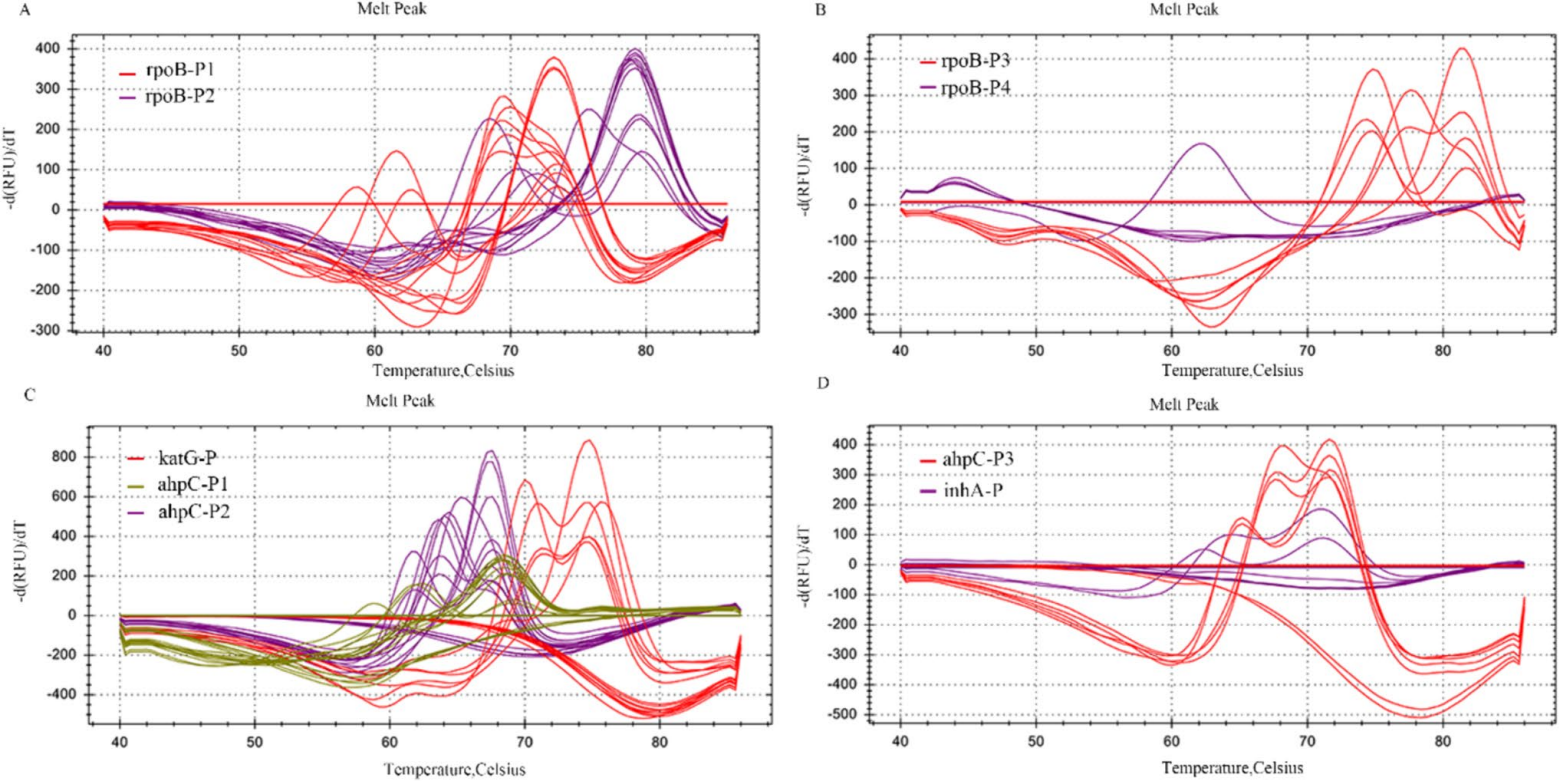
Representative melting curves demonstrating the detection of *M. tuberculosis* RIF (**A**, **B**) and INH (**C**, **D**) resistance by the multiplex PCR-MPMA assay. The melting curve profiles of each of the tubes **A**–**D** of the four-tube multiplex PCR-MPMA assay are shown in **A**–**D**. The individual mutant melting curve profiles were visually easily distinguished from the WT profiles by the distinct differences in the T_m_ values together with the shapes of the melting curves, and no interfering crosstalk from one channel to the other was observed

To establish an integrated multiplex PCR-MPMA system for identifying RIF and INH resistance in *M. tuberculosis*, the optimized single-plex assays were subgrouped into A-, B-, C-, and D-tubes according to the target genes’ detection strategy, using mixtures of WT and mutant plasmids (1 × 10^4^ copies/ml, with 40% WT) for evaluation. This achieved simultaneous genotyping of the *rpoB*, *katG*, *inhA*, and *ahpC* genes (Fig. 2), evaluating whether the multiplex PCR-MPMA assay could simultaneously discriminate between WT and multiple mutant standard references through combining distinct melting temperatures and melting curve peaks generated by fluorescent channel-labelled probes targeting diverse template sequences, reflecting various phenotypes. As indicated in Fig. 1 and Table 1, the melting profile of tube A, which contained two molecular beacon probes covering the *rpoB* point mutations between codons 511–533, revealed a 73.1 ± 1 °C T_m_ value for WT (T_m.W_) reference in the Texas Red fluorescence channel and a 78.9 ± 1 °C T_m.W_ for other WT sequences in the Cy5 channel with corresponding highest melting curve peaks from the probes complementary to the WT sequences, both of which were readily distinguishable from those T_m_ values of mutant sequences (T_m.S_) as the ∆T_m_ ≥ 2 °C. This similar pattern, whereby melting peaks corresponding to the T_m.S_ of the reporter probes collectively help determine template mutations, was observed in the B-, C-, and D-tubes. Specifically, using the probes identical to the WT sequences of *rpoB*, *katG*, *ahpC* and *inhA* generated individual T_m.W_ values of 81.3 ± 1 °C (the Texas Red channel, B-tube), 72.5 ± 1 °C (the Texas Red channel, C-tube), 68.3 ± 1 °C (the VIC channel, C-tube), 66.8 ± 1 °C (the Cy5 channel, C-tube), 72.1 ± 1 °C (the Texas Red channel, D-tube), and 70.1 ± 1 °C (the Cy5 channel, D-tube), respectively, all easily distinguished from those of the mutant sequences. Exceptionally, the probe codenamed *rpoB* P4 for *rpoB* deletion mutants in the B tube produced no WT melting peak, with homozygous/heterozygous mutations in the template generating a mutant melting peak in the Cy5 fluorescence channel. Notably, based on the mutation interpretation guidelines outlined in this optimization experiment, the DNA extracted from the five plasmids carrying synonymous mutations Q510Q, Q513Q, F514F, D516D and L533L (listed in Supplemental Table S2) displayed ∆T_m_ values greater than 5 °C, indicating that all plasmids in the batch were resistant to RIF and, consequently, the presence of false positives (data shown in Supplemental Table S6).

### Evaluation of the accuracy and reproducibility of the multiplex PCR-MPMA assay

The multiplex PCR-MPMA assay’s capacity to predict RIF and INH resistance was evaluated by observing the *rpoB* gene’s RRDR (codon 507–533) and markers in the *katG*, *inhA* and *ahpC* genes that caused differences in T_m.S_ and melting curve shifts. This required an evaluation of the accuracy of the temperature readings indicative of the mutations. To demonstrate high precision, intra-assay repeatability was validated by testing two concentrations (1 × 10^6^ and 1 × 10^4^ copies/ml) of the mutated reference plasmids in ten replicates to determine the CV of the T_m.S_. As delineated in Table 2, for the mutation detection spanning the selected 18 codons of the *rpoB* gene, the CV of the T_m.S_ ranged from 0.03 to 0.24%. Similarly, CV values obtained for the assay’s INH resistance detection part ranged from 0.03 to 0.4% based on the T_m.S_. Our data underscored a markedly low variability in the T_m.S_ detected by the assay. Inter-assay reproducibility was examined by replicating the intra-assay experiment across three different days to further assess the variability inherent to the multiplex PCR-MPMA assay. The inter-assay CV for the T_m.S_ was observed to range between 0.02 and 0.53%.

**Table 2:** Results of precision evaluation of the multiplex PCR-MPMA assay for the detection of *rpoB*, *katG*, *ahpC* and *inhA* mutations

Drug	Target gene	Detected mutation	Template DNA concentration (copies/ml)	Number of replication	Intra-assay			Inter-assay		
					Mean T_m_ of 10 replicates (℃)	SD (℃)	CV (%)	Mean T_m_ of three runs (℃)	SD (℃)	CV (%)
RIF	*rpoB*	511CTG/CCG	5 × 10^6^	10	68.4	0.12	0.18	69.8	0.30	0.44
			5 × 10^4^		69.1	0.11	0.16	70.1	0.08	0.11
		513CAA/CCA	5 × 10^6^		62.3	0.08	0.13	63.8	0.14	0.22
			5 × 10^4^		62.5	0.10	0.16	62.7	0.10	0.16
		513CAA/AAA	5 × 10^6^		58.6	0.14	0.24	58.4	0.27	0.46
			5 × 10^4^		58.2	0.06	0.10	58.9	0.20	0.34
		513CAA/CTA	5 × 10^6^		61.4	0.09	0.15	61.0	0.18	0.30
			5 × 10^4^		61.7	0.07	0.11	62.3	0.11	0.18
		516GAC/GTC	5 × 10^6^		69.2	0.10	0.14	68.2	0.16	0.23
			5 × 10^4^		70.0	0.11	0.16	68.8	0.23	0.33
		516GAC/TAC	5 × 10^6^		68.3	0.04	0.06	69.5	0.11	0.16
			5 × 10^4^		68.4	0.08	0.12	70.2	0.25	0.36
		516GAC/GGC	5 × 10^6^		68.1	0.11	0.16	68.3	0.36	0.53
			5 × 10^4^		68.8	0.10	0.15	68.5	0.14	0.20
		517–518CAGAAC/–	5 × 10^6^		69.8	0.13	0.19	69.4	0.13	0.19
			5 × 10^4^		69.1	0.11	0.16	69.3	0.05	0.07
		531TCG/TTG	5 × 10^6^		72.0	0.06	0.08	72.1	0.14	0.19
			5 × 10^4^		72.3	0.08	0.11	71.8	0.09	0.13
		531TCG/TGG	5 × 10^6^		69.8	0.14	0.20	70.5	0.24	0.34
			5 × 10^4^		70.6	0.02	0.03	70.7	0.14	0.20
		531TCG/TTT	5 × 10^6^		68.3	0.08	0.12	68.0	0.22	0.32
			5 × 10^4^		68.5	0.10	0.15	68.8	0.02	0.03
		533CTG/CCG	5 × 10^6^		76.0	0.16	0.21	76.1	0.06	0.08
			5 × 10^4^		75.6	0.04	0.05	76.3	0.13	0.17
		522TCG/TTG	5 × 10^6^		74.1	0.12	0.16	74.3	0.07	0.09
			5 × 10^4^		74.4	0.05	0.07	74.4	0.05	0.07
		526CAC/GAC	5 × 10^6^		74.6	0.15	0.20	74.2	0.13	0.18
			5 × 10^4^		74.8	0.05	0.07	74.5	0.09	0.12
		526CAC/TAC	5 × 10^6^		74.3	0.05	0.07	74.8	0.12	0.16
			5 × 10^4^		74.7	0.13	0.17	74.6	0.15	0.20
		526CAC/CTC	5 × 10^6^		77.2	0.06	0.08	77.5	0.19	0.25
			5 × 10^4^		77.9	0.17	0.22	77.8	0.23	0.30
		526CAC/CGC	5 × 10^6^		77.2	0.10	0.13	77.7	0.30	0.39
			5 × 10^4^		77.6	0.08	0.10	77.2	0.24	0.31
		510–512CAGCTGAGC/–	5 × 10^6^		62.2	0.05	0.08	62.0	0.04	0.06
			5 × 10^4^		62.0	0.04	0.06	62.1	0.25	0.40
INH	*katG*	315AGC/ACC	5 × 10^6^		69.5	0.12	0.17	69.0	0.14	0.20
			5 × 10^4^		69.6	0.11	0.16	69.3	0.34	0.49
		315AGC/AAC	5 × 10^6^		70.2	0.23	0.33	70.4	0.05	0.07
			5 × 10^4^		70.8	0.22	0.31	70.2	0.01	0.01
		316GGC/AGC	5 × 10^6^		70.2	0.11	0.16	70.4	0.14	0.20
			5 × 10^4^		70.6	0.14	0.20	70.3	0.07	0.10
		316GGC/GAC	5 × 10^6^		70.2	0.16	0.23	70.2	0.03	0.04
			5 × 10^4^		70.3	0.15	0.21	70.0	0.14	0.20
	*ahpC*	2CCA/TCA	5 × 10^6^		62.4	0.16	0.26	62.2	0.22	0.35
			5 × 10^4^		62.0	0.18	0.29	62.4	0.15	0.24
		3CTG/AAG	5 × 10^6^		58.8	0.16	0.27	58.5	0.13	0.22
			5 × 10^4^		58.4	0.21	0.36	58.7	0.04	0.07
		5ACC/ATC	5 × 10^6^		62.4	0.17	0.27	62.3	0.18	0.29
			5 × 10^4^		62.0	0.18	0.29	62.3	0.06	0.10
		– 46G/A	5 × 10^6^		62.4	0.25	0.40	62.3	0.21	0.34
			5 × 10^4^		62.6	0.14	0.22	62.7	0.36	0.57
		– 44T/A	5 × 10^6^		63.2	0.11	0.17	63.3	0.21	0.33
			5 × 10^4^		63.5	0.05	0.08	63.8	0.07	0.11
		– 40T/C	5 × 10^6^		64.5	0.16	0.25	64.3	0.16	0.25
			5 × 10^4^		64.6	0.24	0.37	64.1	0.24	0.37
		– 39C/T	5 × 10^6^		60.2	0.04	0.07	60.7	0.18	0.30
			5 × 10^4^		60.8	0.07	0.12	60.2	0.06	0.10
		– 34T/C	5 × 10^6^		62.7	0.23	0.37	63.0	0.08	0.13
			5 × 10^4^		62.4	0.15	0.24	62.9	0.12	0.19
		– 34T/A	5 × 10^6^		63.5	0.18	0.28	63.7	0.11	0.17
			5 × 10^4^		63.8	0.10	0.16	63.4	0.15	0.24
		– 32G/A	5 × 10^6^		63.1	0.21	0.33	63.4	0.06	0.09
			5 × 10^4^		62.9	0.13	0.21	63.2	0.31	0.49
		– 30C/T	5 × 10^6^		60.5	0.07	0.12	60.7	0.14	0.23
			5 × 10^4^		60.0	0.19	0.32	60.9	0.02	0.03
		– 15C/T	5 × 10^6^		68.4	0.06	0.09	67.9	0.04	0.06
			5 × 10^4^		68.1	0.08	0.12	68.0	0.31	0.46
		– 12C/T	5 × 10^6^		65.3	0.16	0.25	65.5	0.05	0.08
			5 × 10^4^		65.1	0.10	0.15	65.8	0.21	0.32
		– 10C/T	5 × 10^6^		65.2	0.02	0.03	65.2	0.18	0.28
			5 × 10^4^		65.4	0.17	0.26	65.1	0.14	0.22
		– 9G/A	5 × 10^6^		67.1	0.13	0.19	67.4	0.24	0.36
			5 × 10^4^		67.7	0.12	0.18	67.9	0.22	0.32
		– 6G/A	5 × 10^6^		67.5	0.07	0.10	67.8	0.15	0.22
			5 × 10^4^		67.6	0.09	0.13	67.6	0.04	0.06
	*inhA*	– 15C/T	5 × 10^6^		61.3	0.16	0.26	61.2	0.13	0.21
			5 × 10^4^		61.5	0.13	0.21	61.3	0.25	0.41
		– 8T/A	5 × 10^6^		64.8	0.08	0.12	64.5	0.01	0.02
			5 × 10^4^		64.2	0.04	0.06	64.3	0.04	0.06

### Evaluation of specificity and analytical sensitivity of the multiplex PCR-MPMA assay

**Fig. 3:**
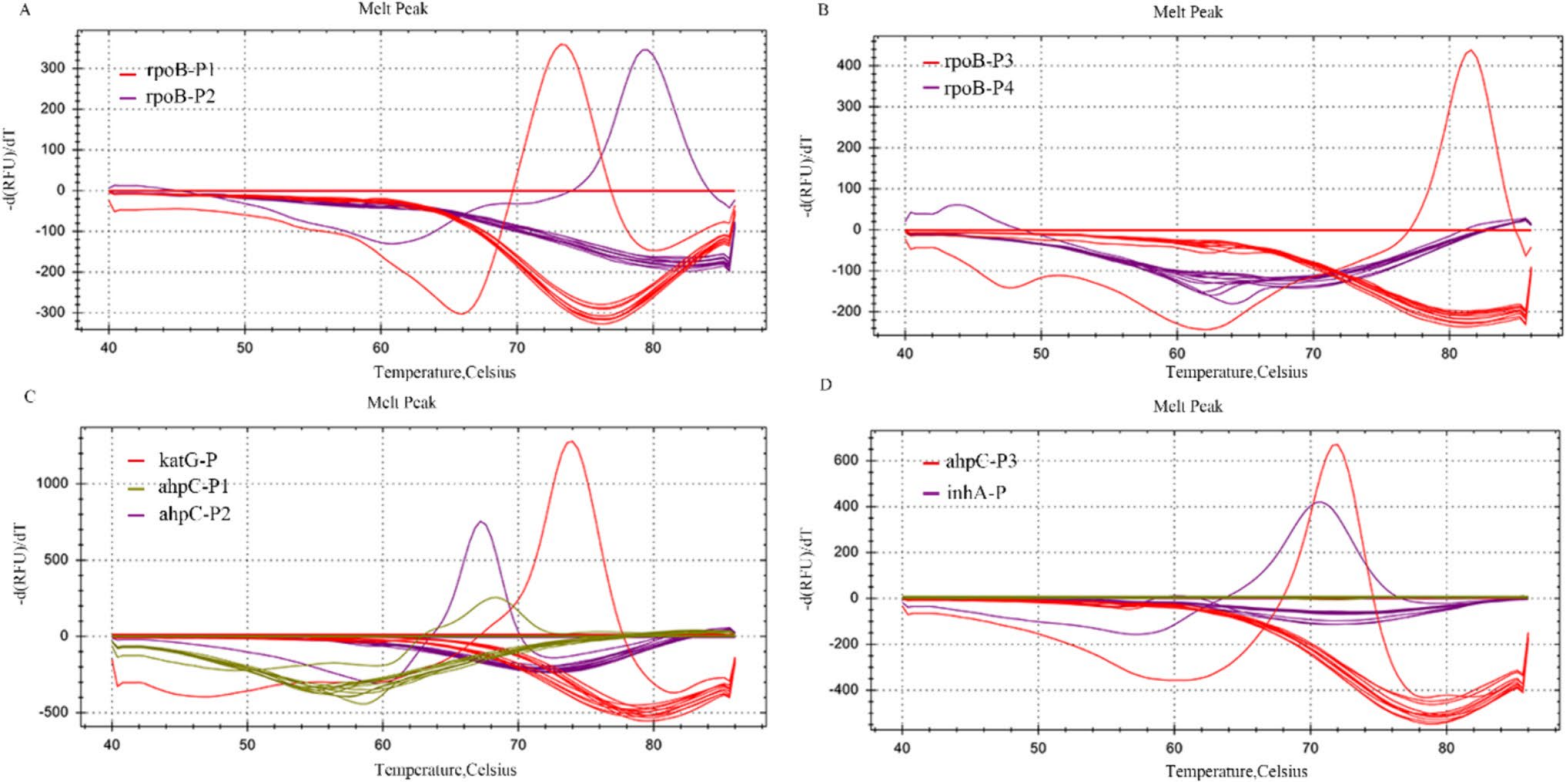
Evaluation of specificity of multiplex PCR-MPMA assay. Melting curves produced during tests of WT *M. tuberculosis* plasmids are indicated in each tube (**A**, **B**) *rpoB* (**C**) *katG* and *ahpC* (**D**) *ahpC* and *inhA,* suggesting the presence of RIF- and INH- susceptible *M. tuberculosis.* And no melting curves were generated from the nine RIF and INH resistance detection probes when non-target pathogen nucleic acids were used, indicating that background levels of nontuberculous mycobacteria and respiratory pathogens do not interfere with the analytical specificity of our developed assay for drug resistance detection

A homology analysis by BLAST confirmed that the primers and probes used in the multiplex PCR-MPMA assay for detecting RIF and INH resistance exhibited no homology to genomic sequences of other bacilli, thereby ensuring assay specificity. This specificity was further validated by the simultaneous detection of 5 × 10^6^ copies/ml genome equivalent DNA extracted from a panel of non-specific control microorganisms, as described in the relevant Methods section, alongside a positive control (nucleic acids extracted from reference strain *M. tuberculosis* H37Rv cells and measured at the identical concentration of 5 × 10^6^ copies/ml) and a no template control (NTC) in the same tubes. As shown in Fig. 3, the melting curves obtained from all four tubes exclusively showed the WT patterns, indicating that none of the non-specific control microorganisms were detected, aside from the target *M. tuberculosis,* thereby affirming the assay’s high specificity.

**Fig. 4:**
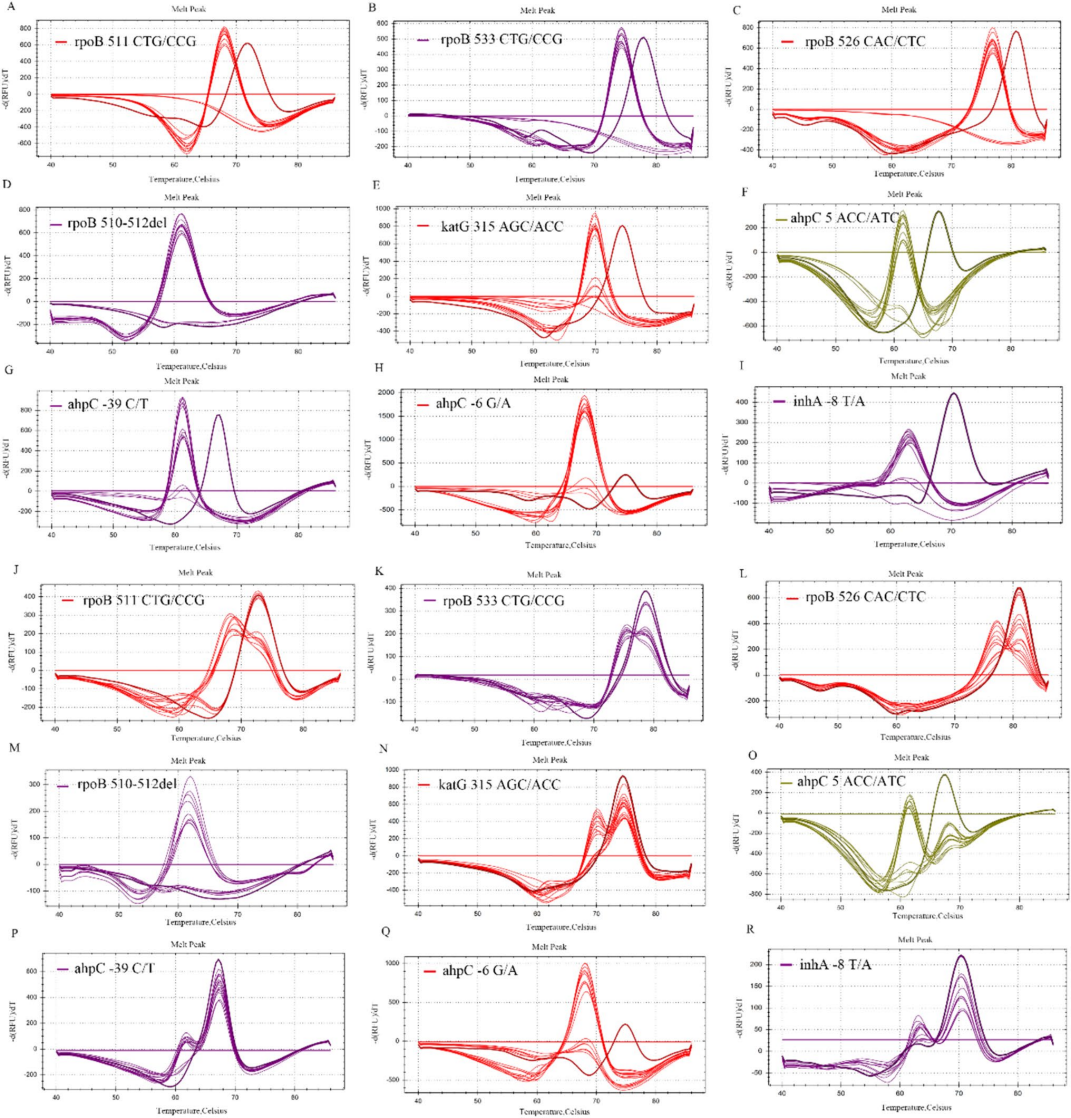
Detection of the dynamic range and hetero-resistance of the multiplex PCR-MPMA assay. Validation of the assay dynamic range for the detection of *M. tuberculosis* RIF (**A**–**D**) and INH (**E**–**I**) was performed with serial tenfold dilutions of the mixtures of WT and mutant reference standard plasmids (WT plasmid: mutant plasmid = 2: 3; concentrations from 5 × 10^6^ to 5 × 10^2^ copies/ml). The presence of RIF- or INH-resistant mutant plasmids was consistently detected in samples containing DNA templates at concentrations as high as 5 × 10^6^ copies/ml to as low as 5 × 10^3^ copies/ml. Determination of the assay capacity for discrimination of hetero-resistance of *M. tuberculosis* RIF (**J**, **K**, **L**, **M**) and INH (**N**, **O**, **P**, **Q**, **R**) by testing samples containing different proportions of each WT and each mutant plasmid as shown in the individual graph. This assay resulted in a predominant mutant peak in all mixtures containing as much as 60% mutant plasmids and a distinct double peak in a proportion of mixtures containing as little as 40% mutant plasmids. This assay failed intermittently to detect RIF and INH resistance in mixtures either at a concentration of 500 copies/ml or with only 30% mutant plasmids

To determine the assay’s LOD for RIF- and INH- resistant mutations, serial tenfold dilutions of mixtures comprising WT and mutant reference standard plasmids (WT plasmid:mutant plasmid = 2:3; concentrations from 5 × 10^6^ to 5 × 10^2^ copies/ml) were conducted as DNA templates, with each concentration measured in triplicate. The LOD for the positive control plasmids carrying the target genes was established at 5000 copies/ml, equivalent to 125 copies per 25 µl reaction volume, upon which both the WT and mutant types exhibited smooth and distinct melting curves morphologies (Fig. 4, A–I). Consequently, no qualified curves were generated at the lowest tested concentration of 500 copies/ml. This detection limit of 5000 copies/ml was further confirmed by repeating the measurement 10 times, with all four tubes yielding distinct and smooth melting curves under this template DNA concentration condition (Supplemental Figure S2), achieving a 100% detection rate.

Given the imperative to identify both drug-susceptible and -resistant strains in clinically diagnosed TB patients, the analytical capacity of our assay was validated against templates of a DNA mixture series containing varying ratios of mutant versus WT. The mutant plasmids containing the mutations: L511P, L533P, H526Y and 510–512 deletion in the *rpoB* gene, S315T in the *katG* gene, C(-39)T, G(-6)A and T5L in the *ahpC* gene, and T(-8)A in the *inhA* gene, mixed with background WT plasmids at variable percentages at a fixed total concentration as templates, were measured by the assay. The results, showcasing distinct double peaks responsive in the mutant melting curves, indicated a 40% fraction of mutant plasmids amidst 60% WT plasmids as the minimum detectable fraction to distinguish RIF and INH-associated hetero-resistance (Fig. 4, J–R). This implies that the assay’s detection capacity is adept at identifying mutation levels surpassing 40% in hetero-resistant samples.

### Evaluation of the assay’s diagnostic performance using clinical specimens

**Fig. 5:**
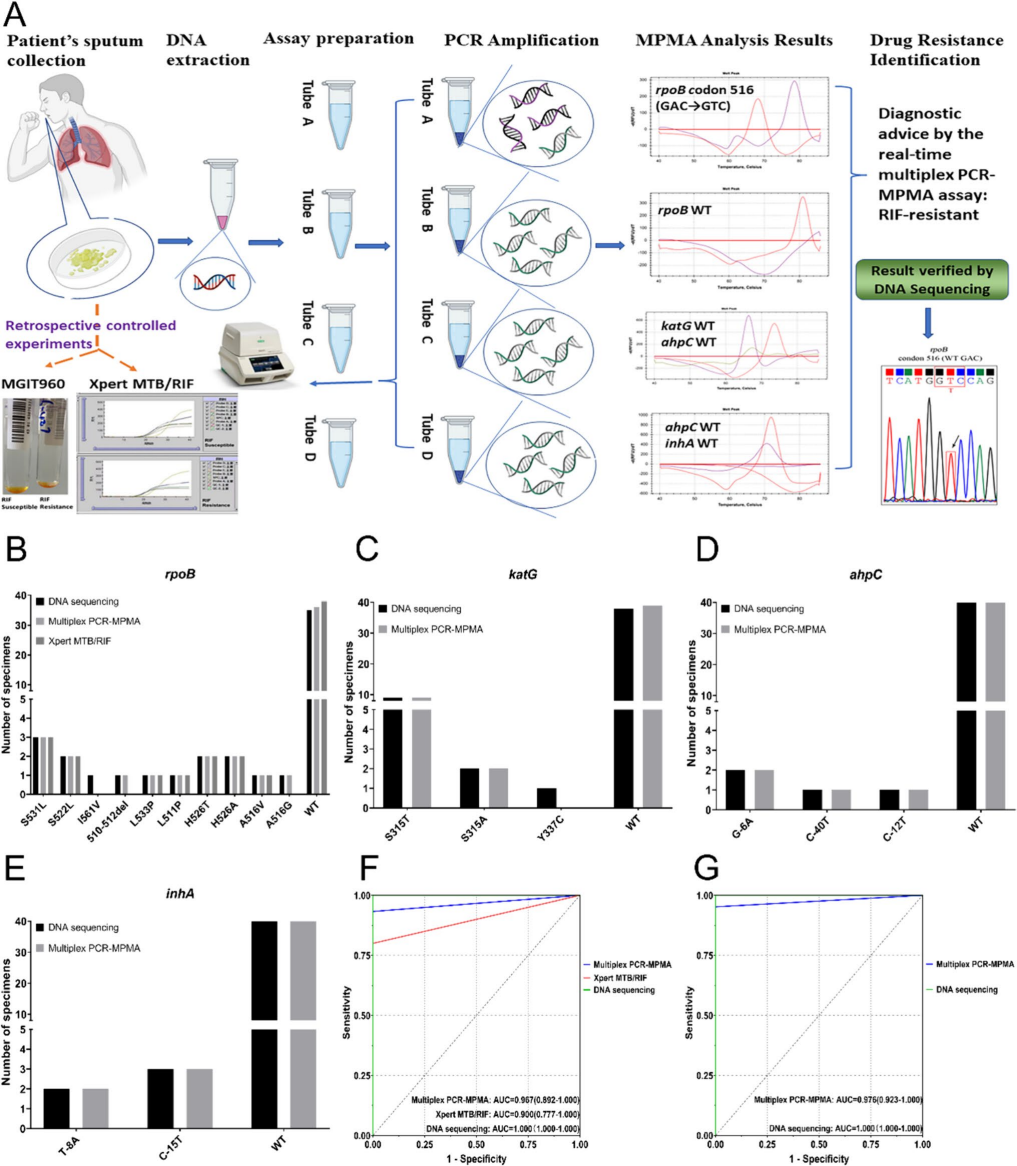
Diagnostic performance evaluation of the multiplex PCR-MPMA assay. **A** Schematic representation of diagnostic process of the multiplex PCR-MPMA assay. The process is graphically illustrated in the following cartoon, which individually depicts the collection of sputum specimens from TB patients, the simultaneous amplification of the extracted nucleic acids in the four tubes of the multiplex PCR, the analysis of the probe-produced melting curves versus those of the WT controls, the diagnostic confirmation of the genotypic results of the multiplex PCR-MPMA assay by DNA sequencing, and further validation of the results through retrospective analysis, including *M. tuberculosis* culture, phenotypic DST at the gold standard level and head-to-head comparison with WHO-recommended Cepheid’s Xpert MTB/RIF. If the difference in melting curve T_m_ values between the tested sample and the WT plasmid used as the positive control is greater than 2 °C (∆T_m_ ≥ 2 °C), a specific mutation can be identified indicating resistance to a particular drug. For example, if in tube A, one of the T_m_ values of the melting curves generated by the probes targeting the *rpoB* gene matches the WT value and the other is very close to the value of the mutant D516V (within ± 1 °C), combined with the shapes of the melting curves, while the melting curves generated by the other three tubes agree with the WT T_m_ values, it can be initially diagnosed that the sample belongs to a patient resistant to RIF but susceptible to INH. DNA sequencing serves as the reference standard for confirming the genotypic results. B-E, categorization of analyzed specimens in the mutant/WT category. The data in this figure are categorized according to the genes to much the mutant loci belong, regardless of mono-resistance to RIF or INH or MDR to both RIF and INH. **B** 8 mutated loci in the *rpoB* gene and 15 specimens that harbored a total of 11 mutations at each locus were determined by DNA sequencing. I561V was incorrectly denoted as WT by the multiplex PCR-MPMA assay. In addition to I561V, A516G and del 510–512 in the *rpoB* gene were reported as susceptible to RIF by the Xpert MTB/RIF assay **C** 2 mutated loci in *katG* gene and 13 samples that carried 3 mutations at each locus were determined by sequencing. Y337C was incorrectly identified as WT by the multiplex PCR-MPMA assay. **D** 4 samples that harbored *ahpC* G-6A, C-40T and C-12T mutations were confirmed by sequencing. 22 samples were designated as WT by both multiplex PCR-MPMA assay and DNA sequencing. **E** 5 *inhA* T-8A and C-15T mutant samples were determined by sequencing and no discrepant results were produced between the two methods. **F**, **G** receiver operating characteristic (ROC) curves for multiplex PCR-MPMA, Xpert MTB/RIF and DNA sequencing assays. **F** The diagnostic performance of multiplex PCR-MPMA, Xpert MTB/RIF and DNA sequencing for detecting RIF resistance were assessed as the area under the ROC curve (AUC). The AUC values for multiplex PCR-MPMA, Xpert MTB/RIF and DNA sequencing were 0.967, 0.900 and 1.000, indicating comparable and highly accurate diagnostic capabilities. **G** The assessment focused on the diagnostic performance of multiplex PCR-MPMA and DNA sequencing in detecting INH resistance, using AUC as the indicator. The AUC values for multiplex PCR-MPMA and DNA sequencing were 0.976 and 1.000, indicating similarly high diagnostic accuracies

Prior to molecular diagnostic procedures, all sputum specimens from the 50 patients with definitive pulmonary TB underwent direct smear microscopy examination employing Ziehl Neelsen’s staining for AFB, as described in the Methods, verifying the presence of AFB with a bacillary load of at least 1+. The bacterial infection status was further substantiated by the findings of the retrospective evaluation study. Each of the 50 clinical sputum samples was analyzed using this multiplex fluorescence PCR-MPMA assay, with the drug susceptibility results compared to the DNA sequencing data of the targeted loci obtained concurrently, as well as the results of the phenotypic DST and Xpert MTB/RIF (Table 3). Fundamentally, all methods facilitated effective detection across the 50 samples, yielding results of the requisite analytical quality, whilst the DNA sequencing results were in complete agreement with those of the phenotypic DST, the gold standard. Secondly, this multiplex PCR-MPMA assay accurately identified 8 sputum samples resistant to both RIF and INH, aligning with the DNA sequencing and phenotypic DST findings, revealing the *rpoB* mutation in combination with any of the *katG*, *inhA* or *ahpC* mutations detected in the corresponding samples. Thirdly, the *rpoB* portion of the multiplex PCR-MPMA assay accurately detected 6 out of 7 RIF mono-resistant samples, while the *katG*/*inhA*/*ahpC* portion of the assay correctly identified 12 out of 13 INH mono-resistant specimens, when compared to the phenotypic DST. More specifically, these 2 discrepant samples were designated as WT by the assay, one harboring a I561V mutation in the *rpoB* region at codon 561 and the other possessing a Y337C mutant genotype in the *katG* region at codon 337 by DNA sequencing, with neither mutation within the detection range of the probes designed for this assay. This is corroborated by the fact that while Xpert MTB/RIF can only detect mutations associated with RIF resistance, it reported three false-negative cases, all of which were outside the range of the probes developed by the manufacturer. This intimates that DNA sequencing identified additional mutant genotypes in the clinical samples used for assay validation. From these mono-resistant samples reported by the assay, melting curves, in addition to the observed mutations, matched either *rpoB* or *katG*/*inhA*/*ahpC* gene WT plasmid controls in the other tubes of the four-tube system, indicating they were identified as resistant to one drug but susceptible to the other. Supplemental Figure S3 shows representative electropherograms of DNA sequencing for detected mutants associated with RIF- or INH-resistance. Figure 5A illustrates the workflow of the multiplex PCR-MPMA assay for the detection of clinical sputum specimens, verified by DNA sequencing and retrospectively validated by bacterial culture, phenotypic DST, and Xpert MTB/RIF.

**Table 3:** Multiplex PCR-MPMA assay screening of 50 clinical sputum specimens for RIF and INH resistance compared with the results from DNA sequencing, MGIT960 liquid culture and Cepheid’s Xpert MTB/RIF

Sputum specimen ID	DNA sequencing result			Multiplex PCR-MPMA result	MGIT960 liquid culture result		Xpert MTB/RIF result	
	Locus	Nucleotide change	Amino acid change		*M. tuberculosis*	Drug resistance	MTB	RIF
1	*rpoB* 531	TCG → TTG	Ser → Leu	RIF-resistant/INH-susceptible	+	RIF	+	+
2	*katG* 315	AGC → ACC	Ser → Thr	INH-resistant/RIF-susceptible	+	INH	+	–
3	/	WT (*rpoB*, *katG*, *inhA* and *ahpC*)	/	Susceptible	+	–	+	–
4	*katG* 315	AGC → ACC	Ser → Thr	INH-resistant/RIF-susceptible	+	INH	+	–
5	*rpoB* 516	GAC → GTC	Asp → Val	RIF-resistant/INH-susceptible	+	RIF	+	+
6	*rpoB* 516, *katG* 315	GAC → GGC, AGC → AAC	Asp → Gly, Ser → Asn	RIF- and INH-resistant	+	RIF/INH	+	–
7	*rpoB* 526, *ahpC* -12	CAC → GAC, C → T	His → Asp, /	RIF- and INH-resistant	+	RIF/INH	+	+
8	*katG* 315	AGC → ACC	Ser → Thr	INH-resistant/RIF-susceptible	+	INH	+	–
9	*rpoB* 533	CTG → CCG	Leu → Pro	RIF-resistant/INH-susceptible	+	RIF	+	+
10	*rpoB* 526, *katG* 315	CAC → TAC, AGC → ACC	His → Tyr, Ser → Thr	RIF- and INH-resistant	+	RIF/INH	+	+
11	*ahpC* -40	C → T	/	INH-resistant/RIF-susceptible	+	INH	+	–
12	*ahpC* -6	G → A	/	INH-resistant/RIF-susceptible	+	INH	+	–
13	*rpoB* 522, *inhA* -15	TCG → TTG, C → T	Ser → Leu, /	RIF- and INH-resistant	+	RIF/INH	+	+
14	*rpoB* 511, *inhA* -8	CTG → CCG, T → A	Leu → Pro, /	RIF- and INH-resistant	+	RIF/INH	+	+
15	/	WT (*rpoB*, *katG*, *inhA* and *ahpC*)	/	Susceptible	+	–	+	–
16	/	WT (*rpoB*, *katG*, *inhA* and *ahpC*)	/	Susceptible	+	–	+	–
17	/	WT (*rpoB*, *katG*, *inhA* and *ahpC*)	/	Susceptible	+	–	+	–
18	/	WT (*rpoB*, *katG*, *inhA* and *ahpC*)	/	Susceptible	+	–	+	–
19	*katG* 315	AGC → ACC	Ser → Thr	INH-resistant/RIF-susceptible	+	INH	+	–
20	/	WT (*rpoB*, *katG*, *inhA* and *ahpC*)	/	Susceptible	+	–	+	–
21	*rpoB* 522	TCG → TTG	Ser → Leu	RIF-resistant/INH-susceptible	+	RIF	+	+
22	/	WT (*rpoB*, *katG*, *inhA* and *ahpC*)	/	Susceptible	+	–	+	–
23	*inhA* -15	C → T	/	INH-resistant/RIF-susceptible	+	INH	+	–
24	*rpoB* 526, *katG* 315	CAC → CGC, AGC → ACC	His → Arg, Ser → Thr	RIF- and INH-resistant	+	RIF/INH	+	+
25	*rpoB* 531	TCG → TTG	Ser → Leu	RIF-resistant/INH-susceptible	+	RIF	+	+
26	*inhA* -15	C → T	/	INH-resistant/RIF-susceptible	+	INH	+	–
27	*inhA* -8	T → A	/	INH-resistant/RIF-susceptible	+	INH	+	–
28	*rpoB* 510–512	Deletion	/	RIF-resistant/INH-susceptible	+	RIF	+	–
29	/	WT (*rpoB*, *katG*, *inhA* and *ahpC*)	/	Susceptible	+	–	+	–
30	/	WT (*rpoB*, *katG*, *inhA* and *ahpC*)	/	Susceptible	+	–	+	–
31	/	WT (*rpoB*, *katG*, *inhA* and *ahpC*)	/	Susceptible	+	–	+	–
32	/	WT (*rpoB*, *katG*, *inhA* and *ahpC*)	/	Susceptible	+	–	+	–
33	/	WT (*rpoB*, *katG*, *inhA* and *ahpC*)	/	Susceptible	+	–	+	–
34	*rpoB* 526, *ahpC* -6	CAC → TAC, G → A	His → Tyr, /	RIF- and INH-resistant	+	RIF/INH	+	+
35	/	WT (*rpoB*, *katG*, *inhA* and *ahpC*)	/	Susceptible	+	–	+	–
36	/	WT (*rpoB*, *katG*, *inhA* and *ahpC*)	/	Susceptible	+	–	+	–
37	/	WT (*rpoB*, *katG*, *inhA* and *ahpC*)	/	Susceptible	+	–	+	–
38	*rpoB* 561	ATC → GTC	Ile → Val	Susceptible	+	RIF	+	–
39	*katG* 315	AGC → ACC	Ser → Thr	INH-resistant/RIF-susceptible	+	INH	+	–
40	*katG* 337	TAC → TGC	Tyr → Cys	Susceptible	+	INH	+	–
41	/	WT (*rpoB*, *katG*, *inhA* and *ahpC*)	/	Susceptible	+	–	+	–
42	/	WT (*rpoB*, *katG*, *inhA* and *ahpC*)	/	Susceptible	+	–	+	–
43	/	WT (*rpoB*, *katG*, *inhA* and *ahpC*)	/	Susceptible	+	–	+	–
44	/	WT (*rpoB*, *katG*, *inhA* and *ahpC*)	/	Susceptible	+	–	+	–
45	*katG* 315	AGC → AAC	Ser → Asn	INH-resistant/RIF-susceptible	+	INH	+	–
46	*rpoB* 531, *katG* 315	TCG → TTG, AGC → ACC	Ser → Leu, Ser → Thr	RIF- and INH-resistant	+	RIF/INH	+	+
47	*katG* 315	AGC → ACC	Ser → Thr	INH-resistant/RIF-susceptible	+	INH	+	–
48	/	WT (*rpoB*, *katG*, *inhA* and *ahpC*)	/	Susceptible	+	–	+	–
49	/	WT (*rpoB*, *katG*, *inhA* and *ahpC*)	/	Susceptible	+	–	+	–
50	/	WT (*rpoB*, *katG*, *inhA* and *ahpC*)	/	Susceptible	+	–	+	–

DNA sequencing analysis revealed the following RIF and/or INH resistance profiles. Specifically, the 7 samples harboring single genotypic mutations involving the *rpoB* gene included 2 having S531L mutation, 1 exhibiting 510–512 deletion, and each of the remaining 4 showcasing mutation in codon 516, 522, 533, and 561, respectively. The 13 samples classified as INH mono-resistance encompassed 6 samples displaying a *katG* S315T mutant, 2 samples showing a *katG* S315N and a *katG* Y337C mutation, 2 samples carrying mutations located in the *ahpC* promoter region (position − 40, − 6) and 3 samples harboring mutations in the *inhA* promoter region (two at the − 15 position and one at the − 8 site). The 8 samples categorized as both RIF and INH resistance demonstrated a more complex mix of mutant genotypes as these samples carried mutations in both *rpoB* (codon 511, 516, 522, 526 and 531) as well as *katG* (codon 315), or *ahpC* region (position − 12 and − 6), or *inhA* region (position − 8 and − 15). Figure 5B–E categorize the specimens based on their mutant/WT status as determined by either PCR-MPMA, Xpert MTB/RIF (data available for detection of mutation sites in the *rpoB* gene), or DNA sequencing.

Table 4 summarizes the results of PCR-MPMA, DNA sequencing and Xpert MTB/RIF screening of the 50 clinical sputum specimens compared to the culture-based phenotypic DST for RIF and INH resistance. Notably, our assay demonstrated 100% specificity, identifying samples as either RIF-resistant or INH-resistant, thereby no WT sample was erroneously reported as a mutant. The two discrepant samples resulted in an assay overall sensitivity of 93.33% (95% CI 65.76–100.00%) for RIF resistance detection and 95.24% (95% CI 74.10–100.00%) for INH resistance identification, respectively. Furthermore, these two samples led to the NPV of 97.22% (95% CI 83.90–100.00%) for RIF resistance testing and 96.67% (95% CI 81.03–100.00%) for INH resistance testing, while the PPV for both drugs attained 100%. In contrast, DNA sequencing achieved 100% sensitivity and specificity when compared with the gold standard. However, the three discrepant samples reporting RIF susceptibility resulted in a sensitivity of 80.00% (95% CI 51.40–95.97%) for Xpert MTB/RIF in RIF resistance detection in this study. The coincidence between the multiplex PCR-MPMA assay and phenotypic DST for RIF and INH resistance detection was calculated to be 98.00% (95% CI 88.10–100.00%), compared to 100.00% (95% CI 91.06–100.00%) for DNA sequencing and 94.00% (95% CI 82.71–98.78%) for Xpert MTB/RIF, indicating that the three molecular-based assays have comparable accuracies in comparison to phenotypic DST results. The McNemar’s test (also known as the paired Chi-square) was employed to analyze the statistical significance of the discordance in detection accuracy between the gold standard phenotypic DST and each of the three molecular assays. The calculated *p*-value of > 0.999 for the multiplex PCR-MPMA and DNA sequencing in all aspects of RIF and INH resistance detection, and a 0.248 *p*-value for Xpert MTB/RIF in RIF resistance detection did not yet reach statistical significance, indicating no significant difference in the performance among all three tests. Moreover, the effectiveness of multiplex PCR-MPMA, DNA sequencing and Xpert MTB/RIF in RIF or INH resistance detection was further evidenced by plotting the sensitivity and specificity of each test (sensitivity against 1-specificity) to calculate the AUC values, as shown in Fig. 5F and G. In terms of diagnosing RIF resistance, the AUC values were 0.967, 0.900 and 1.000 for multiplex PCR-MPMA, Xpert MTB/RIF and DNA sequencing, respectively (Fig. 5F). Similarly, for assessing INH resistance, the AUC values were 0.976 and 1.000 for multiplex PCR-MPMA and DNA sequencing, respectively (Fig. 5G). These findings demonstrate the exceptional diagnostic accuracy of all three molecular approaches in identifying either RIF or INH resistance.

**Table 4:** Diagnostic performance of multiplex PCR-MPMA assay, DNA sequencing and Xpert MTB/RIF assay for detecting gene mutations associated with RIF and INH resistance

Assay		Drug	MGIT960 liquid culture		Total	Sensitivity^a^ [% (95% CI)]	Specificity^a^ [% (95% CI)]	PPV^a^ [% (95% CI)]	NPV^a^ [% (95% CI)]	Coincidence^a^ [% (95% CI)]	McNemar’s test^a^ (*p* value)
			Resistance	Susceptible							
Multiplex PCR-MPMA	RIF	Resistance	14	0	14	93.33 (65.76–100.00)	100.00 (87.56–100.00)	100.00 (72.43–100.00)	97.22 (83.90–100.00)	98.00 (88.10–100.00)	> 0.999
		Susceptible	1	35	36						
		Total	15	35	50						
	INH	Resistance	20	0	20	95.24 (74.10–100.00)	100.00 (85.26–100.00)	100.00 (79.55–100.00)	96.67 (81.03–100.00)	98.00 (88.1–100.00)	> 0.999
		Susceptible	1	29	30						
		Total	21	29	50						
DNA Sequencing	RIF	Resistance	15	0	15	100.00 (80.40–100.00)	100.00 (85.26–100.00)	100.00 (80.40–100.00)	100.00 (85.26–100.00)	100.00 (91.06–100.00)	> 0.999
		Susceptible	0	35	35						
		Total	15	35	50						
	INH	Resistance	21	0	21	100.00 (80.40–100.00)	100.00 (85.26–100.00)	100.00 (80.40–100.00)	100.00 (85.26–100.00)	100.00 (91.06–100.00)	> 0.999
		Susceptible	0	29	29						
		Total	21	29	50						
Xpert MTB/RIF	RIF	Resistance	12	0	12	80.00 (51.40–95.97)	100.00 (87.56–100.00)	100.00 (68.79–100.00)	92.11 (77.80–98.39)	94.00 (82.71–98.78)	0.248
		Susceptible	3	35	38						
		Total	15	35	50						

^a^The calculation method for each indicator listed in this table is presented as follows. TP = true positive; FP = false positive; TN = true negative; FN = false negative. Sensitivity = TP/(TP + FN); Specificity = TN/(TN + FP); PPV (positive predictive value) = TP/(TP + FP); NPV (negative predictive value) = TN/(TN + FN); Coincidence = (TP + TN)/(TP + FP + TN + FN). The reference standard used to assess individual assay’s diagnostic performance was mycobacterial culture and phenotypic drug susceptibility testing (DST). A McNemar’s test (paired Chi-square test) was performed on the paired dichotomous resistance-susceptible data and a p-value of < 0.05 indicates a significant difference in detection performance between the two methods. The 95% CI was calculated using the Wilson score confidence interval formula

## Discussion

Molecular diagnostics are increasingly recognized as a crucial complement to traditional phenotypic antibiotic susceptibility testing to address the time-consuming limitations inherent to bacterial culture. In China, the burden of TB prevention and control predominantly lies on numerous county-level secondary hospitals which often operate under resource constraints but lacking adequate facilities for *M. tuberculosis* culture and gold standard DST. Our collaborating Chaoshan Hospital of Jinan University First Affiliated Hospital represents such hospitals facing constraints associated with biosafety-compliant bacterial culture laboratories and costly MGIT™ 960 liquid culture/DST system. However, amidst the endeavor to curb local outbreaks of the severe acute respiratory syndrome coronavirus 2 (SARS-CoV-2) virus and the resulting coronavirus disease 2019 (COVID-19) pandemic, fluorescence PCR instruments have gained significant popularity in China’s primary care [35]. This serendipitously positions Chaoshan Hospital, equipped with multi-channel fluorescence PCR instruments, as a conducive setting for deploying our multiplex PCR-MPMA assay to fully exploit the potential of fluorescence real-time PCR technology for rapid molecular diagnostics, enabling frontline healthcare facilities to perform follow-up testing to sputum smear microscopy. Additionally, this assay, by monitoring drug resistance in patients receiving specific medications, facilitates timely adjustments of therapeutic regimens, aligning with the objective of the WHO 2021TPP.

*M. tuberculosis* acquires drug resistance through specific genetic alterations, including point mutations, deletions or insertions [14]. The WHO recommends routine testing of all TB patients for resistance to RIF and INH, the classic first-line drugs [29]. Extensive studies have illumined the genetic mechanisms of resistance. RIF resistance is predominantly attributed to mutations in the 81-bp fragment RRDR of the *rpoB* gene, which accounts for about 95% of cases [36]. However, it is worth noting that certain studies have reported rare mutations outside this region, such as I491F, I59T, V146F and I572F, which potentially confer RIF resistance but are not currently targeted by commercially available assays [37, 38]. Specifically, I572F and six other located within the RRDR-namely, L511P, D516Y, H526L, H526N, H526C, and L533P-are often considered “disputed” or “occult”, which can mask MDR-TB when phenotypically assessed by methods such as the MGIT™ 960, and can lead to potential treatment failure or relapse if not detected early [38]. In contrast, INH resistance unravels through a complex genetic narrative, with previous studies reporting over 80% of INH-resistant *M. tuberculosis* strains harbouring mutations at codon 315 of *katG*, alongside positions -15 and -8 of the *inhA* promoter and positions -6 to -47 of the *ahpC* promoter [39–41]. Noteworthily, while some previous reports suggest that the *ahpC* gene serves only to compensate for the loss of peroxidase function caused by *katG* mutations in many INH-resistant isolates, two meta-analyses have jointly reported that mutations in 11 loci within a 106-bp intergenic region between the pseudogene *oxyR* and the *ahpC* gene are associated with INH resistance [42, 43]. One of the two studies also observed a global prevalence of 1.84%, with regional prevalence reaching 2.71% in Asia, a phenomenon transcending the mere compensatory mechanism of *katG* mutations [43]. Despite the generally acknowledged clinical relevance of the *fabG1* gene for INH resistance detection, it was not specially investigated in this study. The decision was steered by a recent study employing whole-genome sequencing to analyze mutations associated with INH resistance in 188 MDR-TB and single INH-resistant clinical isolates collected in a Chinese national survey. Remarkably, no mutations in the *fabG1* gene were detected, whereas the same study reported high mutation frequencies in the *katG* (86.2%), *inhA* promoter (19.6%), and *oxyR-ahpC* intergenic regions (18.6%) [19]. As described in the Methods section, our strategy for selecting variants to detect, including *rpoB* RRDR coding and deletion mutations, *katG* coding mutations, *ahpC* coding and promoter mutations, and *inhA* promoter mutations, was informed by the WHO guideline, the listed commercial assays and the literature review. Furthermore, in recent years, very few PCR-HRMA studies demonstrating a mutation-resistance association have directly tested sputum samples from TB patients, with one exception, but that study also did not include the *inhA* gene [44]. Consequently, this study aims to bridge this gap by evaluating diagnostic performance using clinical sputum samples, thereby providing valuable insights for researchers in this field.

Our multiplex PCR-MPMA assay utilizes an inventive combination of fluorescence and melting temperature labelling to detect the selected mutant sequences in a high-throughput, multicolour melting curve analysis framework. Consequently, by meticulously scrutinizing a unique melting peak plus a T_m_ value in each mutation as identifiable markers for the discrimination of 4 WT from a total number of 40 mutant sequences, our assay possess the diagnostic potential to analyse the genetic information of resistance to RIF and INH. Careful optimization improved assay’s performance, including optimized nucleic acid extraction to obtain high-quality template DNA, intensive Molecular Beacon probe design to increase the specificity of melting analysis, and fragment length adjustment to avoid primer dimerization. A salient feature of our assay is its ability to discriminate between insertion and deletion mutations, whereas we observed that base substitution mutations can lead to very diverse differentiation effects, depending on the difference in T_m_ values between the mutant and WT targets. For example, mutation types G/A, G/T, C/A and C/T were readily distinguished (T_m_ difference > 0.5 °C), while G/C (T_m_ difference < 0.4 °C) and A/T (T_m_ difference < 0.2 °C) were much more difficult to distinguish. To address this problem, our application of Molecular Beacon probes significantly bolsters the specificity of experiments. This was demonstrated that the shorter double-stranded portion formed by the binding of the probes to their complementary regions, combined with the Touch-down PCR reaction cycling program, worked better to identify selected mutant sequences. In addition, the application of asymmetric PCR as a replacement for standard PCR was crucial because, after the completion of asymmetric PCR, the probes hybridized to a large number of specific sites on the single strands with increasing temperature. As a result, this combination enabled at least 2.4 °C T_m_ shifts between mutant and WT samples, as shown by single-plex PCR evaluation data (Supplemental Table S5). These visible differences allowed us to effectively discriminate between drug-susceptible and drug-resistant clinical specimens for the diagnosis of TB patients. However, this sensitivity leads to false positives from synonymous mutations. Our data from the detection of plasmids containing the five synonymous mutations, Q510Q, F514F, Q513Q, D516D and L533L reported RIF resistance. Despite the remarkable sensitivity of the probe being advantageous in identifying minor variations, the detection of synonymous mutations may not necessarily be pertinent for cases pertaining to RIF resistance in certain clinical scenarios. This fundamental drawback is inherent in the melting curve assay methodology. In this regard, our assay did not prove superior to Cepheid’s Xpert MTB/RIF, which uses a similar methodology that is nested PCR combined with melting curve analysis, in the detection of synonymous mutations to avoid false positives [25].

The multiplex PCR-MPMA assay was evaluated for its analytical performance, validating 40 different mutations within *rpoB* (18 codon mutations), *katG* (4 codon mutations), *ahpC* (3 codon mutations and 13 promotor region mutations) and *inhA* (2 promotor region mutations) using the relevant commercially synthesized plasmids (Supplemental Table S2). The ddPCR technique was used for precise quantification of the nucleic acid molecules in the template plasmids, aiding in determining the assay’s analytical sensitivity. All 40 mutations plus the corresponding WT from each gene were simultaneously correctly detected by the four-tube, multiplex PCR-MPMA assay. Unlike other fluorescence PCR assays that use amplification curves and Ct values as detection limit indicators, the LOD of our assay was determined using a proportional mixture of WT and mutant artificial plasmids as templates to test for qualitative detection of characteristic melting peaks and T_m_ values representative of WT and mutant samples under serial dilution. The assay LOD was defined as the lowest concentration at which characteristic melting peaks/ T_m_ values were reliably detectable, based on achieving 100% success rate in 10 replicates. Therefore, our assay was determined to achieve a LOD of 5000 copies/ml, equivalent to 125 copies/reaction, but it must be clarified that these outcomes pertain solely to the input DNA samples. Given the increasing prevalence of infection with mixed susceptibility and resistance to a drug in the MDR population as evidenced by recent studies [45, 46], an alternative LOD validation experiment was performed to investigate the extent to which our assay could unequivocally identify hetero-resistant mutants mixed with WT drug-sensitive strains. Our data corroborated the assay’s analytical rigor, with its capacity to distinguish as little as 40% of the desired hetero-resistant mutants mixed with WT being particularly noteworthy. This suggests that our assay’s performance for detecting hetero-resistance is consistent with a study using laboratory-modified Xpert MTB/RIF assay to achieve comparable results [47]. Intra- and inter-assay CVs below 0.5% demonstrated robust and reproducible T_m_ values, thereby reinforcing the consistency of our assay’s performance and affirming its stability and precision.

The latter phase of this study transitioned from assay development to a critical evaluation of its diagnostic performance, involved in collaboration with clinical patients. We designed a protocol to consecutively recruit a compact cohort of confirmed TB patients, facilitating a comparative analysis of our assay against DNA sequencing data obtained during the same period and retrospectively, against reference standards like liquid culture, phenotypic DST and the WHO recommended Xpert MTB/RIF assay. The authors candidly admit that, at the inception of this study, challenges in establishing a collaboration with a centralized diagnostic laboratory precluded the synchronous implementation of all designated control tests during the clinical evaluation phase of our assay. Initially, we intended to partner with a specialist hospital and Chaoshan Hospital to conduct all tests concurrently, thereby validating our assay’s efficacy in the intended application scenario. However, unforeseen circumstances led to a year’s delay in performing the gold standard tests and comparator test post clinical sample collection, necessitating a shift to retrospective analyses. This timing misalignment forced us to conduct the assay’s field test suboptimally. A significant limitation is that solely detecting resistance mutations from smear- positive clinical sputum samples doesn’t provide a comprehensive assessment of our assay, as data from smear- negative samples are essential for a balanced evaluation. Understandably, enrolling suspected TB patients without bacterial confirmation of *M. tuberculosis* infection during the participant screening phase would introduce bias. Additionally, the inability to utilize advanced diagnostic equipment not available in county-level hospitals, such as bronchofiberscopes, results in another notable limitation of this study-specifically, the lack of comprehensive evaluation of diagnostic performance across other types of respiratory specimens, including bronchoalveolar lavage fluid.

Nevertheless, after the retrospective analysis, this study aligns the minimal criteria for the evaluation of innovative products for molecular TB-DR detection products defined by the WHO 2021TPP at the level of experimental protocol design and implementation, encompassing the requisite reference standards and comparative tests. Given the potential of this technique for TB diagnosis, the selection of the *rpoB* gene as the target within the *Mycobacterium* genomic region is a noteworthy step. However, extensive clinical trials are requisite to validate the application of our assay as a primary screening test for *M. tuberculosis*. A pertinent illustration of a comparable diagnostic approach is Cepheid’s Xpert MTB/RIF. Much like our focus on the *rpoB* gene, the Xpert MTB/RIF, a WHO-approved molecular rapid diagnostic (mWRD) test, facilitates the initial diagnosis of *M. tuberculosis* rather than serving as a reflex test to a TB-positive result [22, 24, 36]. The inclusion of DNA sequencing in this study was founded on the premise of phenotypic DST being the gold standard reference used in retrospective validation. Selecting DNA sequencing as a reference tool to furnish genotypic information for the multiplex PCR-MPMA assay outcomes was buttressed by a WHO report. This document elucidated that the accuracy of sequencing in predicting INH, RIF, FQ and SLI resistance compared to phenotypic DST results, coupled with its sufficient sensitivity in appraising the prevalence of drug resistance in TB surveillance, substantiates its choice as a reference tool [48].

The focus of this study was to develop a novel multiplex PCR-MPMA assay for ascertaining drug resistance status, for which we conducted verification experiments under defined scenarios mirroring the assay’s intended application. The empirical data obtained from these experiments supplied substantial evidence of the assay’s efficacy when deployed on samples from the target patient population. Our findings underscored a sensitivity of 93.33% and 95.24% for detecting RIF and INH resistance, respectively, with a specificity of 100% against phenotypic DST. These findings were consistent with the previously published study validating their PCR-HRMA assay via sputum samples, which reported HRMA sensitivity of 90.5%, 86.4% and 100% for pinpointing mutations in the *rpoB*, *katG* and *inhA* genes, respectively [44]. In our assay, we observed a notable discrepancy: one missing sample detection each for the *rpoB* and *katG* probes. This was primarily due to the presence of mutations in the patient samples that fell outside the predefined mutation detection range of our designed probes, leading to reduced diagnostic sensitivity. In comparison, the retrospective analysis of the Xpert MTB/RIF assay revealed three false-negative samples with a sensitivity of 80%. These false-negative results were attributed to the inability of the manufacturer’s probes to capture the specific mutation sites carried by these patients. The AUC values, delineated in Fig. 5 F, evinced a comparable accuracy among our assay, Xpert MTB/RIF and DNA sequencing in detecting RIF resistance within the examined cohort, with respective AUC values of 0.967, 0.900 and 1.000. Similarly, in detecting INH resistance, our assay and DNA sequencing exhibited a tight diagnostic concordance, as illustrated in Fig. 5G, with AUC values of 0.976 and 1.000, respectively. The diagnostic performance of our assay, juxtaposed against both the WHO-approved reference test and the Xpert MTB/RIF assay, showcased comparable efficacy. Overall, despite the fact that the phenotypic DST results used as reference standards were obtained from retrospective analyses, the measured sensitivities of 93.33% for RIF resistance and 95.24% for INH resistance stand in parallel with the pooled sensitivities of 94% for RIF and 93% for INH elucidated in a 2021 systematic review and meta-analysis encompassing 47 HRMA-based studies [49]. It’s necessary to emphasize that, unlike the meta-analysis which primarily analyzed data from clinical isolates of *M. tuberculosis* derived from 47 studies, our investigation uniquely utilized sputum samples, enhancing the clinical feasibility of our findings.

Our multiplex PCR-MPMA assay identified resistant samples, a finding corroborated by DNA sequencing. These samples harboured a total of 17 mutated loci, including well-documented mutation hotspots such as codons 531, 526 and 516 within the RRDR of the *rpoB* gene responsible for RIF resistance, alongside locus 315 of *katG*, locus -15 of *inhA* and locus -8 of *inhA*, the three most frequently mutated loci related to INH resistance. Moreover, our assay detected the disputed mutations at codons 511 and 533, specifically L511P and L533P, in two samples, providing compelling evidence of our assay’s capability to identify mutations that contribute to low-level DR. DNA sequencing additionally identified two uncommon loci, locus 561 of *rpoB* and locus 337 of *katG*. These mutations have been consistently reported in numerous studies employing commercial testing protocols or laboratory-developed assays [21, 44, 47, 50, 51]. Among the 50 samples analyzed, DNA sequencing authenticated 11 mutations across 15 samples associated with RIF resistance, revealing no significant variance in their distribution. Although the high-frequency mutation S531L in *rpoB* (TCG → TTG) manifested in merely 3 samples, three mutations at another commonly reported locus, *rpoB526* (CAC → GAC, CAC → TAC and CAC → CGC), were found across 4 samples cumulatively. Similarly, this clinical validation confirmed that our assay, in alignment with DNA sequencing, accurately identified the mutation S315T (AGC → ACC) in the *katG* gene in 9 (18%) samples, thereby corroborating with literature that this mutation type responsible for INH resistance is predominant. Interestingly, the mutations *rpoB* I561V (ATC → GTC) and *katG* Y337C (TAC → TGC), overlooked by our assay yet recognized by DNA sequencing, have been delineated in merely two preceding publications, hence are deemed rare [52, 53]. In addition, regarding *rpoB* mutation detection, our experiments using the Xpert MTB/RIF assay failed to detect samples carrying *rpoB* A516G (GAC → GGC) and *rpoB* del 510–512, alongside *rpoB* I561V, likely due to these mutations transcending the probe target range as per Cepheid’s disclosed information. Should ensuing studies persist in discovering these two rare mutations in larger cohorts, their sequences could be integrated into newly developed probes within an upgraded assay. Overall, 28 out of 50 TB patients (56%) were found to harbor mono-resistance to RIF (7, 14%) or INH (13, 26%), or MDR (8, 16%) to both drugs, as ascertained by our assay coupled with DNA sequencing. This substantial proportion of drug resistance, encapsulating MDR-TB, as demonstrated by the findings of this study, represents a fundamental challenge confronting global TB control, highlighting its marked significance in public health and direct clinical relevance.

Similar to other molecular tests, our assay is specifically designed to detect certain resistant mutations, inherently introducing a risk of misdiagnosis due to its inability to cover all potential mutations. For instance, our study demonstrated sensitivities of 93.33% for RIF and 95.24% for INH, slightly lower than those reported in a prior study, which claimed a 100% HRMA sensitivity in smear-positive sputum samples [44]. The discrepancy can be attributed to the non-detection of a single mono-resistant sample for each drug, a limitation stemming from our probe design. Nonetheless, the inherent flexibility of our assay facilitates swift adaptation to newly reported mutations. By incorporating or modifying probes based on prevalent types discovered through pathogenic and epidemiological studies, we can effectively reduce the misdiagnosis risk. Addressing our decision against selecting the *fabG1* gene as a detection target, our probe design strategy has appropriately considered this factor. It is noteworthy that mutations in the *inhA* promoter, including those upstream of *fabG1,* overlap with the *inhA* gene and serve as promoters for the entire *inhA-fabG1* operon [43]. Our assay targets specific sites like C-15T and T-8C within the promoter region, which are well-documented and globally recognised as drivers of INH resistance [43]. Additionally, as mentioned earlier, *rpoB* mutations outside the 81-bp RRDR, including I491F, I59T, V146F and I572F, are infrequent and remained undetected by our current probes. We remain committed to monitoring emerging literature to determine if adding probes for these mutations enhances RR-TB detection sensitivity. Pertaining to the potential false positives associated with the identification of synonymous mutations in the RRDR within the *rpoB* gene, we are planning rigorous clinical validations to assess the incidence of such mutations. Upon validation through sequencing, we can refine our probes and modulate fluorescent moiety to classify these mutations as WT, effectively minimizing false positive impact.

The updated WHO 2021TPP outlines both minimum and optimal criteria for technical and operational specifications of next generation TB DST protocols, guiding the development of innovative molecular diagnostics intended for use in primary healthcare centres [9, 10]. Our multiplex PCR-MPMA assay aligns with the 2021TPP’s four technical specifications: target selection, analytical performance, operational and infrastructural requirements, and pricing. This ensures an affordable, efficient TB DR screening tool tailored for peripheral laboratories with specific needs. We retrospectively conducted phenotypic DST on liquid culture, using it as the gold standard, and compared our assay’s performance with Cepheid’s Xpert MTB/RIF during the clinical validation phase. This comprehensive evaluation contextualized our assay within the 2021TPP framework. Furthermore, we benchmarked technical specifications of our assay against the six commercially available assays, utilizing WHO published documents as our data resource [22–24, 36] (detailed in Supplementary Material Figure S4). Our assay excels in drug and mutation site coverage and offers competitive diagnostic performance (Table S1). For RIF resistance diagnosis, our 93.33% combined sensitivity is slightly lower than Cepheid’s Xpert MTB/RIF (94.4%), BD’s Max MDR-TB (99.1%), Bruker/Hain’s FluoroType MTBDR (97%), and Abbott’s RealTime MTB RIF/INH (94%), but outperforms Roche’s cobas MTB-RIF/INH (91%) and Molbio’s Truenat MTB-RIF Dx (84%). Moreover, our 95.24% combined sensitivity for detecting INH resistance is consistent with competitors.

Uniquely, unlike our assay necessitates minimal infrastructural investment compared to the six proprietary commercial systems. It is fully compatible with various renowned real-time fluorescence PCR instruments, thereby eliminating the necessity to modify existing molecular pathogen detection protocols, making its compatibility beneficial in diverse laboratory environments. Our assay workflow comprises three key steps: sputum liquefaction, decontamination and centrifugal concentration, nucleic acid extraction, and PCR amplification and melting curve analysis, aligning with the 2021TPP’s five-step maximum. All six competitors also require sputum liquefaction, albeit with varying incubation times. Notably, protocols from Cepheid, Roche, and BD offer integrated nucleic acid extraction and testing, while others, including ours, necessitate a separate nucleic acid extraction and PCR amplification detection process. Thus, despite its significantly lower initial infrastructure costs, our assay is marginally inferior in terms of biosafety compared to the aforementioned fully integrated systems, owing to the necessity for manual sample transfers between distinct procedural stages. This manual handling, despite using closed tube operations to reduce exposure to infectious agents, introduces potential biosafety challenges that are inherently mitigated in more seamless, integrated assay configurations. Our assay’s time efficiency stands out: 40 min for sputum specimen pre-processing, 40 min for nucleic extraction, 10 min for PCR manipulation, and 2 h 40 min for PCR amplification and melting curve analysis. Consequently, our assay can analyse up to 22 patient samples (including negative and positive quality control references) within approximately 4.2 h using a standard 96-well fluorescence PCR instrument. In contrast, Cepheid’s Xpert MTB/RIF requires 1.5 h for *M. tuberculosis* and RIF resistance detection, followed by an additional 1.5 h for INH resistance detection. Molbio’s protocol entails 20 min for DNA extraction, 1 h for *M. tuberculosis* detection and 1 h for RIF resistance detection. It is worth noting that the total protocol time for the other four platforms to complete both drug resistance detections exceeds that of our assay. Considering the expenses associated with fluorescent probes, molecular diagnostic enzymes, and other necessary reagents for assay development, we have set an ex-factory price at $10 per test. In contrast, Cepheid offers negotiated mass availability rates of $9.90 for Xpert MTB/RIF and $19.80 for Xpert MTB/XDR, implying an ex-factory cost of $29.70 for the required RIF and INH resistance tests. The authors acknowledge that the fee of $29.70 levied by Cepheid, represents a costlier yet comprehensive test for suspected MDR-TB cases, covering not only the detection of resistance to RIF and INH but also extending to FQ, second-line injectable drugs (such as amikacin, kanamycin, and capreomycin), and ethionamide [24], thereby justifying the value of Xpert MTB/XDR in terms of the extensive DR profiling it offers. Negotiations between the other companies and the WHO have yet to determine a final ex-factory price for low- and middle- income countries. Therefore, our assay demonstrates compatibility and aligns with the guiding principles outlined in the 2021TPP, promising to enhance public health in peripheral centres.

Incorporating matched sequencing data in our study was essential for identifying and interpreting mutations linked to resistance phenotypes which were retrospectively verified by bacteriological culture and phenotypic DST. To ensure a thorough evaluation of our assay, it is imperative to use bacteriological evidence and DST as reference standards. Future studies should draw from a diverse patient population, factoring in various screening and inclusion criteria, such as age, gender, socio-geographical characteristics, clinical symptoms, diagnosis/treatment history, and culture outcome. This holistic approach facilitates robust analytical modelling. It is also advisable for subsequent research to categorize specimens, encompassing sputum smear-negative specimens, clinical culture isolates and bronchoalveolar lavage fluid. Analyzing varied sample types offers a richer diagnostic performance evaluation. Once thoroughly evaluated, our assay promises several advantages: solid analytical results, extensive coverage, and the flexibility to update mutation targets, positioning it highly effectively for first-line *M. tuberculosis* drug resistance screening and epidemiological studies. Finally, the rigorous validation and comprehensive reporting conducted for our assay adheres to the key criteria outlined in the Standards for Reporting Diagnostic Accuracy (STARD) 2015 guidelines [54], including appropriate use of reference standards and transparent discussion of limitations and further investigation, ensuring a trustworthy and accurate evaluation of this novel diagnostic tool.

## Conclusion

In summary, we have successfully developed a multiplex PCR-MPMA assay that exhibits strong diagnostic capabilities using AFB*-*positive sputum specimens from pulmonary TB patients. This assay simultaneously and accurately detects 40 mutations associated with resistance to RIF and INH, striking a balance between compatibility, simplicity and cost effectiveness. Its potential applications span routine research, TB epidemiology diagnostics, and clinical care, while also facilitating genetic characterization of *M. tuberculosis* in a more advanced and simplified manner.

## Supplementary Information

Below is the link to the electronic supplementary material.

Supplementary file1 (DOCX 2467 KB)

## Data Availability

The manuscript and its supporting information files encompass all pertinent data. Raw experimental data, including export files of PCR reactions, and derived data supporting the findings of this study can be available from the corresponding author X. Jiang upon reasonable request.
