# Supplementary material for: Accurate and affordable detection of rifampicin and isoniazid resistance in Tuberculosis sputum specimens by multiplex PCR-multiple probes melting analysis
Source: Infection. 2024 Jun 17;52(6):2371–98. doi: 10.1007/s15010-024-02295-w (PMC11621165; doi:10.1007/s15010-024-02295-w)
Supplement: Supplementary file 1 — Supplementary file1 (DOCX 2467 KB) [file 15010_2024_2295_MOESM1_ESM.docx]

**Supplementary information**

**Additional file 1**

**Table S1:** **List of genomic regions⃰ associated with RIF or INH resistance detected by the six WHO-recommended commercial assays.** The mutation sites targeted by each commercial assay that overlap with our assay are highlighted.

| Manufacturer | Product | Methodology | Detected Resistance | Targeted Gene | Detected Mutation | Notes |
| --- | --- | --- | --- | --- | --- | --- |
| Cepheid | Xpert MTB/RIF  Xpert MTB/RIF Ultra | Real Time PCR  with Melting Curve Analysis | RIF | *rpoB* | Gln510Val |  |
|  |  |  |  |  | Leu511Pro |  |
|  |  |  |  |  | Gln513Lys |  |
|  |  |  |  |  | del 516 |  |
|  |  |  |  |  | Asp516Val |  |
|  |  |  |  |  | del 518 |  |
|  |  |  |  |  | Ser522Leu |  |
|  |  |  |  |  | His526Cys |  |
|  |  |  |  |  | His526Asp |  |
|  |  |  |  |  | His526Leu |  |
|  |  |  |  |  | His526Asn |  |
|  |  |  |  |  | His526Arg |  |
|  |  |  |  |  | His526Tyr |  |
|  |  |  |  |  | stop 526 |  |
|  |  |  |  |  | Arg529Lys |  |
|  |  |  |  |  | Ser531Leu |  |
|  |  |  |  |  | Ser531Gln |  |
|  |  |  |  |  | Ser531Trp |  |
|  |  |  |  |  | Leu533Pro |  |
|  |  |  |  |  | Leu533Arg |  |
|  |  |  |  |  | His526Ser |  |
|  |  |  |  |  | Leu530Met Ser531Pro |  |
|  | Xpert MTB/XDR |  | INH | *inhA* | -1 to -32 | Promoter |
|  |  |  |  | *katG* | Ser315Thr |  |
|  |  |  |  | *fabG1* | Gly609Ala |  |
|  |  |  |  | *oxyR- ahpC* | -5 to -50 intergenic (or -47 to -92) | Intergenic region |
| Roche | Cobas MTB-RIF/INH | Real Time PCR | RIF | *rpoB* | Leu511Pro |  |
|  |  |  |  |  | Gln513Lys |  |
|  |  |  |  |  | Gln513Leu |  |
|  |  |  |  |  | Gln513Pro |  |
|  |  |  |  |  | Asp516Val |  |
|  |  |  |  |  | Asp516Tyr |  |
|  |  |  |  |  | Ser522Leu |  |
|  |  |  |  |  | Ser522Gln |  |
|  |  |  |  |  | His526Asp |  |
|  |  |  |  |  | His526Leu |  |
|  |  |  |  |  | His526Asn |  |
|  |  |  |  |  | His526Arg |  |
|  |  |  |  |  | His526Tyr |  |
|  |  |  |  |  | Ser531Leu |  |
|  |  |  |  |  | Ser531Trp |  |
|  |  |  |  |  | Leu533Pro |  |
|  |  |  |  |  | Ser522Trp |  |
|  |  |  |  |  | Asp516Gly |  |
|  |  |  | INH | *katG* | Ser315lle |  |
|  |  |  |  |  | Ser315Asn |  |
|  |  |  |  |  | Ser315Thr1 |  |
|  |  |  |  |  | Ser315Thr2 |  |
|  |  |  |  | *fabG1-inhA* | T-8A  T-8C  C-15T | Promoter |
| Abbott | RealTime MTB RIF/INH | Real Time PCR | RIF | *rpoB* | Ser531Leu |  |
|  |  |  |  |  | His526Tyr |  |
|  |  |  |  |  | Asp516Val |  |
|  |  |  |  |  | His526Asp |  |
|  |  |  |  |  | Leu533Pro |  |
|  |  |  |  |  | Ser531Trp |  |
|  |  |  |  |  | His526Leu |  |
|  |  |  |  |  | Gln513Pro |  |
|  |  |  |  |  | Asp516Phe |  |
|  |  |  |  |  | Ser531Phe |  |
|  |  |  |  |  | His526Gly |  |
|  |  |  |  |  | Gln513-514 insertion |  |
|  |  |  | INH | *katG* | Ser315Thr |  |
|  |  |  |  |  | Ser315Asn |  |
|  |  |  |  | *fabG1-inhA* | C-15T | Promoter |
| Becton-Dickinson | BD MAX MDR-TB | Real Time PCR | RIF | *rpoB* | Asp516Val |  |
|  |  |  |  |  | Ser531Leu |  |
|  |  |  |  |  | His526Tyr |  |
|  |  |  |  |  | His526Asp |  |
|  |  |  |  |  | Leu533Pro |  |
|  |  |  |  |  | His526Arg |  |
|  |  |  |  |  | Asp516Gly |  |
|  |  |  |  |  | Ser531Trp |  |
|  |  |  |  |  | His526Leu |  |
|  |  |  |  |  | Gln513Pro |  |
|  |  |  |  |  | Ser522Leu |  |
|  |  |  |  |  | Asp516Phe |  |
|  |  |  |  |  | Ser531Phe |  |
|  |  |  |  |  | His526Gly |  |
|  |  |  |  |  | Gln513-514 insertion |  |
|  |  |  | INH | *katG* | Ser315Thr |  |
|  |  |  |  | *fabG1-inhA* | C-15T | Promoter |
| Molbio Diagnostics | Truenat MTB-RIF Dx | Chip-based Real Time PCR | RIF | *rpoB* | Ser531Leu |  |
|  |  |  |  |  | His526Tyr |  |
|  |  |  |  |  | Asp516Val |  |
|  |  |  |  |  | His526Asp |  |
|  |  |  |  |  | His526Arg |  |
|  |  |  |  |  | Asp516Gly |  |
|  |  |  |  |  | Ser531Trp |  |
|  |  |  |  |  | His526Leu |  |
|  |  |  |  |  | Gln513Lys |  |
|  |  |  |  |  | Gln513Pro |  |
|  |  |  |  |  | Gln513Leu |  |
|  |  |  |  |  | Leu533Pro |  |
|  |  |  |  |  | Asp516Tyr |  |
|  |  |  |  |  | Ser522Leu |  |
|  |  |  |  |  | His526Asn |  |
| Hain Lifescience/Bruker | FluoroType MTBDR | Real Time PCR | RIF | *rpoB* | Ser531Leu |  |
|  |  |  |  |  | His526Tyr |  |
|  |  |  |  |  | Asp516Val |  |
|  |  |  |  |  | His526Asp |  |
|  |  |  |  |  | Leu533Pro |  |
|  |  |  |  |  | His526Arg |  |
|  |  |  |  |  | Asp516Gly |  |
|  |  |  |  |  | Ser531Trp |  |
|  |  |  |  |  | His526Leu |  |
|  |  |  |  |  | Gln513Pro |  |
|  |  |  |  |  | Ser522Leu |  |
|  |  |  |  |  | Asp516Phe |  |
|  |  |  |  |  | Ser531Phe |  |
|  |  |  |  |  | His526Gly |  |
|  |  |  |  |  | Gln513-514  insertion |  |
|  |  |  | INH | *katG* | Ser315Thr |  |
|  |  |  |  | *fabG1-inhA* | C-15T | Promoter |

Abbreviations: RIF, rifampicin; INH, isoniazid; WHO, World Health Organization.

⃰ The target genes and mutation loci corresponding to each commercial test solution listed in the table were obtained from the downloadable assay instructions published on the official websites of Cepheid, Roche, Abbott, Becton-Dickinson, Molbio Diagnostics and Hain Lifescience/Bruker.

**Table S2: List of plasmids containing WT sequences and the desired mutations in rpoB, katG, inhA and ahpC genes.** Negative positions are nucleotide positions relative to the start of the genes inhA and ahpC on the positive strand, while positive positions are codon numbers in the genes rpoB, katG and ahpC.

| Target gene | Mutation site analysed | Nucleotide change | Amino acid change | Plasmid number from Manufacturer | Plasmid  length(bp) | Supplier |
| --- | --- | --- | --- | --- | --- | --- |
| *rpoB* | Codon 511 | CTG/CCG | Leu→Pro | 211120PT8704-8R21 | 795 | Sangon, Inc (Shanghai, China) |
|  | Codon 513 | CAA/CCA | Gln→Pro | 211120HT8731-3 | 792 |  |
|  | Codon 513 | CAA/AAA | Gln→Lys | 211121HT9095-2R25 |  |  |
|  | Codon 513 | CAA/CTA | Gln→Leu | 211121HT9097-3 |  |  |
|  | Codon 516 | GAC/GTC | Asp→Val | 211121PT8978-8R22 | 777 |  |
|  | Codon 516 | GAC/TAC | Asp→Tyr | 211121PT8979-4R22 |  |  |
|  | Codon 516 | GAC/GGC | Asp→Gly | 211121PT8980-3R22 |  |  |
|  | Deletion of Codon 517-518 | CAGAAC | / | 211121HT9100-1 | 786 |  |
|  | Codon 531 | TCG/TTG | Ser→Leu | 211120PT8742-3R21 | 798 |  |
|  | Codon 531 | TCG/TGG | Ser→Trp | 211120PT8743-8R21 |  |  |
|  | Codon 531 | TCG/TTT | Ser→Phe | 210617PT2402-10R18 |  |  |
|  | Codon 533 | CTG/CCG | Leu→Pro | 211124HT0130-5 |  |  |
|  | Codon 522 | TCG/TTG | Ser→Leu | 211121HT9098-1 | 792 |  |
|  | Codon 526 | CAC/GAC | His→Asp | 211129PT1633-2R30 | 794 |  |
|  | Codon 526 | CAC/TAC | His→Tyr | 211120PT8745-4R21 |  |  |
|  | Codon 526 | CAC/CTC | His→Leu | 211120PT8746-4R21 |  |  |
|  | Codon 526 | CAC/CGC | His→Arg | 211125PT0383-3R26 |  |  |
|  | Deletion of Codon 510-512 | CAGCTGAGC | / | 211121HT9099-3 | 783 |  |
|  | Codon 510 | CAG/CAA | Gln→Gln | G0272039-1 | 568 |  |
|  | Codon 514 | TTC/TTT | Phe→Phe | G0272039-2 | 563 |  |
|  | Codon 516 | GAC/GAT | Asp→Asp | G0272039-3 |  |  |
|  | Codon 533 | CTG/TTG | Leu→Leu | G0272039-4 |  |  |
|  | Codon 513 | CAA/CAG | Gln→Gln | G0272039-5 |  |  |
|  | WT | / | / | 211121HT9101-4 | 792 |  |
| *katG* | Codon 315 | AGC/ACC | Ser→Thr | 211130HT1821-2 | 778 |  |
|  | Codon 315 | AGC/AAC | Ser→Asn | 211127HT1080-3 |  |  |
|  | Codon 316 | GGC/AGC | Gly→Ser | 211127HT1081-3 |  |  |
|  | Codon 316 | GGC/GAC | Gly→Asp | 211201HT2165-3 |  |  |
|  | WT | / | / | 211130HT1745-7 |  |  |
| *ahpC* | Codon 2 | CCA/TCA | Pro→Ser | 211121PT9006-2R22 | 838 |  |
|  | Codon 3 | CTG/AAG | Leu→Lys | 211121PT9007-2R22 |  |  |
|  | Codon 5 | ACC/ATC | Thr→Ile | 211121PT9008-11R22 |  |  |
|  | -46 | G/A | / | 211121HT9202-2R24 |  |  |
|  | -44 | T/A | / | 211121HT9190-1R24 |  |  |
|  | -40 | T/C | / | 211124HT0131-2R28 |  |  |
|  | -39 | C/T | / | 211121HT9192-5R24 |  |  |
|  | -34 | T/C | / | 211121HT9194-1R24 |  |  |
|  | -34 | T/A | / | 211129HT1588-7R2 |  |  |
|  | -32 | G/A | / | 211126HT0634-2R30 |  |  |
|  | -30 | C/T | / | 211121HT9196-2R24 |  |  |
|  | -15 | C/T | / | 211121HT9197-1R24 |  |  |
|  | -12 | C/T | / | 211121HT9198-1R24 |  |  |
|  | -10 | C/T | / | 211121HT9199-4R24 |  |  |
|  | -9 | G/A | / | 211121HT9200-3R24 |  |  |
|  | -6 | G/A | / | 211121HT9201-6 |  |  |
|  | WT | / | / | 211121HT9203-5R24 |  |  |
| *inhA* | -15 | C/T | / | 211126HT0881-5 | 794 |  |
|  | -8 | T/A | / | 211130HT1760-6 |  |  |
|  | WT | / | / | 211123HT9829-1 |  |  |

**Table S3: Complete sequences of the 49 plasmids used in assay development.** The nucleotide sequence of the desired mutation contained in each plasmid sequence and its location are highlighted.

| Plasmid ID | Plasmid Sequences |
| --- | --- |
| CJH4-531-TTG | GTCGCTATAAGGTCAACAAGAAGCTCGGGCTGCATGTCGGCGAGCCCATCACGTCGTCGACGCTGACCGAAGAAGACGTCGTGGCCACCATCGAATATCTGGTCCGCTTGCACGAGGGTCAGACCACGATGACCGTTCCGGGCGGCGTCGAGGTGCCGGTGGAAACCGACGACATCGACCACTTCGGCAACCGCCGCCTGCGTACGGTCGGCGAGCTGATCCAAAACCAGATCCGGGTCGGCATGTCGCGGATGGAGCGGGTGGTCCGGGAGCGGATGACCACCCAGGACGTGGAGGCGATCACACCGCAGACGTTGATCAACATCCGGCCGGTGGTCGCCGCGATCAAGGAGTTCTTCGGCACCAGCCAGCTGAGCCAATTCATGGACCAGAACAACCCGCTGTCGGGGTTGACCCACAAGCGCCGACTGTTGGCGCTGGGGCCCGGCGGTCTGTCACGTGAGCGTGCCGGGCTGGAGGTCCGCGACGTGCACCCGTCGCACTACGGCCGGATGTGCCCGATCGAAACCCCTGAGGGGCCCAACATCGGTCTGATCGGCTCGCTGTCGGTGTACGCGCGGGTCAACCCGTTCGGGTTCATCGAAACGCCGTACCGCAAGGTGGTCGACGGCGTGGTTAGCGACGAGATCGTGTACCTGACCGCCGACGAGGAGGACCGCCACGTGGTGGCACAGGCCAATTCGCCGATCGATGCGGACGGTCGCTTCGTCGAGCCGCGCGTGCTGGTCCGCCGCAAGGCGGGCGAGGTGGAGTACGTGCCCTCGTCTGAGGTGGACT |
| CJH5-531-TGG | GTCGCTATAAGGTCAACAAGAAGCTCGGGCTGCATGTCGGCGAGCCCATCACGTCGTCGACGCTGACCGAAGAAGACGTCGTGGCCACCATCGAATATCTGGTCCGCTTGCACGAGGGTCAGACCACGATGACCGTTCCGGGCGGCGTCGAGGTGCCGGTGGAAACCGACGACATCGACCACTTCGGCAACCGCCGCCTGCGTACGGTCGGCGAGCTGATCCAAAACCAGATCCGGGTCGGCATGTCGCGGATGGAGCGGGTGGTCCGGGAGCGGATGACCACCCAGGACGTGGAGGCGATCACACCGCAGACGTTGATCAACATCCGGCCGGTGGTCGCCGCGATCAAGGAGTTCTTCGGCACCAGCCAGCTGAGCCAATTCATGGACCAGAACAACCCGCTGTCGGGGTTGACCCACAAGCGCCGACTGTGGGCGCTGGGGCCCGGCGGTCTGTCACGTGAGCGTGCCGGGCTGGAGGTCCGCGACGTGCACCCGTCGCACTACGGCCGGATGTGCCCGATCGAAACCCCTGAGGGGCCCAACATCGGTCTGATCGGCTCGCTGTCGGTGTACGCGCGGGTCAACCCGTTCGGGTTCATCGAAACGCCGTACCGCAAGGTGGTCGACGGCGTGGTTAGCGACGAGATCGTGTACCTGACCGCCGACGAGGAGGACCGCCACGTGGTGGCACAGGCCAATTCGCCGATCGATGCGGACGGTCGCTTCGTCGAGCCGCGCGTGCTGGTCCGCCGCAAGGCGGGCGAGGTGGAGTACGTGCCCTCGTCTGAGGTGGACT |
| CJH6-526 -GAC | AGAAGCTCGGGCTGCATGTCGGCGAGCCCATCACGTCGTCGACGCTGACCGAAGAAGACGTCGTGGCCACCATCGAATATCTGGTCCGCTTGCACGAGGGTCAGACCACGATGACCGTTCCGGGCGGCGTCGAGGTGCCGGTGGAAACCGACGACATCGACCACTTCGGCAACCGCCGCCTGCGTACGGTCGGCGAGCTGATCCAAAACCAGATCCGGGTCGGCATGTCGCGGATGGAGCGGGTGGTCCGGGAGCGGATGACCACCCAGGACGTGGAGGCGATCACACCGCAGACGTTGATCAACATCCGGCCGGTGGTCGCCGCGATCAAGGAGTTCTTCGGCACCAGCCAGCTGAGCCAATTCATGGACCAGAACAACCCGCTGTCGGGGTTGACCGACAAGCGCCGACTGTCGGCGCTGGGGCCCGGCGGTCTGTCACGTGAGCGTGCCGGGCTGGAGGTCCGCGACGTGCACCCGTCGCACTACGGCCGGATGTGCCCGATCGAAACCCCTGAGGGGCCCAACATCGGTCTGATCGGCTCGCTGTCGGTGTACGCGCGGGTCAACCCGTTCGGGTTCATCGAAACGCCGTACCGCAAGGTGGTCGACGGCGTGGTTAGCGACGAGATCGTGTACCTGACCGCCGACGAGGAGGACCGCCACGTGGTGGCACAGGCCAATTCGCCGATCGATGCGGACGGTCGCTTCGTCGAGCCGCGCGTGCTGGTCCGCCGCAAGGCGGGCGAGGTGGAGTACGTGCCCTCGTCTGAGGTGGACTACATGGACGTCTCG |
| CJH7-526-TAC | AGAAGCTCGGGCTGCATGTCGGCGAGCCCATCACGTCGTCGACGCTGACCGAAGAAGACGTCGTGGCCACCATCGAATATCTGGTCCGCTTGCACGAGGGTCAGACCACGATGACCGTTCCGGGCGGCGTCGAGGTGCCGGTGGAAACCGACGACATCGACCACTTCGGCAACCGCCGCCTGCGTACGGTCGGCGAGCTGATCCAAAACCAGATCCGGGTCGGCATGTCGCGGATGGAGCGGGTGGTCCGGGAGCGGATGACCACCCAGGACGTGGAGGCGATCACACCGCAGACGTTGATCAACATCCGGCCGGTGGTCGCCGCGATCAAGGAGTTCTTCGGCACCAGCCAGCTGAGCCAATTCATGGACCAGAACAACCCGCTGTCGGGGTTGACCTACAAGCGCCGACTGTCGGCGCTGGGGCCCGGCGGTCTGTCACGTGAGCGTGCCGGGCTGGAGGTCCGCGACGTGCACCCGTCGCACTACGGCCGGATGTGCCCGATCGAAACCCCTGAGGGGCCCAACATCGGTCTGATCGGCTCGCTGTCGGTGTACGCGCGGGTCAACCCGTTCGGGTTCATCGAAACGCCGTACCGCAAGGTGGTCGACGGCGTGGTTAGCGACGAGATCGTGTACCTGACCGCCGACGAGGAGGACCGCCACGTGGTGGCACAGGCCAATTCGCCGATCGATGCGGACGGTCGCTTCGTCGAGCCGCGCGTGCTGGTCCGCCGCAAGGCGGGCGAGGTGGAGTACGTGCCCTCGTCTGAGGTGGACTACATGGACGTCTCG |
| CJH8-526-CTC | AGAAGCTCGGGCTGCATGTCGGCGAGCCCATCACGTCGTCGACGCTGACCGAAGAAGACGTCGTGGCCACCATCGAATATCTGGTCCGCTTGCACGAGGGTCAGACCACGATGACCGTTCCGGGCGGCGTCGAGGTGCCGGTGGAAACCGACGACATCGACCACTTCGGCAACCGCCGCCTGCGTACGGTCGGCGAGCTGATCCAAAACCAGATCCGGGTCGGCATGTCGCGGATGGAGCGGGTGGTCCGGGAGCGGATGACCACCCAGGACGTGGAGGCGATCACACCGCAGACGTTGATCAACATCCGGCCGGTGGTCGCCGCGATCAAGGAGTTCTTCGGCACCAGCCAGCTGAGCCAATTCATGGACCAGAACAACCCGCTGTCGGGGTTGACCCTCAAGCGCCGACTGTCGGCGCTGGGGCCCGGCGGTCTGTCACGTGAGCGTGCCGGGCTGGAGGTCCGCGACGTGCACCCGTCGCACTACGGCCGGATGTGCCCGATCGAAACCCCTGAGGGGCCCAACATCGGTCTGATCGGCTCGCTGTCGGTGTACGCGCGGGTCAACCCGTTCGGGTTCATCGAAACGCCGTACCGCAAGGTGGTCGACGGCGTGGTTAGCGACGAGATCGTGTACCTGACCGCCGACGAGGAGGACCGCCACGTGGTGGCACAGGCCAATTCGCCGATCGATGCGGACGGTCGCTTCGTCGAGCCGCGCGTGCTGGTCCGCCGCAAGGCGGGCGAGGTGGAGTACGTGCCCTCGTCTGAGGTGGACTACATGGACGTCTCG |
| CJH9-511-CCG | CCTGGCCCGCGTCGGTCGCTATAAGGTCAACAAGAAGCTCGGGCTGCATGTCGGCGAGCCCATCACGTCGTCGACGCTGACCGAAGAAGACGTCGTGGCCACCATCGAATATCTGGTCCGCTTGCACGAGGGTCAGACCACGATGACCGTTCCGGGCGGCGTCGAGGTGCCGGTGGAAACCGACGACATCGACCACTTCGGCAACCGCCGCCTGCGTACGGTCGGCGAGCTGATCCAAAACCAGATCCGGGTCGGCATGTCGCGGATGGAGCGGGTGGTCCGGGAGCGGATGACCACCCAGGACGTGGAGGCGATCACACCGCAGACGTTGATCAACATCCGGCCGGTGGTCGCCGCGATCAAGGAGTTCTTCGGCACCAGCCAGCCGAGCCAATTCATGGACCAGAACAACCCGCTGTCGGGGTTGACCCACAAGCGCCGACTGTCGGCGCTGGGGCCCGGCGGTCTGTCACGTGAGCGTGCCGGGCTGGAGGTCCGCGACGTGCACCCGTCGCACTACGGCCGGATGTGCCCGATCGAAACCCCTGAGGGGCCCAACATCGGTCTGATCGGCTCGCTGTCGGTGTACGCGCGGGTCAACCCGTTCGGGTTCATCGAAACGCCGTACCGCAAGGTGGTCGACGGCGTGGTTAGCGACGAGATCGTGTACCTGACCGCCGACGAGGAGGACCGCCACGTGGTGGCACAGGCCAATTCGCCGATCGATGCGGACGGTCGCTTCGTCGAGCCGCGCGTGCTGGTCCGCCGCAAGGCGGGCGAGGTGGAGTACGTGCC |
| CJH10-513-CCA | CACGTCGTCGACGCTGACCGAAGAAGACGTCGTGGCCACCATCGAATATCTGGTCCGCTTGCACGAGGGTCAGACCACGATGACCGTTCCGGGCGGCGTCGAGGTGCCGGTGGAAACCGACGACATCGACCACTTCGGCAACCGCCGCCTGCGTACGGTCGGCGAGCTGATCCAAAACCAGATCCGGGTCGGCATGTCGCGGATGGAGCGGGTGGTCCGGGAGCGGATGACCACCCAGGACGTGGAGGCGATCACACCGCAGACGTTGATCAACATCCGGCCGGTGGTCGCCGCGATCAAGGAGTTCTTCGGCACCAGCCAGCTGAGCCCATTCATGGGCCAGAACAACCCGCTGTCGGGGTTGACCCACAAGCGCCGACTGTCGGCGCTGGGGCCCGGCGGTCTGTCACGTGAGCGTGCCGGGCTGGAGGTCCGCGACGTGCACCCGTCGCACTACGGCCGGATGTGCCCGATCGAAACCCCTGAGGGGCCCAACATCGGTCTGATCGGCTCGCTGTCGGTGTACGCGCGGGTCAACCCGTTCGGGTTCATCGAAACGCCGTACCGCAAGGTGGTCGACGGCGTGGTTAGCGACGAGATCGTGTACCTGACCGCCGACGAGGAGGACCGCCACGTGGTGGCACAGGCCAATTCGCCGATCGATGCGGACGGTCGCTTCGTCGAGCCGCGCGTGCTGGTCCGCCGCAAGGCGGGCGAGGTGGAGTACGTGCCCTCGTCTGAGGTGGACTACATGGACGTCTCGCCCCGCCAGATGGTGTCGGTGGCCACCGC |
| CJH11-513-AAA | CACGTCGTCGACGCTGACCGAAGAAGACGTCGTGGCCACCATCGAATATCTGGTCCGCTTGCACGAGGGTCAGACCACGATGACCGTTCCGGGCGGCGTCGAGGTGCCGGTGGAAACCGACGACATCGACCACTTCGGCAACCGCCGCCTGCGTACGGTCGGCGAGCTGATCCAAAACCAGATCCGGGTCGGCATGTCGCGGATGGAGCGGGTGGTCCGGGAGCGGATGACCACCCAGGACGTGGAGGCGATCACACCGCAGACGTTGATCAACATCCGGCCGGTGGTCGCCGCGATCAAGGAGTTCTTCGGCACCAGCCAGCTGAGCAAATTCATGGGCCAGAACAACCCGCTGTCGGGGTTGACCCACAAGCGCCGACTGTCGGCGCTGGGGCCCGGCGGTCTGTCACGTGAGCGTGCCGGGCTGGAGGTCCGCGACGTGCACCCGTCGCACTACGGCCGGATGTGCCCGATCGAAACCCCTGAGGGGCCCAACATCGGTCTGATCGGCTCGCTGTCGGTGTACGCGCGGGTCAACCCGTTCGGGTTCATCGAAACGCCGTACCGCAAGGTGGTCGACGGCGTGGTTAGCGACGAGATCGTGTACCTGACCGCCGACGAGGAGGACCGCCACGTGGTGGCACAGGCCAATTCGCCGATCGATGCGGACGGTCGCTTCGTCGAGCCGCGCGTGCTGGTCCGCCGCAAGGCGGGCGAGGTGGAGTACGTGCCCTCGTCTGAGGTGGACTACATGGACGTCTCGCCCCGCCAGATGGTGTCGGTGGCCACCGC |
| CJH12-516-GTC | TGCATGTCGGCGAGCCCATCACGTCGTCGACGCTGACCGAAGAAGACGTCGTGGCCACCATCGAATATCTGGTCCGCTTGCACGAGGGTCAGACCACGATGACCGTTCCGGGCGGCGTCGAGGTGCCGGTGGAAACCGACGACATCGACCACTTCGGCAACCGCCGCCTGCGTACGGTCGGCGAGCTGATCCAAAACCAGATCCGGGTCGGCATGTCGCGGATGGAGCGGGTGGTCCGGGAGCGGATGACCACCCAGGACGTGGAGGCGATCACACCGCAGACGTTGATCAACATCCGGCCGGTGGTCGCCGCGATCAAGGAGTTCTTCGGCACCAGCCAGCTGAGCCAATTCATGGTCCAGAACAACCCGCTGTCGGGGTTGACCCACAAGCGCCGACTGTCGGCGCTGGGGCCCGGCGGTCTGTCACGTGAGCGTGCCGGGCTGGAGGTCCGCGACGTGCACCCGTCGCACTACGGCCGGATGTGCCCGATCGAAACCCCTGAGGGGCCCAACATCGGTCTGATCGGCTCGCTGTCGGTGTACGCGCGGGTCAACCCGTTCGGGTTCATCGAAACGCCGTACCGCAAGGTGGTCGACGGCGTGGTTAGCGACGAGATCGTGTACCTGACCGCCGACGAGGAGGACCGCCACGTGGTGGCACAGGCCAATTCGCCGATCGATGCGGACGGTCGCTTCGTCGAGCCGCGCGTGCTGGTCCGCCGCAAGGCGGGCGAGGTGGAGTACGTGCCCTCGTCTGAGGTGGACTACATGGACG |
| CJH13-516-TAC | TGCATGTCGGCGAGCCCATCACGTCGTCGACGCTGACCGAAGAAGACGTCGTGGCCACCATCGAATATCTGGTCCGCTTGCACGAGGGTCAGACCACGATGACCGTTCCGGGCGGCGTCGAGGTGCCGGTGGAAACCGACGACATCGACCACTTCGGCAACCGCCGCCTGCGTACGGTCGGCGAGCTGATCCAAAACCAGATCCGGGTCGGCATGTCGCGGATGGAGCGGGTGGTCCGGGAGCGGATGACCACCCAGGACGTGGAGGCGATCACACCGCAGACGTTGATCAACATCCGGCCGGTGGTCGCCGCGATCAAGGAGTTCTTCGGCACCAGCCAGCTGAGCCAATTCATGTACCAGAACAACCCGCTGTCGGGGTTGACCCACAAGCGCCGACTGTCGGCGCTGGGGCCCGGCGGTCTGTCACGTGAGCGTGCCGGGCTGGAGGTCCGCGACGTGCACCCGTCGCACTACGGCCGGATGTGCCCGATCGAAACCCCTGAGGGGCCCAACATCGGTCTGATCGGCTCGCTGTCGGTGTACGCGCGGGTCAACCCGTTCGGGTTCATCGAAACGCCGTACCGCAAGGTGGTCGACGGCGTGGTTAGCGACGAGATCGTGTACCTGACCGCCGACGAGGAGGACCGCCACGTGGTGGCACAGGCCAATTCGCCGATCGATGCGGACGGTCGCTTCGTCGAGCCGCGCGTGCTGGTCCGCCGCAAGGCGGGCGAGGTGGAGTACGTGCCCTCGTCTGAGGTGGACTACATGGACG |
| CJH14-516-GGC | TGCATGTCGGCGAGCCCATCACGTCGTCGACGCTGACCGAAGAAGACGTCGTGGCCACCATCGAATATCTGGTCCGCTTGCACGAGGGTCAGACCACGATGACCGTTCCGGGCGGCGTCGAGGTGCCGGTGGAAACCGACGACATCGACCACTTCGGCAACCGCCGCCTGCGTACGGTCGGCGAGCTGATCCAAAACCAGATCCGGGTCGGCATGTCGCGGATGGAGCGGGTGGTCCGGGAGCGGATGACCACCCAGGACGTGGAGGCGATCACACCGCAGACGTTGATCAACATCCGGCCGGTGGTCGCCGCGATCAAGGAGTTCTTCGGCACCAGCCAGCTGAGCCAATTCATGGGCCAGAACAACCCGCTGTCGGGGTTGACCCACAAGCGCCGACTGTCGGCGCTGGGGCCCGGCGGTCTGTCACGTGAGCGTGCCGGGCTGGAGGTCCGCGACGTGCACCCGTCGCACTACGGCCGGATGTGCCCGATCGAAACCCCTGAGGGGCCCAACATCGGTCTGATCGGCTCGCTGTCGGTGTACGCGCGGGTCAACCCGTTCGGGTTCATCGAAACGCCGTACCGCAAGGTGGTCGACGGCGTGGTTAGCGACGAGATCGTGTACCTGACCGCCGACGAGGAGGACCGCCACGTGGTGGCACAGGCCAATTCGCCGATCGATGCGGACGGTCGCTTCGTCGAGCCGCGCGTGCTGGTCCGCCGCAAGGCGGGCGAGGTGGAGTACGTGCCCTCGTCTGAGGTGGACTACATGGACG |
| CJH15-533-CCG | CACGTCGTCGACGCTGACCGAAGAAGACGTCGTGGCCACCATCGAATATCTGGTCCGCTTGCACGAGGGTCAGACCACGATGACCGTTCCGGGCGGCGTCGAGGTGCCGGTGGAAACCGACGACATCGACCACTTCGGCAACCGCCGCCTGCGTACGGTCGGCGAGCTGATCCAAAACCAGATCCGGGTCGGCATGTCGCGGATGGAGCGGGTGGTCCGGGAGCGGATGACCACCCAGGACGTGGAGGCGATCACACCGCAGACGTTGATCAACATCCGGCCGGTGGTCGCCGCGATCAAGGAGTTCTTCGGCACCAGCCAGCTGAGCCAATTCATGGACCAGAACAACCCGCTGTCGGGGTTGACCCACAAGCGCCGACTGTCGGCGCCGGGGCCCGGCGGTCTGTCACGTGAGCGTGCCGGGCTGGAGGTCCGCGACGTGCACCCGTCGCACTACGGCCGGATGTGCCCGATCGAAACCCCTGAGGGGCCCAACATCGGTCTGATCGGCTCGCTGTCGGTGTACGCGCGGGTCAACCCGTTCGGGTTCATCGAAACGCCGTACCGCAAGGTGGTCGACGGCGTGGTTAGCGACGAGATCGTGTACCTGACCGCCGACGAGGAGGACCGCCACGTGGTGGCACAGGCCAATTCGCCGATCGATGCGGACGGTCGCTTCGTCGAGCCGCGCGTGCTGGTCCGCCGCAAGGCGGGCGAGGTGGAGTACGTGCCCTCGTCTGAGGTGGACTACATGGACGTCTCGCCCCGCCAGATGGTGTCGGTGGCCACCGC |
| CJH31-513-CTA | CACGTCGTCGACGCTGACCGAAGAAGACGTCGTGGCCACCATCGAATATCTGGTCCGCTTGCACGAGGGTCAGACCACGATGACCGTTCCGGGCGGCGTCGAGGTGCCGGTGGAAACCGACGACATCGACCACTTCGGCAACCGCCGCCTGCGTACGGTCGGCGAGCTGATCCAAAACCAGATCCGGGTCGGCATGTCGCGGATGGAGCGGGTGGTCCGGGAGCGGATGACCACCCAGGACGTGGAGGCGATCACACCGCAGACGTTGATCAACATCCGGCCGGTGGTCGCCGCGATCAAGGAGTTCTTCGGCACCAGCCAGCTGAGCCTATTCATGGGCCAGAACAACCCGCTGTCGGGGTTGACCCACAAGCGCCGACTGTCGGCGCTGGGGCCCGGCGGTCTGTCACGTGAGCGTGCCGGGCTGGAGGTCCGCGACGTGCACCCGTCGCACTACGGCCGGATGTGCCCGATCGAAACCCCTGAGGGGCCCAACATCGGTCTGATCGGCTCGCTGTCGGTGTACGCGCGGGTCAACCCGTTCGGGTTCATCGAAACGCCGTACCGCAAGGTGGTCGACGGCGTGGTTAGCGACGAGATCGTGTACCTGACCGCCGACGAGGAGGACCGCCACGTGGTGGCACAGGCCAATTCGCCGATCGATGCGGACGGTCGCTTCGTCGAGCCGCGCGTGCTGGTCCGCCGCAAGGCGGGCGAGGTGGAGTACGTGCCCTCGTCTGAGGTGGACTACATGGACGTCTCGCCCCGCCAGATGGTGTCGGTGGCCACCGC |
| CJH32-522-TTG | CACGTCGTCGACGCTGACCGAAGAAGACGTCGTGGCCACCATCGAATATCTGGTCCGCTTGCACGAGGGTCAGACCACGATGACCGTTCCGGGCGGCGTCGAGGTGCCGGTGGAAACCGACGACATCGACCACTTCGGCAACCGCCGCCTGCGTACGGTCGGCGAGCTGATCCAAAACCAGATCCGGGTCGGCATGTCGCGGATGGAGCGGGTGGTCCGGGAGCGGATGACCACCCAGGACGTGGAGGCGATCACACCGCAGACGTTGATCAACATCCGGCCGGTGGTCGCCGCGATCAAGGAGTTCTTCGGCACCAGCCAGCTGAGCCAATTCATGGACCAGAACAACCCGCTGTTGGGGTTGACCCACAAGCGCCGACTGTCGGCGCTGGGGCCCGGCGGTCTGTCACGTGAGCGTGCCGGGCTGGAGGTCCGCGACGTGCACCCGTCGCACTACGGCCGGATGTGCCCGATCGAAACCCCTGAGGGGCCCAACATCGGTCTGATCGGCTCGCTGTCGGTGTACGCGCGGGTCAACCCGTTCGGGTTCATCGAAACGCCGTACCGCAAGGTGGTCGACGGCGTGGTTAGCGACGAGATCGTGTACCTGACCGCCGACGAGGAGGACCGCCACGTGGTGGCACAGGCCAATTCGCCGATCGATGCGGACGGTCGCTTCGTCGAGCCGCGCGTGCTGGTCCGCCGCAAGGCGGGCGAGGTGGAGTACGTGCCCTCGTCTGAGGTGGACTACATGGACGTCTCGCCCCGCCAGATGGTGTCGGTGGCCACCGC |
| CJH33-526-CGC | AGAAGCTCGGGCTGCATGTCGGCGAGCCCATCACGTCGTCGACGCTGACCGAAGAAGACGTCGTGGCCACCATCGAATATCTGGTCCGCTTGCACGAGGGTCAGACCACGATGACCGTTCCGGGCGGCGTCGAGGTGCCGGTGGAAACCGACGACATCGACCACTTCGGCAACCGCCGCCTGCGTACGGTCGGCGAGCTGATCCAAAACCAGATCCGGGTCGGCATGTCGCGGATGGAGCGGGTGGTCCGGGAGCGGATGACCACCCAGGACGTGGAGGCGATCACACCGCAGACGTTGATCAACATCCGGCCGGTGGTCGCCGCGATCAAGGAGTTCTTCGGCACCAGCCAGCTGAGCCAATTCATGGACCAGAACAACCCGCTGTCGGGGTTGACCCGCAAGCGCCGACTGTCGGCGCTGGGGCCCGGCGGTCTGTCACGTGAGCGTGCCGGGCTGGAGGTCCGCGACGTGCACCCGTCGCACTACGGCCGGATGTGCCCGATCGAAACCCCTGAGGGGCCCAACATCGGTCTGATCGGCTCGCTGTCGGTGTACGCGCGGGTCAACCCGTTCGGGTTCATCGAAACGCCGTACCGCAAGGTGGTCGACGGCGTGGTTAGCGACGAGATCGTGTACCTGACCGCCGACGAGGAGGACCGCCACGTGGTGGCACAGGCCAATTCGCCGATCGATGCGGACGGTCGCTTCGTCGAGCCGCGCGTGCTGGTCCGCCGCAAGGCGGGCGAGGTGGAGTACGTGCCCTCGTCTGAGGTGGACTACATGGACGTCTCG |
| CJH84-510-512del | CACGTCGTCGACGCTGACCGAAGAAGACGTCGTGGCCACCATCGAATATCTGGTCCGCTTGCACGAGGGTCAGACCACGATGACCGTTCCGGGCGGCGTCGAGGTGCCGGTGGAAACCGACGACATCGACCACTTCGGCAACCGCCGCCTGCGTACGGTCGGCGAGCTGATCCAAAACCAGATCCGGGTCGGCATGTCGCGGATGGAGCGGGTGGTCCGGGAGCGGATGACCACCCAGGACGTGGAGGCGATCACACCGCAGACGTTGATCAACATCCGGCCGGTGGTCGCCGCGATCAAGGAGTTCTTCGGCACCAGCCAATTCATGGACCAGAACAACCCGCTGTCGGGGTTGACCCACAAGCGCCGACTGTCGGCGCTGGGGCCCGGCGGTCTGTCACGTGAGCGTGCCGGGCTGGAGGTCCGCGACGTGCACCCGTCGCACTACGGCCGGATGTGCCCGATCGAAACCCCTGAGGGGCCCAACATCGGTCTGATCGGCTCGCTGTCGGTGTACGCGCGGGTCAACCCGTTCGGGTTCATCGAAACGCCGTACCGCAAGGTGGTCGACGGCGTGGTTAGCGACGAGATCGTGTACCTGACCGCCGACGAGGAGGACCGCCACGTGGTGGCACAGGCCAATTCGCCGATCGATGCGGACGGTCGCTTCGTCGAGCCGCGCGTGCTGGTCCGCCGCAAGGCGGGCGAGGTGGAGTACGTGCCCTCGTCTGAGGTGGACTACATGGACGTCTCGCCCCGCCAGATGGTGTCGGTGGCCACCGC |
| CJH85-517-518del | CACGTCGTCGACGCTGACCGAAGAAGACGTCGTGGCCACCATCGAATATCTGGTCCGCTTGCACGAGGGTCAGACCACGATGACCGTTCCGGGCGGCGTCGAGGTGCCGGTGGAAACCGACGACATCGACCACTTCGGCAACCGCCGCCTGCGTACGGTCGGCGAGCTGATCCAAAACCAGATCCGGGTCGGCATGTCGCGGATGGAGCGGGTGGTCCGGGAGCGGATGACCACCCAGGACGTGGAGGCGATCACACCGCAGACGTTGATCAACATCCGGCCGGTGGTCGCCGCGATCAAGGAGTTCTTCGGCACCAGCCAGCTGAGCCAATTCATGGACAACCCGCTGTCGGGGTTGACCCACAAGCGCCGACTGTCGGCGCTGGGGCCCGGCGGTCTGTCACGTGAGCGTGCCGGGCTGGAGGTCCGCGACGTGCACCCGTCGCACTACGGCCGGATGTGCCCGATCGAAACCCCTGAGGGGCCCAACATCGGTCTGATCGGCTCGCTGTCGGTGTACGCGCGGGTCAACCCGTTCGGGTTCATCGAAACGCCGTACCGCAAGGTGGTCGACGGCGTGGTTAGCGACGAGATCGTGTACCTGACCGCCGACGAGGAGGACCGCCACGTGGTGGCACAGGCCAATTCGCCGATCGATGCGGACGGTCGCTTCGTCGAGCCGCGCGTGCTGGTCCGCCGCAAGGCGGGCGAGGTGGAGTACGTGCCCTCGTCTGAGGTGGACTACATGGACGTCTCGCCCCGCCAGATGGTGTCGGTGGCCACCGC |
| CJH16 rpoB531 TCG-TTT | GTCGCTATAAGGTCAACAAGAAGCTCGGGCTGCATGTCGGCGAGCCCATCACGTCGTCGACGCTGACCGAAGAAGACGTCGTGGCCACCATCGAATATCTGGTCCGCTTGCACGAGGGTCAGACCACGATGACCGTTCCGGGCGGCGTCGAGGTGCCGGTGGAAACCGACGACATCGACCACTTCGGCAACCGCCGCCTGCGTACGGTCGGCGAGCTGATCCAAAACCAGATCCGGGTCGGCATGTCGCGGATGGAGCGGGTGGTCCGGGAGCGGATGACCACCCAGGACGTGGAGGCGATCACACCGCAGACGTTGATCAACATCCGGCCGGTGGTCGCCGCGATCAAGGAGTTCTTCGGCACCAGCCAGCTGAGCCAATTCATGGACCAGAACAACCCGCTGTCGGGGTTGACCCACAAGCGCCGACTGTTTGCGCTGGGGCCCGGCGGTCTGTCACGTGAGCGTGCCGGGCTGGAGGTCCGCGACGTGCACCCGTCGCACTACGGCCGGATGTGCCCGATCGAAACCCCTGAGGGGCCCAACATCGGTCTGATCGGCTCGCTGTCGGTGTACGCGCGGGTCAACCCGTTCGGGTTCATCGAAACGCCGTACCGCAAGGTGGTCGACGGCGTGGTTAGCGACGAGATCGTGTACCTGACCGCCGACGAGGAGGACCGCCACGTGGTGGCACAGGCCAATTCGCCGATCGATGCGGACGGTCGCTTCGTCGAGCCGCGCGTGCTGGTCCGCCGCAAGGCGGGCGAGGTGGAGTACGTGCCCTCGTCTGAGGTGGACT |
| W3 | CACGTCGTCGACGCTGACCGAAGAAGACGTCGTGGCCACCATCGAATATCTGGTCCGCTTGCACGAGGGTCAGACCACGATGACCGTTCCGGGCGGCGTCGAGGTGCCGGTGGAAACCGACGACATCGACCACTTCGGCAACCGCCGCCTGCGTACGGTCGGCGAGCTGATCCAAAACCAGATCCGGGTCGGCATGTCGCGGATGGAGCGGGTGGTCCGGGAGCGGATGACCACCCAGGACGTGGAGGCGATCACACCGCAGACGTTGATCAACATCCGGCCGGTGGTCGCCGCGATCAAGGAGTTCTTCGGCACCAGCCAGCTGAGCCAATTCATGGACCAGAACAACCCGCTGTCGGGGTTGACCCACAAGCGCCGACTGTCGGCGCTGGGGCCCGGCGGTCTGTCACGTGAGCGTGCCGGGCTGGAGGTCCGCGACGTGCACCCGTCGCACTACGGCCGGATGTGCCCGATCGAAACCCCTGAGGGGCCCAACATCGGTCTGATCGGCTCGCTGTCGGTGTACGCGCGGGTCAACCCGTTCGGGTTCATCGAAACGCCGTACCGCAAGGTGGTCGACGGCGTGGTTAGCGACGAGATCGTGTACCTGACCGCCGACGAGGAGGACCGCCACGTGGTGGCACAGGCCAATTCGCCGATCGATGCGGACGGTCGCTTCGTCGAGCCGCGCGTGCTGGTCCGCCGCAAGGCGGGCGAGGTGGAGTACGTGCCCTCGTCTGAGGTGGACTACATGGACGTCTCGCCCCGCCAGATGGTGTCGGTGGCCACCGC |
| CJH1- 315ACC | AGCCCGATGAGGTCTATTGGGGCAAGGAAGCCACCTGGCTCGGCGATGAGCGTTACAGCGGTAAGCGGGATCTGGAGAACCCGCTGGCCGCGGTGCAGATGGGGCTGATCTACGTGAACCCGGAGGGGCCGAACGGCAACCCGGACCCCATGGCCGCGGCGGTCGACATTCGCGAGACGTTTCGGCGCATGGCCATGAACGACGTCGAAACAGCGGCGCTGATCGTCGGCGGTCACACTTTCGGTAAGACCCATGGCGCCGGCCCGGCCGATCTGGTCGGCCCCGAACCCGAGGCTGCTCCGCTGGAGCAGATGGGCTTGGGCTGGAAGAGCTCGTATGGCACCGGAACCGGTAAGGACGCGATCACCACCGGCATCGAGGTCGTATGGACGAACACCCCGACGAAATGGGACAACAGTTTCCTCGAGATCCTGTACGGCTACGAGTGGGAGCTGACGAAGAGCCCTGCTGGCGCTTGGCAATACACCGCCAAGGACGGCGCCGGTGCCGGCACCATCCCGGACCCGTTCGGCGGGCCAGGGCGCTCCCCGACGATGCTGGCCACTGACCTCTCGCTGCGGGTGGATCCGATCTATGAGCGGATCACGCGTCGCTGGCTGGAACACCCCGAGGAATTGGCCGACGAGTTCGCCAAGGCCTGGTACAAGCTGATCCACCGAGACATGGGTCCCGTTGCGAGATACCTTGGGCCGCTGGTCCCCAAGCAGACCCTGCTGTGGCAGGATCCGGTCCCTGCGGTCAGCCACGACCTCGTCGG |
| CJH2- 315AAC | AGCCCGATGAGGTCTATTGGGGCAAGGAAGCCACCTGGCTCGGCGATGAGCGTTACAGCGGTAAGCGGGATCTGGAGAACCCGCTGGCCGCGGTGCAGATGGGGCTGATCTACGTGAACCCGGAGGGGCCGAACGGCAACCCGGACCCCATGGCCGCGGCGGTCGACATTCGCGAGACGTTTCGGCGCATGGCCATGAACGACGTCGAAACAGCGGCGCTGATCGTCGGCGGTCACACTTTCGGTAAGACCCATGGCGCCGGCCCGGCCGATCTGGTCGGCCCCGAACCCGAGGCTGCTCCGCTGGAGCAGATGGGCTTGGGCTGGAAGAGCTCGTATGGCACCGGAACCGGTAAGGACGCGATCACCAACGGCATCGAGGTCGTATGGACGAACACCCCGACGAAATGGGACAACAGTTTCCTCGAGATCCTGTACGGCTACGAGTGGGAGCTGACGAAGAGCCCTGCTGGCGCTTGGCAATACACCGCCAAGGACGGCGCCGGTGCCGGCACCATCCCGGACCCGTTCGGCGGGCCAGGGCGCTCCCCGACGATGCTGGCCACTGACCTCTCGCTGCGGGTGGATCCGATCTATGAGCGGATCACGCGTCGCTGGCTGGAACACCCCGAGGAATTGGCCGACGAGTTCGCCAAGGCCTGGTACAAGCTGATCCACCGAGACATGGGTCCCGTTGCGAGATACCTTGGGCCGCTGGTCCCCAAGCAGACCCTGCTGTGGCAGGATCCGGTCCCTGCGGTCAGCCACGACCTCGTCGG |
| CJH29- 316AGC | AGCCCGATGAGGTCTATTGGGGCAAGGAAGCCACCTGGCTCGGCGATGAGCGTTACAGCGGTAAGCGGGATCTGGAGAACCCGCTGGCCGCGGTGCAGATGGGGCTGATCTACGTGAACCCGGAGGGGCCGAACGGCAACCCGGACCCCATGGCCGCGGCGGTCGACATTCGCGAGACGTTTCGGCGCATGGCCATGAACGACGTCGAAACAGCGGCGCTGATCGTCGGCGGTCACACTTTCGGTAAGACCCATGGCGCCGGCCCGGCCGATCTGGTCGGCCCCGAACCCGAGGCTGCTCCGCTGGAGCAGATGGGCTTGGGCTGGAAGAGCTCGTATGGCACCGGAACCGGTAAGGACGCGATCACCAGCAGCATCGAGGTCGTATGGACGAACACCCCGACGAAATGGGACAACAGTTTCCTCGAGATCCTGTACGGCTACGAGTGGGAGCTGACGAAGAGCCCTGCTGGCGCTTGGCAATACACCGCCAAGGACGGCGCCGGTGCCGGCACCATCCCGGACCCGTTCGGCGGGCCAGGGCGCTCCCCGACGATGCTGGCCACTGACCTCTCGCTGCGGGTGGATCCGATCTATGAGCGGATCACGCGTCGCTGGCTGGAACACCCCGAGGAATTGGCCGACGAGTTCGCCAAGGCCTGGTACAAGCTGATCCACCGAGACATGGGTCCCGTTGCGAGATACCTTGGGCCGCTGGTCCCCAAGCAGACCCTGCTGTGGCAGGATCCGGTCCCTGCGGTCAGCCACGACCTCGTCGG |
| CJH30- 316GAC | AGCCCGATGAGGTCTATTGGGGCAAGGAAGCCACCTGGCTCGGCGATGAGCGTTACAGCGGTAAGCGGGATCTGGAGAACCCGCTGGCCGCGGTGCAGATGGGGCTGATCTACGTGAACCCGGAGGGGCCGAACGGCAACCCGGACCCCATGGCCGCGGCGGTCGACATTCGCGAGACGTTTCGGCGCATGGCCATGAACGACGTCGAAACAGCGGCGCTGATCGTCGGCGGTCACACTTTCGGTAAGACCCATGGCGCCGGCCCGGCCGATCTGGTCGGCCCCGAACCCGAGGCTGCTCCGCTGGAGCAGATGGGCTTGGGCTGGAAGAGCTCGTATGGCACCGGAACCGGTAAGGACGCGATCACCAGCGACATCGAGGTCGTATGGACGAACACCCCGACGAAATGGGACAACAGTTTCCTCGAGATCCTGTACGGCTACGAGTGGGAGCTGACGAAGAGCCCTGCTGGCGCTTGGCAATACACCGCCAAGGACGGCGCCGGTGCCGGCACCATCCCGGACCCGTTCGGCGGGCCAGGGCGCTCCCCGACGATGCTGGCCACTGACCTCTCGCTGCGGGTGGATCCGATCTATGAGCGGATCACGCGTCGCTGGCTGGAACACCCCGAGGAATTGGCCGACGAGTTCGCCAAGGCCTGGTACAAGCTGATCCACCGAGACATGGGTCCCGTTGCGAGATACCTTGGGCCGCTGGTCCCCAAGCAGACCCTGCTGTGGCAGGATCCGGTCCCTGCGGTCAGCCACGACCTCGTCGG |
| CJH3- -15T | GGGCACGGCGGCGGTTGGTGTCGATGATCGCGGCGGGAAGATCCGCGTCGATCCACTTGGCGCCATGGAAGGCAGAAGCCGAGTAGCCGGCCAGCACGCCGCGGCGGCGCGAGCGCAGCCACAGCGCTTTTGCACGCAATTGCGCGGTCAGTTCCACACCCTGCGGCACGTACACGTCTTTATGTAGCGCGACATACCTGCTGCGCAATTCGTAGGGCGTCAATACACCCGCAGCCAGGGCCTCGCTGCCCAGAAAGGGATCCGTCATGGTCGAAGTGTGCTGAGTCACACCGACAAACGTCACGAGCGTAACCCCAGTGCGAAAGTTCCCGCCGGAAATCGCAGCCACGTTACGCTCGTGGACATACCGATTTCGGCCCGGCCGCGGCGAGATGATAGGTTGTCGGGGTGACTGCCACAGCCACTGAAGGGGCCAAACCCCCATTCGTATCCCGTTCAGTCCTGGTTACCGGAGGAAACCGGGGGATCGGGCTGGCGATCGCACAGCGGCTGGCTGCCGACGGCCACAAGGTGGCCGTCACCCACCGTGGATCCGGAGCGCCAAAGGGGCTGTTTGGCGTCGAATGTGACGTCACCGACAGCGACGCCGTCGATCGCGCCTTCACGGCGGTAGAAGAGCACCAGGGTCCGGTCGAGGTGCTGGTGTCCAACGCCGGCCTATCCGCGGACGCATTCCTCATGCGGATGACCGAGGAAAAGTTCGAGAAGGTCATCAACGCCAACCTCACCGGGGCGTTCCGGGTGGCTCAACGGGCATCGCGCAGCATGCAGCG |
| CJH24 - -8A | GGGCACGGCGGCGGTTGGTGTCGATGATCGCGGCGGGAAGATCCGCGTCGATCCACTTGGCGCCATGGAAGGCAGAAGCCGAGTAGCCGGCCAGCACGCCGCGGCGGCGCGAGCGCAGCCACAGCGCTTTTGCACGCAATTGCGCGGTCAGTTCCACACCCTGCGGCACGTACACGTCTTTATGTAGCGCGACATACCTGCTGCGCAATTCGTAGGGCGTCAATACACCCGCAGCCAGGGCCTCGCTGCCCAGAAAGGGATCCGTCATGGTCGAAGTGTGCTGAGTCACACCGACAAACGTCACGAGCGTAACCCCAGTGCGAAAGTTCCCGCCGGAAATCGCAGCCACGTTACGCTCGTGGACATACCGATTTCGGCCCGGCCGCGGCGAGACGATAGGATGTCGGGGTGACTGCCACAGCCACTGAAGGGGCCAAACCCCCATTCGTATCCCGTTCAGTCCTGGTTACCGGAGGAAACCGGGGGATCGGGCTGGCGATCGCACAGCGGCTGGCTGCCGACGGCCACAAGGTGGCCGTCACCCACCGTGGATCCGGAGCGCCAAAGGGGCTGTTTGGCGTCGAATGTGACGTCACCGACAGCGACGCCGTCGATCGCGCCTTCACGGCGGTAGAAGAGCACCAGGGTCCGGTCGAGGTGCTGGTGTCCAACGCCGGCCTATCCGCGGACGCATTCCTCATGCGGATGACCGAGGAAAAGTTCGAGAAGGTCATCAACGCCAACCTCACCGGGGCGTTCCGGGTGGCTCAACGGGCATCGCGCAGCATGCAGCG |
| CJH56- -46 A | gagaccggcttccgaccaccgctcgccgcaacgtcgactggctcatatcgagaatgcttgcggcactgctgaaccactgctttgccgccaccgcggcgaacgcgcgaagcccggccacggccggctagcacctcttggcggcgatgccgataaatatggtgtAatatatcacctttgcctgacagcgacttcacggcacgatggaatgtcgcaaccaaatgcattgtccgctttgatgatgaggagagtcatgccactgctaaccattggcgatcaattccccgcctaccagctcaccgctctcatcggcggtgacctgtccaaggtcgacgccaagcagcccggcgactacttcaccactatcaccagtgacgaacacccaggcaagtggcgggtggtgttcttttggccgaaagacttcacgttcgtgtgccctaccgagatcgcggcgttcagcaagctcaatgacgagttcgaggaccgcgacgcccagatcctgggggtttcgattgacagcgaattcgcgcatttccagtggcgtgcacagcacaacgacctcaaaacgttacccttcccgatgctctccgacatcaagcgcgaactcagccaagccgcaggtgtcctcaacgccgacggtgtggccgaccgcgtgacctttatcgtcgaccccaacaacgagatccagttcgtctcggccaccgccggttcggtgggacgcaacgtcgatgaggtactgcgagtgctcgacgccctccagtccgacgagctgtgcgcatgcaactggcgcaagggcgacccgacgctagacgctggcgaactcctcaaggcttcggcctaa |
| CJH57- -44 A | gagaccggcttccgaccaccgctcgccgcaacgtcgactggctcatatcgagaatgcttgcggcactgctgaaccactgctttgccgccaccgcggcgaacgcgcgaagcccggccacggccggctagcacctcttggcggcgatgccgataaatatggtgtgaAatatcacctttgcctgacagcgacttcacggcacgatggaatgtcgcaaccaaatgcattgtccgctttgatgatgaggagagtcatgccactgctaaccattggcgatcaattccccgcctaccagctcaccgctctcatcggcggtgacctgtccaaggtcgacgccaagcagcccggcgactacttcaccactatcaccagtgacgaacacccaggcaagtggcgggtggtgttcttttggccgaaagacttcacgttcgtgtgccctaccgagatcgcggcgttcagcaagctcaatgacgagttcgaggaccgcgacgcccagatcctgggggtttcgattgacagcgaattcgcgcatttccagtggcgtgcacagcacaacgacctcaaaacgttacccttcccgatgctctccgacatcaagcgcgaactcagccaagccgcaggtgtcctcaacgccgacggtgtggccgaccgcgtgacctttatcgtcgaccccaacaacgagatccagttcgtctcggccaccgccggttcggtgggacgcaacgtcgatgaggtactgcgagtgctcgacgccctccagtccgacgagctgtgcgcatgcaactggcgcaagggcgacccgacgctagacgctggcgaactcctcaaggcttcggcctaa |
| CJH58- -40C | gagaccggcttccgaccaccgctcgccgcaacgtcgactggctcatatcgagaatgcttgcggcactgctgaaccactgctttgccgccaccgcggcgaacgcgcgaagcccggccacggccggctagcacctcttggcggcgatgccgataaatatggtgtgaTataCcacctttgcctgacagcgacttcacggcacgatggaatgtcgcaaccaaatgcattgtccgctttgatgatgaggagagtcatgccactgctaaccattggcgatcaattccccgcctaccagctcaccgctctcatcggcggtgacctgtccaaggtcgacgccaagcagcccggcgactacttcaccactatcaccagtgacgaacacccaggcaagtggcgggtggtgttcttttggccgaaagacttcacgttcgtgtgccctaccgagatcgcggcgttcagcaagctcaatgacgagttcgaggaccgcgacgcccagatcctgggggtttcgattgacagcgaattcgcgcatttccagtggcgtgcacagcacaacgacctcaaaacgttacccttcccgatgctctccgacatcaagcgcgaactcagccaagccgcaggtgtcctcaacgccgacggtgtggccgaccgcgtgacctttatcgtcgaccccaacaacgagatccagttcgtctcggccaccgccggttcggtgggacgcaacgtcgatgaggtactgcgagtgctcgacgccctccagtccgacgagctgtgcgcatgcaactggcgcaagggcgacccgacgctagacgctggcgaactcctcaaggcttcggcctaa |
| CJH59- -39 T | gagaccggcttccgaccaccgctcgccgcaacgtcgactggctcatatcgagaatgcttgcggcactgctgaaccactgctttgccgccaccgcggcgaacgcgcgaagcccggccacggccggctagcacctcttggcggcgatgccgataaatatggtgtgatatattacctttgcctgacagcgacttcacggcacgatggaatgtcgcaaccaaatgcattgtccgctttgatgatgaggagagtcatgccactgctaaccattggcgatcaattccccgcctaccagctcaccgctctcatcggcggtgacctgtccaaggtcgacgccaagcagcccggcgactacttcaccactatcaccagtgacgaacacccaggcaagtggcgggtggtgttcttttggccgaaagacttcacgttcgtgtgccctaccgagatcgcggcgttcagcaagctcaatgacgagttcgaggaccgcgacgcccagatcctgggggtttcgattgacagcgaattcgcgcatttccagtggcgtgcacagcacaacgacctcaaaacgttacccttcccgatgctctccgacatcaagcgcgaactcagccaagccgcaggtgtcctcaacgccgacggtgtggccgaccgcgtgacctttatcgtcgaccccaacaacgagatccagttcgtctcggccaccgccggttcggtgggacgcaacgtcgatgaggtactgcgagtgctcgacgccctccagtccgacgagctgtgcgcatgcaactggcgcaagggcgacccgacgctagacgctggcgaactcctcaaggcttcggcctaa |
| CJH60- -34 A | gagaccggcttccgaccaccgctcgccgcaacgtcgactggctcatatcgagaatgcttgcggcactgctgaaccactgctttgccgccaccgcggcgaacgcgcgaagcccggccacggccggctagcacctcttggcggcgatgccgataaatatggtgtgatatatcacctAtgcctgacagcgacttcacggcacgatggaatgtcgcaaccaaatgcattgtccgctttgatgatgaggagagtcatgccactgctaaccattggcgatcaattccccgcctaccagctcaccgctctcatcggcggtgacctgtccaaggtcgacgccaagcagcccggcgactacttcaccactatcaccagtgacgaacacccaggcaagtggcgggtggtgttcttttggccgaaagacttcacgttcgtgtgccctaccgagatcgcggcgttcagcaagctcaatgacgagttcgaggaccgcgacgcccagatcctgggggtttcgattgacagcgaattcgcgcatttccagtggcgtgcacagcacaacgacctcaaaacgttacccttcccgatgctctccgacatcaagcgcgaactcagccaagccgcaggtgtcctcaacgccgacggtgtggccgaccgcgtgacctttatcgtcgaccccaacaacgagatccagttcgtctcggccaccgccggttcggtgggacgcaacgtcgatgaggtactgcgagtgctcgacgccctccagtccgacgagctgtgcgcatgcaactggcgcaagggcgacccgacgctagacgctggcgaactcctcaaggcttcggcctaa |
| CJH61- -34 C | gagaccggcttccgaccaccgctcgccgcaacgtcgactggctcatatcgagaatgcttgcggcactgctgaaccactgctttgccgccaccgcggcgaacgcgcgaagcccggccacggccggctagcacctcttggcggcgatgccgataaatatggtgtgatatatcacctCtgcctgacagcgacttcacggcacgatggaatgtcgcaaccaaatgcattgtccgctttgatgatgaggagagtcatgccactgctaaccattggcgatcaattccccgcctaccagctcaccgctctcatcggcggtgacctgtccaaggtcgacgccaagcagcccggcgactacttcaccactatcaccagtgacgaacacccaggcaagtggcgggtggtgttcttttggccgaaagacttcacgttcgtgtgccctaccgagatcgcggcgttcagcaagctcaatgacgagttcgaggaccgcgacgcccagatcctgggggtttcgattgacagcgaattcgcgcatttccagtggcgtgcacagcacaacgacctcaaaacgttacccttcccgatgctctccgacatcaagcgcgaactcagccaagccgcaggtgtcctcaacgccgacggtgtggccgaccgcgtgacctttatcgtcgaccccaacaacgagatccagttcgtctcggccaccgccggttcggtgggacgcaacgtcgatgaggtactgcgagtgctcgacgccctccagtccgacgagctgtgcgcatgcaactggcgcaagggcgacccgacgctagacgctggcgaactcctcaaggcttcggcctaa |
| CJH62- -32 A | gagaccggcttccgaccaccgctcgccgcaacgtcgactggctcatatcgagaatgcttgcggcactgctgaaccactgctttgccgccaccgcggcgaacgcgcgaagcccggccacggccggctagcacctcttggcggcgatgccgataaatatggtgtgatatatcacctttAcctgacagcgacttcacggcacgatggaatgtcgcaaccaaatgcattgtccgctttgatgatgaggagagtcatgccactgctaaccattggcgatcaattccccgcctaccagctcaccgctctcatcggcggtgacctgtccaaggtcgacgccaagcagcccggcgactacttcaccactatcaccagtgacgaacacccaggcaagtggcgggtggtgttcttttggccgaaagacttcacgttcgtgtgccctaccgagatcgcggcgttcagcaagctcaatgacgagttcgaggaccgcgacgcccagatcctgggggtttcgattgacagcgaattcgcgcatttccagtggcgtgcacagcacaacgacctcaaaacgttacccttcccgatgctctccgacatcaagcgcgaactcagccaagccgcaggtgtcctcaacgccgacggtgtggccgaccgcgtgacctttatcgtcgaccccaacaacgagatccagttcgtctcggccaccgccggttcggtgggacgcaacgtcgatgaggtactgcgagtgctcgacgccctccagtccgacgagctgtgcgcatgcaactggcgcaagggcgacccgacgctagacgctggcgaactcctcaaggcttcggcctaa |
| CJH63- -30 T | gagaccggcttccgaccaccgctcgccgcaacgtcgactggctcatatcgagaatgcttgcggcactgctgaaccactgctttgccgccaccgcggcgaacgcgcgaagcccggccacggccggctagcacctcttggcggcgatgccgataaatatggtgtgatatatcacctttgcTtgacagcgacttcacggcacgatggaatgtcgcaaccaaatgcattgtccgctttgatgatgaggagagtcatgccactgctaaccattggcgatcaattccccgcctaccagctcaccgctctcatcggcggtgacctgtccaaggtcgacgccaagcagcccggcgactacttcaccactatcaccagtgacgaacacccaggcaagtggcgggtggtgttcttttggccgaaagacttcacgttcgtgtgccctaccgagatcgcggcgttcagcaagctcaatgacgagttcgaggaccgcgacgcccagatcctgggggtttcgattgacagcgaattcgcgcatttccagtggcgtgcacagcacaacgacctcaaaacgttacccttcccgatgctctccgacatcaagcgcgaactcagccaagccgcaggtgtcctcaacgccgacggtgtggccgaccgcgtgacctttatcgtcgaccccaacaacgagatccagttcgtctcggccaccgccggttcggtgggacgcaacgtcgatgaggtactgcgagtgctcgacgccctccagtccgacgagctgtgcgcatgcaactggcgcaagggcgacccgacgctagacgctggcgaactcctcaaggcttcggcctaa |
| CJH64- -15 T | gagaccggcttccgaccaccgctcgccgcaacgtcgactggctcatatcgagaatgcttgcggcactgctgaaccactgctttgccgccaccgcggcgaacgcgcgaagcccggccacggccggctagcacctcttggcggcgatgccgataaatatggtgtgatatatcacctttgcctgacagcgacttcaTggcacgatggaatgtcgcaaccaaatgcattgtccgctttgatgatgaggagagtcatgccactgctaaccattggcgatcaattccccgcctaccagctcaccgctctcatcggcggtgacctgtccaaggtcgacgccaagcagcccggcgactacttcaccactatcaccagtgacgaacacccaggcaagtggcgggtggtgttcttttggccgaaagacttcacgttcgtgtgccctaccgagatcgcggcgttcagcaagctcaatgacgagttcgaggaccgcgacgcccagatcctgggggtttcgattgacagcgaattcgcgcatttccagtggcgtgcacagcacaacgacctcaaaacgttacccttcccgatgctctccgacatcaagcgcgaactcagccaagccgcaggtgtcctcaacgccgacggtgtggccgaccgcgtgacctttatcgtcgaccccaacaacgagatccagttcgtctcggccaccgccggttcggtgggacgcaacgtcgatgaggtactgcgagtgctcgacgccctccagtccgacgagctgtgcgcatgcaactggcgcaagggcgacccgacgctagacgctggcgaactcctcaaggcttcggcctaa |
| CJH65- -12 T | gagaccggcttccgaccaccgctcgccgcaacgtcgactggctcatatcgagaatgcttgcggcactgctgaaccactgctttgccgccaccgcggcgaacgcgcgaagcccggccacggccggctagcacctcttggcggcgatgccgataaatatggtgtgatatatcacctttgcctgacagcgacttcacggTacgatggaatgtcgcaaccaaatgcattgtccgctttgatgatgaggagagtcatgccactgctaaccattggcgatcaattccccgcctaccagctcaccgctctcatcggcggtgacctgtccaaggtcgacgccaagcagcccggcgactacttcaccactatcaccagtgacgaacacccaggcaagtggcgggtggtgttcttttggccgaaagacttcacgttcgtgtgccctaccgagatcgcggcgttcagcaagctcaatgacgagttcgaggaccgcgacgcccagatcctgggggtttcgattgacagcgaattcgcgcatttccagtggcgtgcacagcacaacgacctcaaaacgttacccttcccgatgctctccgacatcaagcgcgaactcagccaagccgcaggtgtcctcaacgccgacggtgtggccgaccgcgtgacctttatcgtcgaccccaacaacgagatccagttcgtctcggccaccgccggttcggtgggacgcaacgtcgatgaggtactgcgagtgctcgacgccctccagtccgacgagctgtgcgcatgcaactggcgcaagggcgacccgacgctagacgctggcgaactcctcaaggcttcggcctaa |
| CJH66- -10 T | gagaccggcttccgaccaccgctcgccgcaacgtcgactggctcatatcgagaatgcttgcggcactgctgaaccactgctttgccgccaccgcggcgaacgcgcgaagcccggccacggccggctagcacctcttggcggcgatgccgataaatatggtgtgatatatcacctttgcctgacagcgacttcacggcatgatggaatgtcgcaaccaaatgcattgtccgctttgatgatgaggagagtcatgccactgctaaccattggcgatcaattccccgcctaccagctcaccgctctcatcggcggtgacctgtccaaggtcgacgccaagcagcccggcgactacttcaccactatcaccagtgacgaacacccaggcaagtggcgggtggtgttcttttggccgaaagacttcacgttcgtgtgccctaccgagatcgcggcgttcagcaagctcaatgacgagttcgaggaccgcgacgcccagatcctgggggtttcgattgacagcgaattcgcgcatttccagtggcgtgcacagcacaacgacctcaaaacgttacccttcccgatgctctccgacatcaagcgcgaactcagccaagccgcaggtgtcctcaacgccgacggtgtggccgaccgcgtgacctttatcgtcgaccccaacaacgagatccagttcgtctcggccaccgccggttcggtgggacgcaacgtcgatgaggtactgcgagtgctcgacgccctccagtccgacgagctgtgcgcatgcaactggcgcaagggcgacccgacgctagacgctggcgaactcctcaaggcttcggcctaa |
| CJH67- -9 A | gagaccggcttccgaccaccgctcgccgcaacgtcgactggctcatatcgagaatgcttgcggcactgctgaaccactgctttgccgccaccgcggcgaacgcgcgaagcccggccacggccggctagcacctcttggcggcgatgccgataaatatggtgtgatatatcacctttgcctgacagcgacttcacggcacAatggaatgtcgcaaccaaatgcattgtccgctttgatgatgaggagagtcatgccactgctaaccattggcgatcaattccccgcctaccagctcaccgctctcatcggcggtgacctgtccaaggtcgacgccaagcagcccggcgactacttcaccactatcaccagtgacgaacacccaggcaagtggcgggtggtgttcttttggccgaaagacttcacgttcgtgtgccctaccgagatcgcggcgttcagcaagctcaatgacgagttcgaggaccgcgacgcccagatcctgggggtttcgattgacagcgaattcgcgcatttccagtggcgtgcacagcacaacgacctcaaaacgttacccttcccgatgctctccgacatcaagcgcgaactcagccaagccgcaggtgtcctcaacgccgacggtgtggccgaccgcgtgacctttatcgtcgaccccaacaacgagatccagttcgtctcggccaccgccggttcggtgggacgcaacgtcgatgaggtactgcgagtgctcgacgccctccagtccgacgagctgtgcgcatgcaactggcgcaagggcgacccgacgctagacgctggcgaactcctcaaggcttcggcctaa |
| CJH68- -6 A | gagaccggcttccgaccaccgctcgccgcaacgtcgactggctcatatcgagaatgcttgcggcactgctgaaccactgctttgccgccaccgcggcgaacgcgcgaagcccggccacggccggctagcacctcttggcggcgatgccgataaatatggtgtgatatatcacctttgcctgacagcgacttcacggcacgatAgaatgtcgcaaccaaatgcattgtccgctttgatgatgaggagagtcatgccactgctaaccattggcgatcaattccccgcctaccagctcaccgctctcatcggcggtgacctgtccaaggtcgacgccaagcagcccggcgactacttcaccactatcaccagtgacgaacacccaggcaagtggcgggtggtgttcttttggccgaaagacttcacgttcgtgtgccctaccgagatcgcggcgttcagcaagctcaatgacgagttcgaggaccgcgacgcccagatcctgggggtttcgattgacagcgaattcgcgcatttccagtggcgtgcacagcacaacgacctcaaaacgttacccttcccgatgctctccgacatcaagcgcgaactcagccaagccgcaggtgtcctcaacgccgacggtgtggccgaccgcgtgacctttatcgtcgaccccaacaacgagatccagttcgtctcggccaccgccggttcggtgggacgcaacgtcgatgaggtactgcgagtgctcgacgccctccagtccgacgagctgtgcgcatgcaactggcgcaagggcgacccgacgctagacgctggcgaactcctcaaggcttcggcctaa |
| CJH69- 2 TCA | gagaccggcttccgaccaccgctcgccgcaacgtcgactggctcatatcgagaatgcttgcggcactgctgaaccactgctttgccgccaccgcggcgaacgcgcgaagcccggccacggccggctagcacctcttggcggcgatgccgataaatatggtgtgatatatcacctttgcctgacagcgacttcacggcacgatggaatgtcgcaaccaaatgcattgtccgctttgatgatgaggagagtcatgTcactgctaaccattggcgatcaattccccgcctaccagctcaccgctctcatcggcggtgacctgtccaaggtcgacgccaagcagcccggcgactacttcaccactatcaccagtgacgaacacccaggcaagtggcgggtggtgttcttttggccgaaagacttcacgttcgtgtgccctaccgagatcgcggcgttcagcaagctcaatgacgagttcgaggaccgcgacgcccagatcctgggggtttcgattgacagcgaattcgcgcatttccagtggcgtgcacagcacaacgacctcaaaacgttacccttcccgatgctctccgacatcaagcgcgaactcagccaagccgcaggtgtcctcaacgccgacggtgtggccgaccgcgtgacctttatcgtcgaccccaacaacgagatccagttcgtctcggccaccgccggttcggtgggacgcaacgtcgatgaggtactgcgagtgctcgacgccctccagtccgacgagctgtgcgcatgcaactggcgcaagggcgacccgacgctagacgctggcgaactcctcaaggcttcggcctaa |
| CJH70- 3AAG | gagaccggcttccgaccaccgctcgccgcaacgtcgactggctcatatcgagaatgcttgcggcactgctgaaccactgctttgccgccaccgcggcgaacgcgcgaagcccggccacggccggctagcacctcttggcggcgatgccgataaatatggtgtgatatatcacctttgcctgacagcgacttcacggcacgatggaatgtcgcaaccaaatgcattgtccgctttgatgatgaggagagtcatgccaAAgctaaccattggcgatcaattccccgcctaccagctcaccgctctcatcggcggtgacctgtccaaggtcgacgccaagcagcccggcgactacttcaccactatcaccagtgacgaacacccaggcaagtggcgggtggtgttcttttggccgaaagacttcacgttcgtgtgccctaccgagatcgcggcgttcagcaagctcaatgacgagttcgaggaccgcgacgcccagatcctgggggtttcgattgacagcgaattcgcgcatttccagtggcgtgcacagcacaacgacctcaaaacgttacccttcccgatgctctccgacatcaagcgcgaactcagccaagccgcaggtgtcctcaacgccgacggtgtggccgaccgcgtgacctttatcgtcgaccccaacaacgagatccagttcgtctcggccaccgccggttcggtgggacgcaacgtcgatgaggtactgcgagtgctcgacgccctccagtccgacgagctgtgcgcatgcaactggcgcaagggcgacccgacgctagacgctggcgaactcctcaaggcttcggcctaa |
| CJH71- 5ATC | gagaccggcttccgaccaccgctcgccgcaacgtcgactggctcatatcgagaatgcttgcggcactgctgaaccactgctttgccgccaccgcggcgaacgcgcgaagcccggccacggccggctagcacctcttggcggcgatgccgataaatatggtgtgatatatcacctttgcctgacagcgacttcacggcacgatggaatgtcgcaaccaaatgcattgtccgctttgatgatgaggagagtcatgccactgctaaTcattggcgatcaattccccgcctaccagctcaccgctctcatcggcggtgacctgtccaaggtcgacgccaagcagcccggcgactacttcaccactatcaccagtgacgaacacccaggcaagtggcgggtggtgttcttttggccgaaagacttcacgttcgtgtgccctaccgagatcgcggcgttcagcaagctcaatgacgagttcgaggaccgcgacgcccagatcctgggggtttcgattgacagcgaattcgcgcatttccagtggcgtgcacagcacaacgacctcaaaacgttacccttcccgatgctctccgacatcaagcgcgaactcagccaagccgcaggtgtcctcaacgccgacggtgtggccgaccgcgtgacctttatcgtcgaccccaacaacgagatccagttcgtctcggccaccgccggttcggtgggacgcaacgtcgatgaggtactgcgagtgctcgacgccctccagtccgacgagctgtgcgcatgcaactggcgcaagggcgacccgacgctagacgctggcgaactcctcaaggcttcggcctaa |
| W1 | AGCCCGATGAGGTCTATTGGGGCAAGGAAGCCACCTGGCTCGGCGATGAGCGTTACAGCGGTAAGCGGGATCTGGAGAACCCGCTGGCCGCGGTGCAGATGGGGCTGATCTACGTGAACCCGGAGGGGCCGAACGGCAACCCGGACCCCATGGCCGCGGCGGTCGACATTCGCGAGACGTTTCGGCGCATGGCCATGAACGACGTCGAAACAGCGGCGCTGATCGTCGGCGGTCACACTTTCGGTAAGACCCATGGCGCCGGCCCGGCCGATCTGGTCGGCCCCGAACCCGAGGCTGCTCCGCTGGAGCAGATGGGCTTGGGCTGGAAGAGCTCGTATGGCACCGGAACCGGTAAGGACGCGATCACCAGCGGCATCGAGGTCGTATGGACGAACACCCCGACGAAATGGGACAACAGTTTCCTCGAGATCCTGTACGGCTACGAGTGGGAGCTGACGAAGAGCCCTGCTGGCGCTTGGCAATACACCGCCAAGGACGGCGCCGGTGCCGGCACCATCCCGGACCCGTTCGGCGGGCCAGGGCGCTCCCCGACGATGCTGGCCACTGACCTCTCGCTGCGGGTGGATCCGATCTATGAGCGGATCACGCGTCGCTGGCTGGAACACCCCGAGGAATTGGCCGACGAGTTCGCCAAGGCCTGGTACAAGCTGATCCACCGAGACATGGGTCCCGTTGCGAGATACCTTGGGCCGCTGGTCCCCAAGCAGACCCTGCTGTGGCAGGATCCGGTCCCTGCGGTCAGCCACGACCTCGTCGG |
| W2 | GGGCACGGCGGCGGTTGGTGTCGATGATCGCGGCGGGAAGATCCGCGTCGATCCACTTGGCGCCATGGAAGGCAGAAGCCGAGTAGCCGGCCAGCACGCCGCGGCGGCGCGAGCGCAGCCACAGCGCTTTTGCACGCAATTGCGCGGTCAGTTCCACACCCTGCGGCACGTACACGTCTTTATGTAGCGCGACATACCTGCTGCGCAATTCGTAGGGCGTCAATACACCCGCAGCCAGGGCCTCGCTGCCCAGAAAGGGATCCGTCATGGTCGAAGTGTGCTGAGTCACACCGACAAACGTCACGAGCGTAACCCCAGTGCGAAAGTTCCCGCCGGAAATCGCAGCCACGTTACGCTCGTGGACATACCGATTTCGGCCCGGCCGCGGCGAGACGATAGGTTGTCGGGGTGACTGCCACAGCCACTGAAGGGGCCAAACCCCCATTCGTATCCCGTTCAGTCCTGGTTACCGGAGGAAACCGGGGGATCGGGCTGGCGATCGCACAGCGGCTGGCTGCCGACGGCCACAAGGTGGCCGTCACCCACCGTGGATCCGGAGCGCCAAAGGGGCTGTTTGGCGTCGAATGTGACGTCACCGACAGCGACGCCGTCGATCGCGCCTTCACGGCGGTAGAAGAGCACCAGGGTCCGGTCGAGGTGCTGGTGTCCAACGCCGGCCTATCCGCGGACGCATTCCTCATGCGGATGACCGAGGAAAAGTTCGAGAAGGTCATCAACGCCAACCTCACCGGGGCGTTCCGGGTGGCTCAACGGGCATCGCGCAGCATGCAGCG |
| W8 | gagaccggcttccgaccaccgctcgccgcaacgtcgactggctcatatcgagaatgcttgcggcactgctgaaccactgctttgccgccaccgcggcgaacgcgcgaagcccggccacggccggctagcacctcttggcggcgatgccgataaatatggtgtgatatatcacctttgcctgacagcgacttcacggcacgatggaatgtcgcaaccaaatgcattgtccgctttgatgatgaggagagtcatgccactgctaaccattggcgatcaattccccgcctaccagctcaccgctctcatcggcggtgacctgtccaaggtcgacgccaagcagcccggcgactacttcaccactatcaccagtgacgaacacccaggcaagtggcgggtggtgttcttttggccgaaagacttcacgttcgtgtgccctaccgagatcgcggcgttcagcaagctcaatgacgagttcgaggaccgcgacgcccagatcctgggggtttcgattgacagcgaattcgcgcatttccagtggcgtgcacagcacaacgacctcaaaacgttacccttcccgatgctctccgacatcaagcgcgaactcagccaagccgcaggtgtcctcaacgccgacggtgtggccgaccgcgtgacctttatcgtcgaccccaacaacgagatccagttcgtctcggccaccgccggttcggtgggacgcaacgtcgatgaggtactgcgagtgctcgacgccctccagtccgacgagctgtgcgcatgcaactggcgcaagggcgacccgacgctagacgctggcgaactcctcaaggcttcggcctaa |
| Q510Q | AATATCTGGTCCGCTTGCACGAGGGTCAGACCACGATGACCGTTCCGGGCGGCGTCGAGGTGCCGGTGGAAACCGACGACATCGACCACTTCGGCAACCGCCGCCTGCGTACGGTCGGCGAGCTGATCCAAAACCAGATCCGGGTCGGCATGTCGCGGATGGAGCGGGTGGTCCGGGAGCGGATGACCACCCAGGACGTGGAGGCGATCACACCGCAGACGTTGATCAACATCCGGCCGGTGGTCGCCGCGATCAAGGAGTTCTTCGGCACCAGCCAACTGAGCCAATTCATGGACCAGAACAACCCGCTGTCGGGGTTGACCCACAAGCGCCGACTGTCGGCGCTGGGGCCCGGCGGTCTGTCACGTGAGCGTGCCGGGCTGGAGGTCCGCGACGTGCACCCGTCGCACTACGGCCGGATGTGCCCGATCGAAACCCCTGAGGGGCCCAACATCGGTCTGATCGGCTCGCTGTCGGTGTACGCGCGGGTCAACCCGTTCGGGTTCATCGAAACGCCGTACCGCAAGGTGGTCGACGGCGTGGTTAGCGACGAGATCGTGTACCTGAC |
| F514F | AATATCTGGTCCGCTTGCACGAGGGTCAGACCACGATGACCGTTCCGGGCGGCGTCGAGGTGCCGGTGGAAACCGACGACATCGACCACTTCGGCAACCGCCGCCTGCGTACGGTCGGCGAGCTGATCCAAAACCAGATCCGGGTCGGCATGTCGCGGATGGAGCGGGTGGTCCGGGAGCGGATGACCACCCAGGACGTGGAGGCGATCACACCGCAGACGTTGATCAACATCCGGCCGGTGGTCGCCGCGATCAAGGAGTTCTTCGGCACCAGCCAGCTGAGCCAATTTATGGACCAGAACAACCCGCTGTCGGGGTTGACCCACAAGCGCCGACTGTCGGCGCTGGGGCCCGGCGGTCTGTCACGTGAGCGTGCCGGGCTGGAGGTCCGCGACGTGCACCCGTCGCACTACGGCCGGATGTGCCCGATCGAAACCCCTGAGGGGCCCAACATCGGTCTGATCGGCTCGCTGTCGGTGTACGCGCGGGTCAACCCGTTCGGGTTCATCGAAACGCCGTACCGCAAGGTGGTCGACGGCGTGGTTAGCGACGAGATCGTGTAC |
| D516D | AATATCTGGTCCGCTTGCACGAGGGTCAGACCACGATGACCGTTCCGGGCGGCGTCGAGGTGCCGGTGGAAACCGACGACATCGACCACTTCGGCAACCGCCGCCTGCGTACGGTCGGCGAGCTGATCCAAAACCAGATCCGGGTCGGCATGTCGCGGATGGAGCGGGTGGTCCGGGAGCGGATGACCACCCAGGACGTGGAGGCGATCACACCGCAGACGTTGATCAACATCCGGCCGGTGGTCGCCGCGATCAAGGAGTTCTTCGGCACCAGCCAGCTGAGCCAATTCATGGATCAGAACAACCCGCTGTCGGGGTTGACCCACAAGCGCCGACTGTCGGCGCTGGGGCCCGGCGGTCTGTCACGTGAGCGTGCCGGGCTGGAGGTCCGCGACGTGCACCCGTCGCACTACGGCCGGATGTGCCCGATCGAAACCCCTGAGGGGCCCAACATCGGTCTGATCGGCTCGCTGTCGGTGTACGCGCGGGTCAACCCGTTCGGGTTCATCGAAACGCCGTACCGCAAGGTGGTCGACGGCGTGGTTAGCGACGAGATCGTGTAC |
| L533L | AATATCTGGTCCGCTTGCACGAGGGTCAGACCACGATGACCGTTCCGGGCGGCGTCGAGGTGCCGGTGGAAACCGACGACATCGACCACTTCGGCAACCGCCGCCTGCGTACGGTCGGCGAGCTGATCCAAAACCAGATCCGGGTCGGCATGTCGCGGATGGAGCGGGTGGTCCGGGAGCGGATGACCACCCAGGACGTGGAGGCGATCACACCGCAGACGTTGATCAACATCCGGCCGGTGGTCGCCGCGATCAAGGAGTTCTTCGGCACCAGCCAGCTGAGCCAATTCATGGACCAGAACAACCCGCTGTCGGGGTTGACCCACAAGCGCCGACTGTCGGCGTTGGGGCCCGGCGGTCTGTCACGTGAGCGTGCCGGGCTGGAGGTCCGCGACGTGCACCCGTCGCACTACGGCCGGATGTGCCCGATCGAAACCCCTGAGGGGCCCAACATCGGTCTGATCGGCTCGCTGTCGGTGTACGCGCGGGTCAACCCGTTCGGGTTCATCGAAACGCCGTACCGCAAGGTGGTCGACGGCGTGGTTAGCGACGAGATCGTGTAC |
| Q513Q | AATATCTGGTCCGCTTGCACGAGGGTCAGACCACGATGACCGTTCCGGGCGGCGTCGAGGTGCCGGTGGAAACCGACGACATCGACCACTTCGGCAACCGCCGCCTGCGTACGGTCGGCGAGCTGATCCAAAACCAGATCCGGGTCGGCATGTCGCGGATGGAGCGGGTGGTCCGGGAGCGGATGACCACCCAGGACGTGGAGGCGATCACACCGCAGACGTTGATCAACATCCGGCCGGTGGTCGCCGCGATCAAGGAGTTCTTCGGCACCAGCCAGCTGAGCCAGTTCATGGACCAGAACAACCCGCTGTCGGGGTTGACCCACAAGCGCCGACTGTCGGCGCTGGGGCCCGGCGGTCTGTCACGTGAGCGTGCCGGGCTGGAGGTCCGCGACGTGCACCCGTCGCACTACGGCCGGATGTGCCCGATCGAAACCCCTGAGGGGCCCAACATCGGTCTGATCGGCTCGCTGTCGGTGTACGCGCGGGTCAACCCGTTCGGGTTCATCGAAACGCCGTACCGCAAGGTGGTCGACGGCGTGGTTAGCGACGAGATCGTGTAC |

**Table S4: Primer sequences used in DNA Sequencing and digital droplet PCR.**

| Method | Primer name | Oligonucleotide Sequence (5’-3’) |
| --- | --- | --- |
| Sequencing | rpoB-F-Seq | 5’-GACGACATCGACCACTTC-3’ |
|  | rpoB-R-Seq | 5’-CCATGTAGTCCACCTCAGA-3’ |
|  | katG-F-Seq | 5’-ATGAGCGTTACAGCGGTA-3’ |
|  | katG-R-Seq | 5’-CCATGTCTCGGTGGATCA-3’ |
|  | inhA-F-Seq | 5’-AAGGCAGAAGCCGAGTAG-3’ |
|  | inhA-R-Seq | 5’-GAGGTTGGCGTTGATGAC-3’ |
|  | ahpC-F-Seq | 5’-TGCCGATAAATATGGTGTGA-3’ |
|  | ahpC-R-Seq | 5’-CGAAGCCTTGAGGAGTTC-3’ |
| digital droplet PCR | rpoB-DF | 5’-CACCGCAGACGTTGATCAACAT-3’ |
|  | rpoB-DR | 5’-TGCACGTCGCGGACCTC-3’ |
|  | rpoB-DP | 5’ -FAM-CGCGATCAAGGAGTTCTTCGGCA-BHQ1-3’ |
|  | katG-DF | 5’-CTGGAAGAGCTCGTATGGCA-3’ |
|  | katG-DR | 5’-GTCAGCTCCCACTCGTAGCC-3’ |
|  | katG-DP | 5’ -FAM-TCGTATGGACGAACACCCCGA-BHQ1-3’ |
|  | inhA-DF | 5’-ATCGCAGCCACGTTACGCT-3’ |
|  | inhA-DR | 5’-TCCTCCGGTAACCAGGACTGA-3’ |
|  | inhA-DP | 5’ -FAM-CCCTTCAGTGGCTGTGGCAGTCA-BHQ1-3’ |
|  | ahpC-DF1 | 5’-TAGCACCTCTTGGCGGCGAT-3’ |
|  | ahpC-DR1 | 5’-TGGTTAGCAGTGGCATGACTCT-3’ |
|  | ahpC-DP1 | 5’ -FAM-CGACATTCCATCGTGCCGTGAAGT-BHQ1-3’ |
|  | ahpC-DF2 | 5’-CGATGCCGATAAATATGGTGTG-3’ |
|  | ahpC-DR2 | 5’-GACCTTGGACAGGTCACCGC-3’ |
|  | ahpC-DP2 | 5’ -FAM-CGCCAATGGTTAGCAGTGGCATGAC-BHQ1-3’ |
|  | ahpC-DF3 | 5’-CGACTTCACGGCACGATGGA-3’ |
|  | ahpC-DR3 | 5’-GTCGACCTTGGACAGGTCAC-3’ |
|  | ahpC-DP3 | 5’ -FAM-GTGAGCTGGTAGGCGGGGAATTGAT-BHQ1-3 |

**Table S5: Tm values obtained from individual single plex PCRs to serve as a reference for interpretating results of tested samples.**

| Probe | Amino acid/nucleotide change | T_m,S_⃰ of probe | | T_m,W_⃰ of probe | |
| --- | --- | --- | --- | --- | --- |
|  |  | Mean T_m_ (℃) | SD (℃) | Mean T_m_ (℃) | SD (℃) |
| rpoB-P1 | Leu511Pro | 69.2 | 0.10 | 73.2 | 0.12 |
|  | Gln 513Pro | 62.8 | 0.11 |  |  |
|  | Gln 513Lys | 58.8 | 0.06 |  |  |
|  | Gln 513Leu | 61.6 | 0.13 |  |  |
|  | Asp516Val | 68.8 | 0.15 |  |  |
|  | Asp516Tyr | 69.6 | 0.13 |  |  |
|  | Asp516Gly | 68.4 | 0.08 |  |  |
|  | 517-518 Deletion | 69.6 | 0.13 |  |  |
| rpoB-P2 | Ser531Leu | 72.0 | 0.14 | 79.6 | 0.14 |
|  | Ser531Trp | 70.8 | 0.10 |  |  |
|  | Ser531Phe | 68.4 | 0.11 |  |  |
|  | Leu533Pro | 76.0 | 0.12 |  |  |
| rpoB-P3 | Ser 522Leu | 74.4 | 0.08 | 81.6 | 0.15 |
|  | His526Asp | 74.4 | 0.13 |  |  |
|  | His526Tyr | 74.8 | 0.04 |  |  |
|  | His526Leu | 77.6 | 0.18 |  |  |
|  | His526Arg | 77.2 | 0.13 |  |  |
| rpoB-P4 | 510-512 Deletion | 62.0 | 0.07 | NONE | / |
| katG-P | Ser315Thr | 69.6 | 0.11 | 72.8 | 0.11 |
|  | Ser315Asn | 70.0 | 0.17 |  |  |
|  | Gly316Ser | 70.4 | 0.16 |  |  |
|  | Gly316Asp | 70.4 | 0.14 |  |  |
| ahpC-P1 | Pro2Ser | 62.0 | 0.08 | 68.8 | 0.18 |
|  | Leu2Lys | 58.8 | 0.14 |  |  |
|  | Thr5Ile | 62.4 | 0.07 |  |  |
| ahpC-P2 | G(-46)A | 62.8 | 0.15 | 66.8 | 0.12 |
|  | T(-44)A | 63.2 | 0.13 |  |  |
|  | T(-40)C | 64.4 | 0.07 |  |  |
|  | C(-39)T | 60.8 | 0.08 |  |  |
|  | T(-34)C | 62.8 | 0.12 |  |  |
|  | T(-34)A | 63.6 | 0.15 |  |  |
|  | G(-32)A | 63.2 | 0.10 |  |  |
|  | C(-30)T | 60.8 | 0.13 |  |  |
| ahpC-P3 | C(-15)T | 68.0 | 0.18 | 72.0 | 0.13 |
|  | C(-12)T | 65.2 | 0.13 |  |  |
|  | C(-10)T | 65.2 | 0.09 |  |  |
|  | G(-9)A | 67.6 | 0.14 |  |  |
|  | G(-6)A | 67.6 | 0.16 |  |  |
| inhA-P | C(-15)T | 61.2 | 0.07 | 70.8 | 0.16 |
|  | T(-8)A | 64.4 | 0.11 |  |  |

⃰T_m.S_ refers to T_m_ value of mutant sequences, while T_m.W_ refers to T_m_ value of WT. Mean T_m_ and SD values were calculated from the T_m_ values obtained from 10 individual single-plex PCR-MPMA experiments.

**Table S6: T_m_ values obtained from plasmids carrying the five *rpoB* synonymous mutations detected by the multiplex PCR-MPMA assay and interpretation of the results.**

| Probe | Amino acid change | T_m,S_⃰ of probe | | T_m,W_⃰ of probe | | ∆T_m_ (℃) | Interpretation of the result |
| --- | --- | --- | --- | --- | --- | --- | --- |
|  |  | Mean T_m_ (℃) | SD (℃) | Mean T_m_ (℃) | SD (℃) |  |  |
| rpoB-P1 | Gln 510 Gln | 66.8 | 0.11 | 72.7 | 0.16 | 5.9 | Resistant to RIF/False Positive |
|  | Phe 514 Phe | 64.3 | 0.19 |  |  | 8.4 | Resistant to RIF/False Positive |
|  | Asp 516 Asp | 66.0 | 0.11 |  |  | 6.7 | Resistant to RIF/False Positive |
|  | Gln 513 Gln | 65.7 | 0.15 |  |  | 7 | Resistant to RIF/False Positive |
| rpoB-P2 | Leu 533 Leu | 70.8 | 0.11 | 78.8 | 0.27 | 8 | Resistant to RIF/False Positive |

⃰T_m.S_ refers to T_m_ value of synonymous mutant sequences, while T_m.W_ refers to T_m_ value of WT. Mean T_m_ and SD values were calculated from the T_m_ values obtained from three individual Multiplex PCR-MPMA experiments using the relevant *rpoB* probes. ∆T_m_ was determined by calculating the difference in mean T_m_ values of the melting curves between the measured sample and the WT reference control.

**Additional file 2**

**
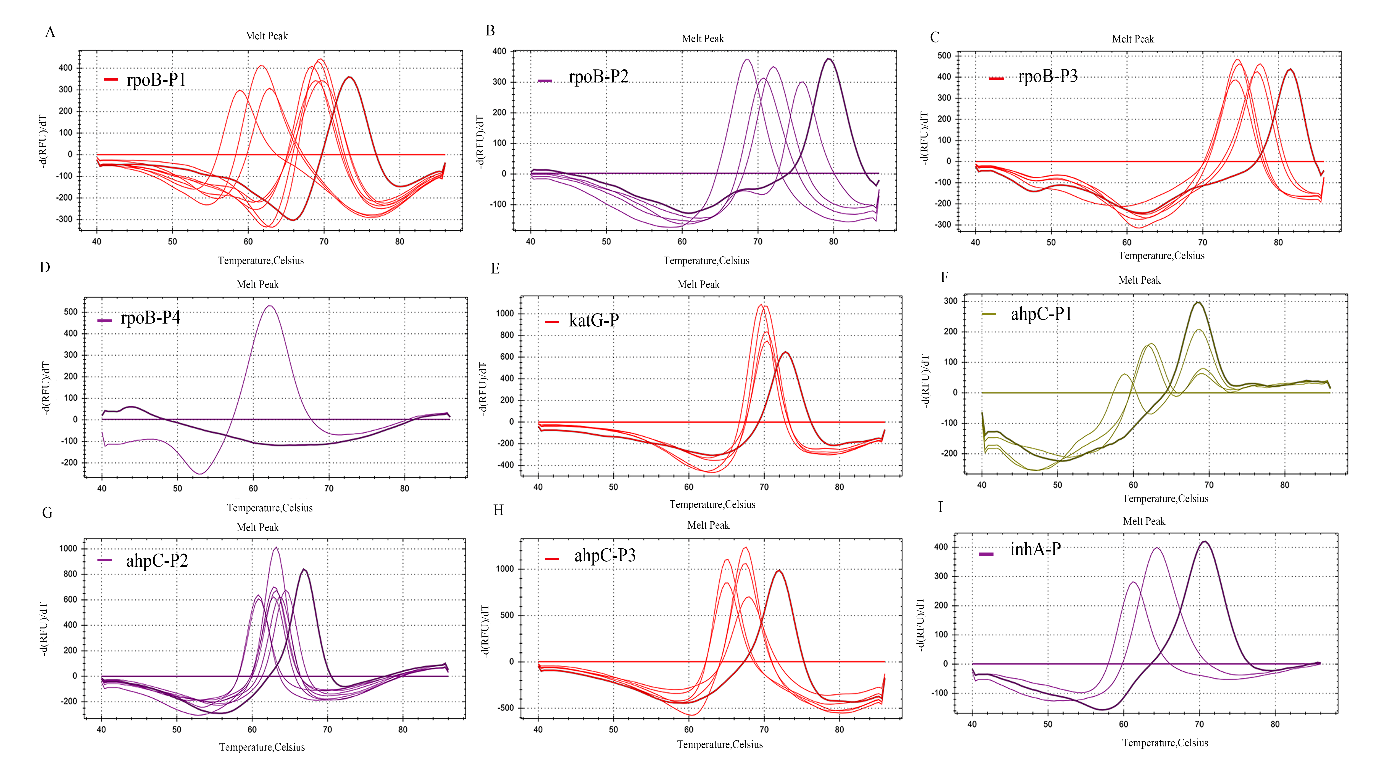
**

**Figure S1. Characteristic melting curves generated by nine single-plex PCRs demonstrating the detection of *M. tuberculosis* RIF (A, B, C, D) and INH (E, F, G, H, I) resistance.** Melting curves of artificial plasmid templates containing WT and the 40 mutant sequences indicative of the relevant drug resistance were each tested by the corresponding single-plex PCR experiments. Drug-susceptible WT targets were identified by the presence of characteristic melting peaks (bold curves) and T_m.W_ values, while drug-resistance mutations in these targets were recognized by a shift in the melting curves and T_m.W_ values away from the WT profiles, as shown by graphs of rpoB-P1 (A), rpoB-P2 (B), rpoB-P3 (C), rpoB-P4 (D), katG-P (E), ahpC-P1 (F), ahpC-P2 (G), ahpC-P3 (H) and inhA-P (I), respectively. Briefly, because of the higher T_m_ values, the melting peaks representing the WT plasmids detected by each probe are always on the rightmost side of the same-colored lines including the mutant plasmids in each graph. Individual melting curves were generated by molecular beacon probes labeled with Texas Red (red), Cy5 (purple) and VIC (green), respectively.


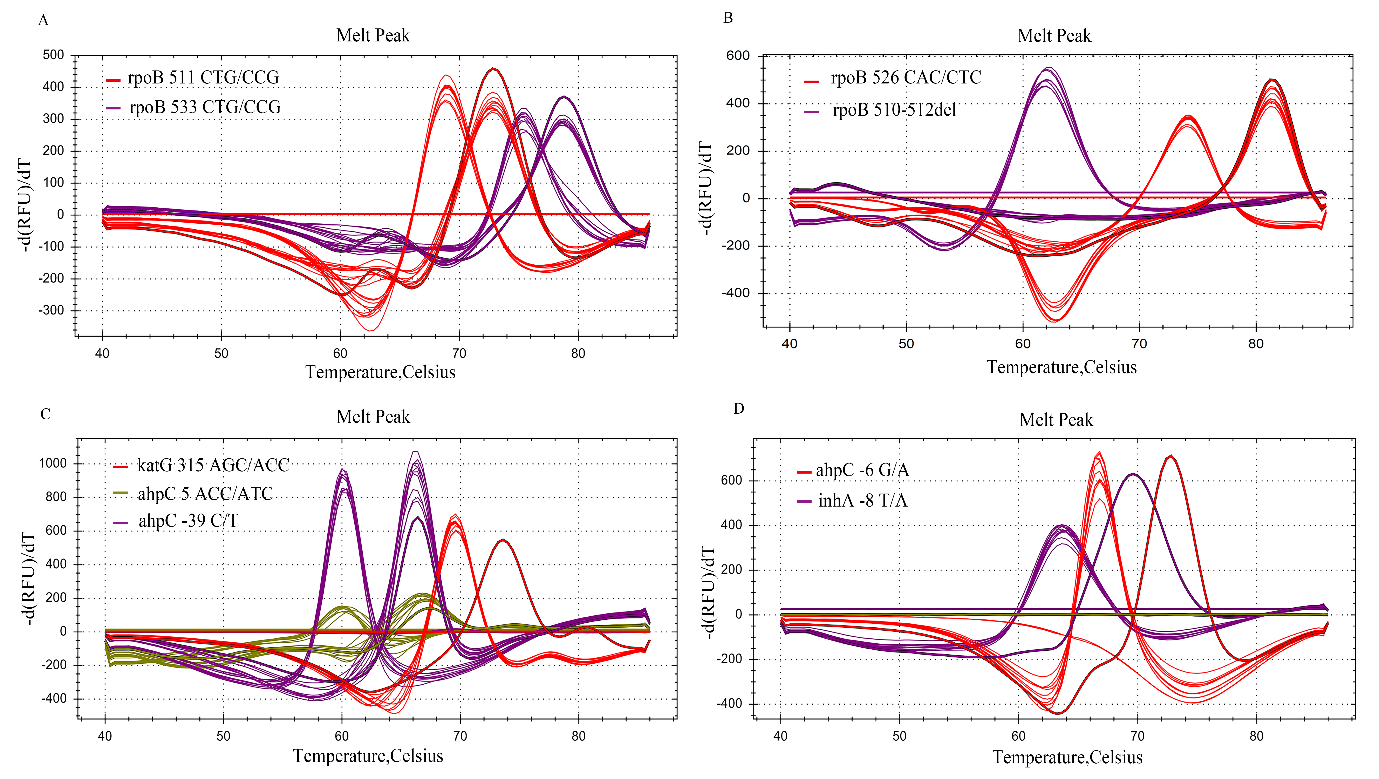


**Figure S2. Determination of the detection limits of the four-tube multiplex PCR-MPMA assay with WT and mutant plasmids.** When serial 10-fold dilutions of the mixtures of WT and mutant reference standard plasmids demonstrated 5000 copies/ml the detection limit of the assay for detecting WT and the desired mutations from the four genes, confirmatory experiments were performed to verify 100% success rates for identifying RIF-resistance (**A, B**; the *rpoB* gene) and INH-resistance (**C, D**; the *katG*, *ahpC* and *inhA* genes) based on 10 replicates of each positive controls.


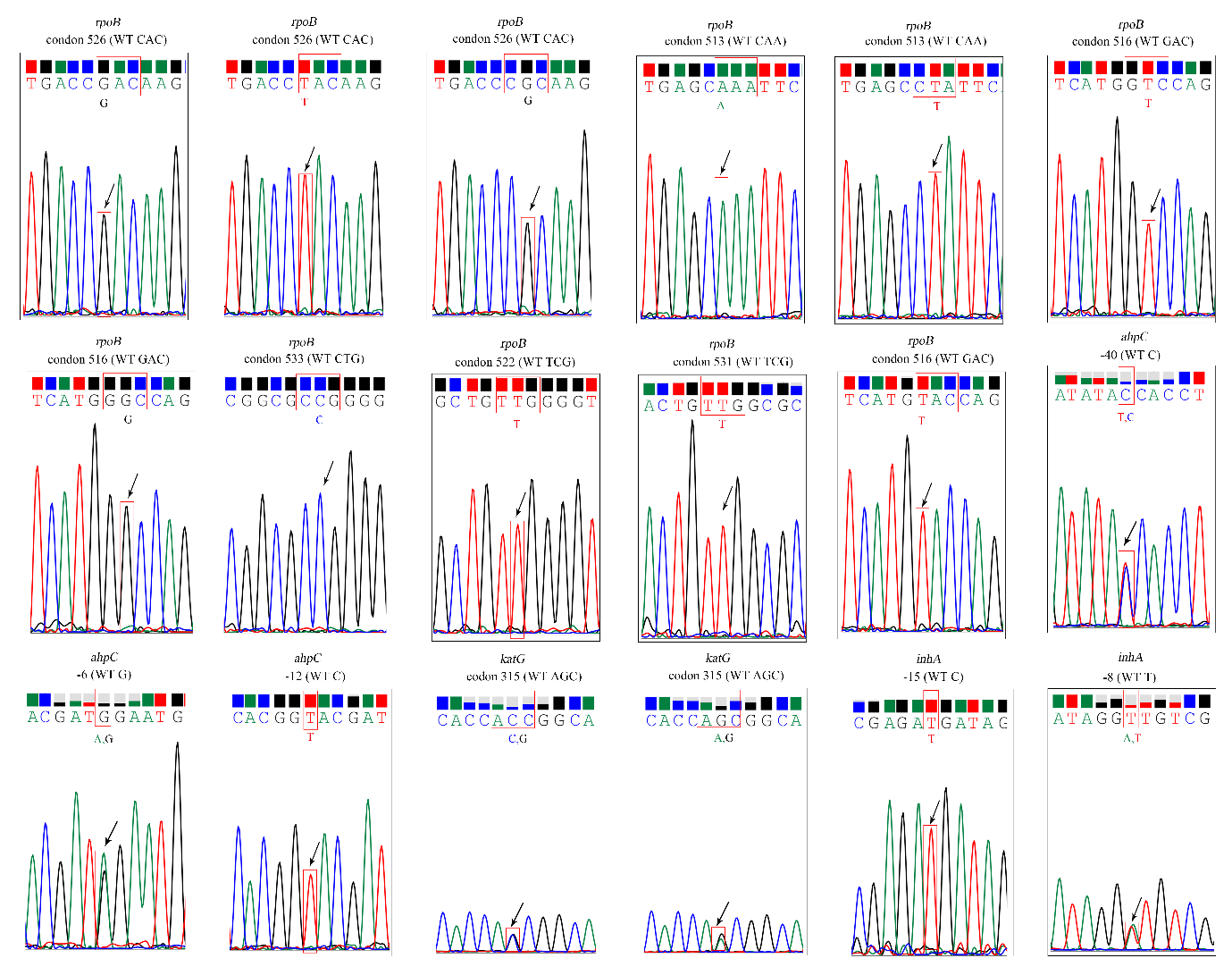


**Figure S3.** **Representative electropherograms obtained by DNA sequencing for RIF- and INH- resistant clinical samples.**


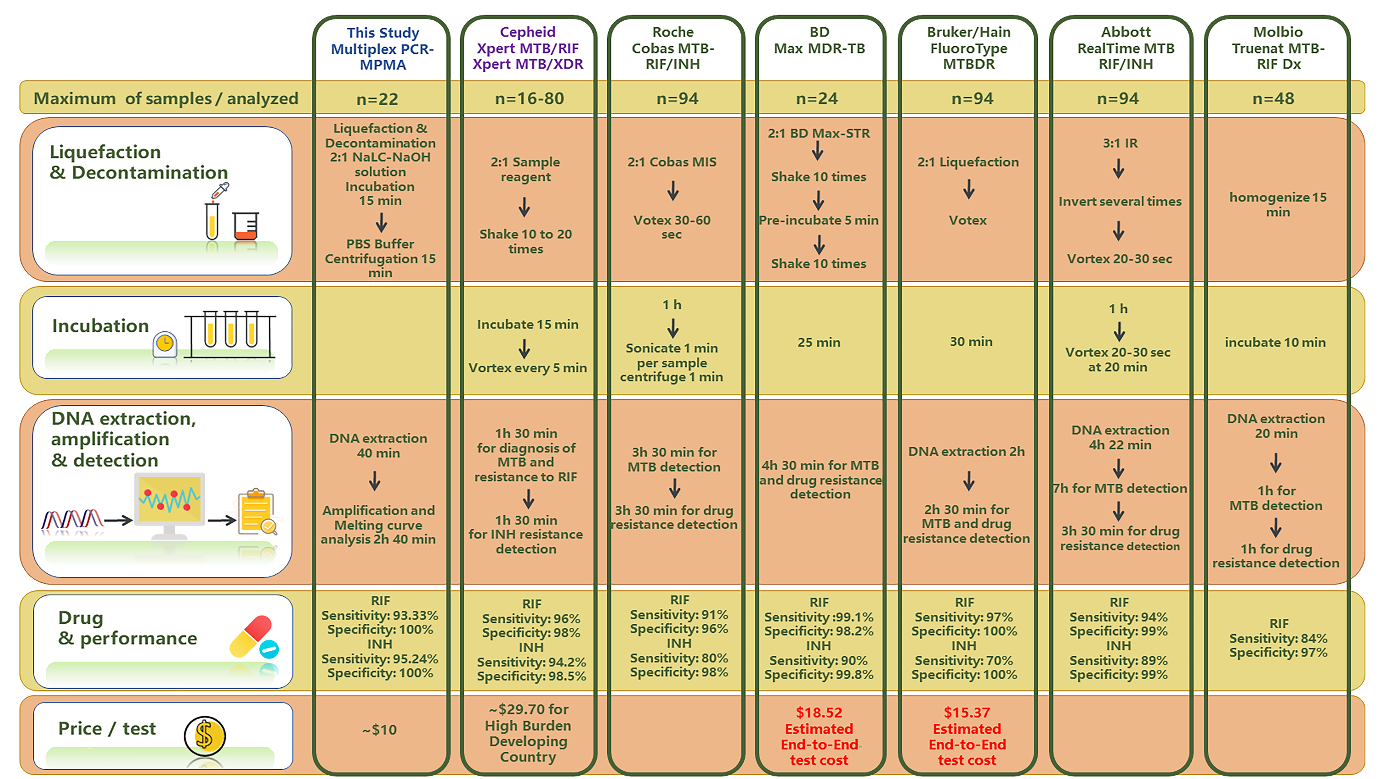


**Figure S4. Comparison of workflow, type of drug resistance detected, diagnostic performance and price between multiplex PCR-MPMA assay and WHO-recommended assay platforms.** Each assay analyzes the number of samples per test as indicated. While the Cepheid, Roche, and BD instruments are noted for their integration, which facilitates a streamlined process from DNA extraction to detection, thereby reducing the likelihood of sample contamination and enhancing biosafety measures. However, this integration often necessitates specialized infrastructure that may not be available in all settings. In contrast, our PCR-MPMA assay, along with several others, requires distinct equipment for each step of the testing process: DNA extraction, PCR amplification, and detection. This segmentation necessitates manual transfer of samples between instruments, a step that could potentially increase the risk of contamination if not managed with stringent biosafety protocols. Despite this, the flexibility in equipment choice allows for adaptation in varied laboratory settings, mitigating the heavy infrastructure investments required by more integrated systems. The WHO classifies Cepheid’s Xpert MTB/RIF and MTB/XDR tests as follow-on diagnostic tests for the detection of RIF and INH resistance. Following the initial diagnostic application of the Xpert MTB/RIF assay, which is utilized for both the diagnosis of TB and the detection of RIF resistance, the Xpert MTB/XDR assay is subsequently administered. This updated assay remarkably extends the diagnostic capabilities of the first version of Xpert MTB/RIF to include the detection of resistance not only to INH, but also to boarder anti-TB medications comprising FQ, injectable second-line drugs (such as amikacin, kanamycin, and capreomycin), and ethionamide, thus providing a comprehensive assessment of MDR-TB. For the cobas MTB-RIF/INH by Roche, RealTime MTB RIF/INH by Abbott, and Truenat MTB-RIF Dx by Molbio a two-step process involving distinct PCR amplification reactions is employed. The initial step aims to identify infection of *M. tuberculosis*, while the subsequent phase is dedicated to the detection of drug resistance. The data pertaining to the operational manuals and technical specifications of the WHO-recommended assays spotlighted in this illustration were collated from references 22, 23, 24 and 36. Pricing information for Cepheid’s MTB/XDR, BD’s Max MDR-TB and Hain’s FluoroType MTBDR was sourced from reference 22. In summary, while most the commercial systems come with significant infrastructure demands, our multiplex PCR-MPMA assay offers a flexible and economically viable option that aligns well with the diverse capabilities of laboratories worldwide. FQ, fluoroquinolones; INH, isoniazid; IR, inactivation reagent; MDR, multidrug-resistant; MIS, microbial inactivation reagent; *M. tuberculosis*, *Mycobacterium tuberculosis*; RIF, rifampicin; STR, sample treatment reagent; TB, tuberculosis; XDR, extensive drug-resistant.
